# Machine learning *v.* traditional regression models predicting treatment outcomes for binge-eating disorder from a randomized controlled trial

**DOI:** 10.1017/S0033291721004748

**Published:** 2023-05

**Authors:** Lauren N. Forrest, Valentina Ivezaj, Carlos M. Grilo

**Affiliations:** 1 Department of Psychiatry, Yale School of Medicine, New Haven, CT, USA; 2 Department of Psychiatry, Penn State College of Medicine, 700 HMC Crescent Road, Hershey, PA 17033, USA

**Keywords:** Behavioral weight loss, binge-eating disorder, eating disorders, machine learning, obesity, prediction, treatment, weight stigma

## Abstract

**Background:**

While effective treatments exist for binge-eating disorder (BED), prediction of treatment outcomes has proven difficult, and few reliable predictors have been identified. Machine learning is a promising method for improving the accuracy of difficult-to-predict outcomes. We compared the accuracy of traditional and machine-learning approaches for predicting BED treatment outcomes.

**Methods:**

Participants were 191 adults with BED in a randomized controlled trial testing 6-month behavioral and stepped-care treatments. Outcomes, determined by independent assessors, were binge-eating (% reduction, abstinence), eating-disorder psychopathology, and weight loss (% loss, ⩾5% loss). Predictors included treatment condition, demographic information, and baseline clinical characteristics. Traditional models were logistic/linear regressions. Machine-learning models were elastic net regressions and random forests. Predictive accuracy was indicated by the area under receiver operator characteristic curve (AUC), root mean square error (RMSE), and *R*^2^. Confidence intervals were used to compare accuracy across models.

**Results:**

Across outcomes, AUC ranged from very poor to fair (0.49–0.73) for logistic regressions, elastic nets, and random forests, with few significant differences across model types. RMSE was significantly lower for elastic nets and random forests *v.* linear regressions but *R*^2^ values were low (0.01–0.23).

**Conclusions:**

Different analytic approaches revealed some predictors of key treatment outcomes, but accuracy was limited. Machine-learning models with unbiased resampling methods provided a minimal advantage over traditional models in predictive accuracy for treatment outcomes.

## Introduction

Binge-eating disorder (BED) is a prevalent eating disorder associated strongly with obesity, elevated psychiatric and medical comorbidities, and psychosocial impairment (Udo & Grilo, 2018, 2019). Specific treatments for BED are known to reduce binge eating (Grilo, 2017; Hilbert et al., 2019) but many patients do not benefit sufficiently; the leading BED treatments result in binge-eating abstinence for only half of patients (Linardon, 2018), and most treatments fail to produce clinically meaningful weight loss (Hilbert et al., 2019).

Prediction of BED treatment outcomes has proven difficult. A number of patient variables have been evaluated as predictors, including – but not limited to – various eating-disorder psychopathology scales/measures as well as specific features such as overvaluation of shape/weight, self-control, depression and negative affect, and psychiatric comorbidity (e.g. Anderson et al., 2020; Grilo, Masheb, & Crosby, 2012a; Grilo, Thompson-Brenner, Shingleton, Thompson, & Franko, 2021; Lydecker & Grilo, in press; see online Supplementary Materials). Research has also tested treatment parameters (Thompson-Brenner et al., 2013) and processes such as rapid response to treatment (⩾65% reduction in binge-eating episodes within the first month of treatment; Grilo, White, Masheb, & Gueorguieva, 2015). To date, however, no reliable predictors of BED outcome (other than rapid response) have been identified (Linardon, Brennan, & de la Piedad Garcia, 2016; Vall & Wade, 2015). One potential reason for the limited ability to predict treatment outcomes – a problem across many fields, not just eating disorders – could be due to reliance on traditional statistical techniques, such as linear/logistic regression. Regression methods assess univariate and linear relations between limited numbers of predictors and outcomes, and this approach (ideally informed by theory) might be poorly matched to the complexity inherent in both psychopathology architecture and treatment mechanisms (Chekroud et al., 2021; King & Resick, 2014). In addition, traditional regression models are subject to overfitting, which can result in the identification of significant predictors that lack generalizability and clinical utility (Dwyer, Falkai, & Koutsouleris, 2018; Poldrack, Huckins, & Varoquaux, 2020).

Recently, machine learning (ML) approaches have been used in attempts to enhance the prediction of hard-to-predict outcomes. ML is an umbrella term for many types of analyses sharing several commonalities. First, ML analyses are inductive, meaning that they rely on patterns in the data to generate and optimize models, as compared to relying on clinicians/researchers specifying models a priori (Kuhn & Johnson, 2013). The algorithms include tuning parameters that identify the model that results in optimal prediction (Kuhn & Johnson, 2013). Second, ML enhances generalizability through cross-validation (i.e. a method to evaluate model effectiveness and generalizability), which can be done through simulations (e.g. bootstrapping), training models on one subset of data and then testing models on a separate subset of data, or a combination of the two (Kuhn & Johnson, 2013). Third, ML algorithms can accommodate large numbers of predictors even with sample sizes in the hundreds (Poldrack et al., 2020). Early applications of ML showed promise in predicting self-injurious behaviors (e.g. Huang, Ribeiro, & Franklin, 2020). Whereas traditional statistical models predicted self-injurious behaviors barely above chance (Franklin et al., 2017), initial ML studies reported excellent prediction (Fox et al., 2019; Huang et al., 2020; Walsh, Ribeiro, & Franklin, 2017). ML has been applied to eating disorders in several studies (Espel-Huynh et al., 2021; Haynos et al., in press; Sadeh-Sharvit, Fitzsimmons-Craft, Taylor, & Yom-Tov, 2020); ML showed increased predictive accuracy for outcomes relative to traditional models in some (Haynos et al., in press) but not other (Espel-Huynh et al., 2021) studies.

Notably, several of the initial ML studies in clinical psychology used random forests paired with a form of resampling called optimism-corrected bootstrapping (Fox et al., 2019; Huang et al., 2020; Walsh et al., 2017). Although random forests are a robust ML method (see online Supplementary Materials), pairing random forests with optimism-corrected bootstrapping is known to result in inflated estimates of model performance (Tantithamthavorn, McIntosh, Hassan, & Matsumoto, 2017), which is one of the problems ML is intended to protect against. Emerging evidence indicates that when random forests are paired with other resampling methods, such as cross-validation or traditional bootstrapping, the prediction of suicide attempts is nearly identical to that produced by logistic regression (Jacobucci, Littlefield, Millner, Kleiman, & Steinley, 2021; Littlefield et al., 2021). Collectively, findings from the suicide and eating-disorder fields call into question whether ML may be a panacea to improve treatment outcome prediction.

This study compared the accuracy of three types of predictive models (one traditional and two ML) with three types of resampling methods in the prediction of BED treatment outcomes using data from a randomized controlled trial (RCT; Grilo et al., 2020). The primary goal was to determine whether ML was superior to traditional models for predicting treatment outcomes. The secondary goal was to compare predictive accuracy across different ML models paired with different forms of resampling, to serve as an example for future researchers considering using ML. A final goal was to identify variables that most strongly predict BED treatment outcomes. We acknowledge that this last goal diverges from ML's primary purpose/promise, which is increasing predictive accuracy, not identifying single predictors (Kuhn & Johnson, 2013; Murdoch, Singh, Kumbier, Abbasi-Asl, & Yu, 2019). However, the identification of individual-level predictors may provide necessary information to enhance treatment prescription and refine therapeutic targets. Thus, this final aim represents a bridge between ML models’ aim of accurate prediction and the potentially useful convention of identifying individual variables that predict treatment outcomes.

## Method

### Participants

Participants were 191 patients (age 18–65 years) with BED and comorbid obesity [body mass index (BMI)⩾30] who participated in an RCT testing 6-month behavioral weight-loss (BWL) and stepped-care interventions (Grilo et al., 2020). A detailed description of the RCT is published (Grilo et al., 2020), thus only a brief description follows. Exclusionary criteria included: concurrent treatment for eating/weight, uncontrolled medical problems, severe psychiatric conditions (psychosis, bipolar disorder, current substance dependence), or current pregnancy/breastfeeding. The majority of participants were female (*n* = 136, 71.2%) and identified as White (*n* = 150, 78.5); mean age was 48.4 years (s.d. = 9.5) and mean BMI was 39.0 (s.d. = 6.0) kg/m^2^.

### Procedure

Participants were randomized to either BWL (*n* = 39) or stepped care (*n* = 152) delivered following manualized protocols (Grilo et al., 2020). Diagnostic and clinical interviews were performed and height/weight was measured at baseline and post-treatment, and a battery of psychometrically established measures was completed throughout treatment (months 1, 2, and 4) and at post-treatment (6 months). Post-treatment assessments were obtained for 89.5% of participants. BWL and stepped care treatments did not differ significantly in binge-eating remission (74.4% *v.* 66.5%) or binge-eating frequency (1.7 binges/month *v.* 2.7 binges/month) at post-treatment. Treatments also did not significantly differ on eating-disorder psychopathology or percent weight loss at post-treatment (5.1% *v.* 5.8%).

### Measures

#### Predictor variables (see online Supplementary Materials for detailed descriptions and rationale)

Predictor variables (Table 1) included demographics, baseline BMI and clinical characteristics, rapid response, and treatment condition (BWL *v.* stepped care).

**Table 1.** Baseline clinical characteristics, treatment conditions, and treatment outcomes (*N* = 191)

	*n* (%) or *M* (s.d.)
*Demographic characteristics*	
Sex	
Male	55 (28.80)
Female	136 (71.20)
Race	
Black or Asian	30 (15.71)
White	161 (84.29)
Ethnicity	
Not Hispanic or Latino	176 (92.15)
Hispanic or Latino	9 (4.71)
Not reported	6 (3.14)
Education	
Less than Bachelor's degree	90 (47.12)
Bachelor's degree or more	98 (51.30)
Not reported	3 (1.57)
Age (years)	48.4 (9.5)
Body mass index	38.98 (5.98)
*Psychiatric comorbidity (Lifetime diagnoses on SCID-I/P)*	
Depressive disorders	99 (51.83)
Anxiety disorders	65 (34.03)
Posttraumatic stress disorder	15 (7.85)
Drug use disorder	31 (16.23)
Alcohol use disorder	43 (22.51)
*Eating-related psychopathology*	
Binge-eating frequency past month (EDE)	19.76 (14.63)
Overvaluation (EDE)	4.57 (1.04)
Dissatisfaction (EDE)	3.84 (1.74)
Restraint (EDE)	2.58 (1.91)
Restraint (TFEQ)	21.31 (13.54)
Behavioral indicator: Eat rapidly (QEWP)	140 (73.30)
Behavioral indicator: Eat until uncomfortably full (QEWP)	174 (91.58)
Behavioral indicator: Eat alone because embarrassed (QEWP)	126 (65.97)
Distress about binge eating (QEWP)	4.02 (0.98)
Weight cycling (QEWP)	2.94 (1.02)
Diet history (QEWP)	3.37 (1.41)
Emotional overeating (EOQ)	1.81 (1.23)
Food thought suppression (FTSI)	33.32 (12.15)
Other psychological symptoms and features	
*Food addiction ‘category’ (YFAS)*	117 (61.26)
Number of food addiction criteria met (YFAS)	4.77 (1.80)
Emotion regulation: Nonacceptance (DERS)	11.21 (4.85)
Emotion regulation: Difficulties with goals (DERS)	12.75 (5.26)
Emotion regulation: Impulse control difficulties (DERS)	12.37 (5.35)
Emotion regulation: Lack of awareness (DERS)	16.73 (5.54)
Emotion regulation: Limited access to strategies (DERS)	15.55 (6.65)
Emotion regulation: Lack of clarity (DERS)	10.32 (3.70)
Self-control (BSCS)	3.01 (0.63)
Weight bias internalization (WBIS)	4.59 (1.25)
Depression score (BDI)	15.11 (8.74)
Self-esteem (RSES)	20.03 (6.53)
Interpersonal problems (IIP)	1.05 (0.65)
Cognitive rumination: Brooding (RRS)	2.17 (0.77)
Cognitive rumination: Reflecting (RRS)	1.88 (0.66)
Mental health composite (SF36)	42.70 (11.12)
Physical health composite (SF36)	44.03 (10.29)
*Treatment condition*	
Standard care	39 (20.42)
Stepped care	152 (79.58)
Rapid response (⩾65% reduction binge-eating by week 4)	120 (62.83)
*Treatment outcomes*	
Binge-eating abstinence	106 (55.50)
Binge-eating reduction (%)	84.47 (37.63)
Eating-disorder psychopathology (EDE Global)	1.77 (0.84)
Weight loss ⩾5%	82 (42.93)
Weight loss (%)	4.21 (7.21)

SCID-I/P, Structured Clinical Interview for DSM Diagnosis; EDE, Eating Disorder Examination; TFEQ, Three Factor Eating Questionnaire; QEWP, Questionnaire on Eating and Weight Pattern; EOQ, Emotional Overeating Questionnaire, FTSI, Food Thought Suppression Inventory, YFAS, Yale Food Addiction Scale, DERS, Difficulties in Emotion Regulation Scale, BSCS, Self-Control Scale, WBIS, Weight Bias Internalization Scale, BDI, Beck Depression Inventory, RSES, Rosenberg Self-Esteem Scale, IIP, Inventory of Interpersonal Problems, RRS, Rumination Scale.

*Note*: Binge-eating percent reduction was log-transformed for analyses, though raw values are presented here for ease of interpretation.

**Psychiatric comorbidities**. *Structured Clinical Interview for DSM Axis I Psychiatric Disorders* (SCID-I/P; First, Spitzer, Gibbon, & Williams, 1997) assessed lifetime *DSM-IV* (APA, 1994) psychiatric disorders. Disorder classes considered in analyses were depressive, anxiety, posttraumatic stress, and drug and alcohol use disorders.

**Eating-related psychopathology**. *Eating Disorder Examination* interview (EDE; Fairburn, Cooper, & O'Connor, 2008), Three-Factor Eating Questionnaire (TFEQ; Anglé et al., 2009), Questionnaire on Eating/Weight Patterns–Revised (QEWP-R; Spitzer, Yanovski, & Marcus, 1994), Emotional Overeating Questionnaire (Masheb & Grilo, 2006), and Food Thought Suppression Inventory (Barnes, Fisak, & Tantleff-Dunn, 2010) assessed multiple domains of eating-related psychopathology including: binge-eating frequency (EDE), weight/shape overvaluation (EDE), weight/shape dissatisfaction (EDE), restraint (EDE, TFEQ), behavioral indicators for loss-of-control eating for *DSM-IV* BED diagnosis (QEWP-R), distress about binge eating (QEWP-R), weight cycling (QEWP-R), diet history (QEWP-R), emotional overeating (EOQ), and food thought suppression (FTSI).

**Other psychological symptoms/features**. Psychological symptoms/features relevant to BED (theoretically/empirically) listed below were included as predictors.

***Food addiction***. Number of food addiction criteria met and food addiction categorization (present *v.* absent) were assessed using the *Yale Food Addiction Scale* (Gearhardt, Corbin, & Brownell, 2009).

***Emotion regulation difficulties.*** Emotion regulation was assessed using the *Difficulties in Emotion Regulation Scale* (Gratz & Roemer, 2004). This 36-item self-report scale includes six subscales (nonacceptance, difficulties meeting goals, impulse control problems, low awareness, limited strategies, and low clarity), which were included as separate predictors.

***Self-control.*** Perceived self-control was assessed with the 13-item self-report *Self-Control Scale–Brief* (Tagney, Baumeister, & Boone, 2004).

***Weight bias internalization.*** Weight bias internalization, or the degree to which individuals have internalized negative beliefs about overweight or obesity, was assessed with the 11-item self-report *Weight Bias Internalization Scale* (Durso & Latner, 2008).

***Depression scores.*** Depressive symptoms experienced in the past week were assessed with the 21-item self-report *Beck Depression Inventory* (Beck & Steer, 1987).

***Self-esteem.*** Self-esteem was assessed with the 10-item self-report *Rosenberg Self Esteem Scale* (RSES; Rosenberg, 1989).

***Interpersonal problems.*** The extent to which people experience difficulties in their interpersonal functioning was assessed with the 32-item self-report *Inventory of Interpersonal Problems* (Barkham, Hardy, & Startup, 1996).

***Cognitive rumination.*** Two types of cognitive rumination, reflecting and brooding, were assessed with the 10-item self-report *Ruminative Responses Scale* (Treynor, Gonzalez, & Nolen-Hoeksema, 2003).

***Physical and mental health.*** The 36-item self-report *Short Form Health Survey* (Ware & Sherbourne, 1992) assessed physical and mental functioning and quality of life.

***Treatment variables.*** Two treatment-related variables were included as predictors: treatment condition (BWL or stepped care) and exhibiting rapid response (⩾65% reduction in binge-eating frequency at the month 1 assessment).

#### Outcome variables

Outcome variables reflected both eating-disorder psychopathology and weight loss, and included complementary approaches of analyzing variables in categorical and continuous formats.

**Binge-eating abstinence and binge-eating reduction**. Binge-eating abstinence was defined as having zero binge-eating episodes during final month of treatment (EDE). Percent reduction in binge-eating episodes from pre- to post-treatment was also calculated (EDE).

**Eating-disorder psychopathology.** Eating-disorder psychopathology was measured using the EDE Global score (Fairburn et al., 2008).

**Percent weight loss and weight loss ⩾5%.** Percent weight loss was calculated from subtracting posttreatment weight from pretreatment weight, dividing by pretreatment weight, and multiplying by 100. A dichotomous variable was also created based on whether weight loss was ⩾5%. Losing five percent of body weight is associated with physiological benefits (Magkos et al., 2016) and is frequently used in BED and obesity treatment studies.

### Data analytic plan

Analyses were completed using R computing software (R Core Team, 2020), using the following packages: mice (van Buuren & Groothuis-Oudshoorn, 2011), caret (Kuhn, 2008), glmnet (Friedman, Hastie, & Tibshirani, 2010), and random Forest (Liaw & Wiener, 2002). dplyr (Wickham, François, Henry, & Müller, 2021) was used to clean data and ggplot2 (Wickham, 2016) was used to create figures.

#### Missing data

We ran analyses with both the overall sample and the subsample who completed the post-treatment assessment (*n* = 171; see online Supplementary Table S1 for comparison of those who did *v.* did not complete the post-treatment assessment). The pattern of results was highly similar and we present analyses for the full intent-to-treat sample (*N* = 191). The proportion of missing data was 2.1%. The maximum proportion of missing data was 4% for any single predictor and 15% for any single outcome. After completing diagnostics to identify variables related to missingness, data were judged to be missing at random. Missing data for baseline characteristics were imputed with multivariate imputations with chained equations. Missing data for categorical outcomes were failure imputed (e.g. if data were missing to determine binge-eating abstinence, non-abstinence was coded). Missing data for continuous outcomes were replaced with estimated marginal means for each treatment group, obtained through multilevel modeling (Grilo et al., 2020).

#### Models

Three types of models were used to predict treatment outcomes: traditional logistic/linear regression^†^^1^, elastic net regression, and random forests. One benefit of logistic/linear regression is high interpretability. Weaknesses include potential to overfit and traditionally limited predictive power (King & Resick, 2014). Random forests, in contrast, have higher predictive performance but are less interpretable. Elastic nets have intermediate predictive performance and interpretability. Thus, the combination of these three types of models allows for comprehensive comparison among models across a spectrum of interpretability and prediction. We describe the models briefly below. In addition, the online Supplementary Materials provide further details, and we recommend reviewing Kuhn and Johnson (2013) for comprehensive descriptions.

Elastic net is a linear regression method that contains two regularization parameters, lambda and alpha, which are tuned to achieve the best model prediction. Random forests are a non-linear ensemble method comprised of hundreds of individual trees. Each tree in the forest is estimated from a random subset of predictors, and within each tree, the data are recursively partitioned to find the specific values of the predictors that divide the data into subgroups with the smallest sums of squares error values. This process of creating subgroups within subgroups is repeated until further splits do not result in improved model fit. Results are aggregated across trees to result in an overall metric of predictive performance.

After identifying the optimal models, three types of resampling were completed and compared: repeated 10-fold cross-validation, traditional bootstrapping, and optimism-corrected bootstrapping. Resampling is an umbrella term for methods to prevent overfitting a model to data. Repeated 10-fold cross-validation and traditional bootstrapping were used per recommendations (Kuhn & Johnson, 2013; Tantithamthavorn et al., 2017). Optimism-corrected bootstrapping was used given its use in initial ML in clinical psychological science (e.g. Fox et al., 2019; Huang et al., 2020; Walsh et al., 2017).

Repeated 10-fold cross-validation splits the dataset into 10 equal-sized folds. Nine folds are used to train the model on the data and one fold is used to test the model and evaluate its performance. This process is repeated 10 times, with a separate fold held out as the test set each time. Across these 10 repetitions, results are averaged to indicate overall model performance. Bootstrap resampling means that a bootstrap sample is drawn repeatedly (*n* = 100) from an overall sample. Optimism-corrected bootstrap resampling is similar to bootstrap resampling but in addition to the model being estimated from the bootstrap samples (*n* = 100), the model is also estimated on the original dataset. The difference between the model's performance in the bootstrap samples and on the original dataset produces a metric called optimism, which quantifies the level of overfitting of the model to the data. The optimism value is then subtracted from the overall metric of model performance. Optimism-corrected bootstrapping should theoretically produce more stringent results. However, optimism-corrected bootstrapping results in highly inflated results of model performance when paired with random forests (Jacobucci et al., 2021; Tantithamthavorn et al., 2017). Thus, we include this resampling method to demonstrate the differences that can arise from various combinations of ML models and resampling methods.

For each model, the following pre-processing of predictors was performed: identification and removal of predictors with near-zero variance, identifying whether any variables may be assessing similar underlying constructs, transformations for non-normal distributions, and centering and scaling. Ethnicity and two BED behavioral indicators (‘eating large amounts of food when not physically hungry’ and ‘feeling guilty, depressed, or disgusted with oneself after an eating binge’) had little variance and were removed from models. Because race and education had little variance [e.g. the only races reported in addition to White were Asian (*n* = 2) and Black (*n* = 28)], these variables were dichotomized. The largest correlation among predictors was *r* = .80 (for self-esteem and depression scores), suggesting that no predictors were too highly correlated and all predictors were entered into the models (*r* cutoff = 0.90). The binge-eating frequency at baseline and binge-eating reduction were log-transformed prior to imputation.

Across all model types (i.e. logistic/linear regression, elastic net, and random forest), performance for categorical outcomes was determined based on the area under the receiver operator characteristic curve (AUC) value. AUC of 0.50 indicates chance-level prediction. AUC classifications are categorized as follows: ⩽0.59 = extremely poor, 0.60–0.69 = poor, 0.70–0.79 = fair, 0.80–0.89 = good, and ⩾0.90 = excellent. Performance for continuous outcomes was determined based on root mean square error (RMSE) values and *R*^2^. RMSE values are in the same units as the outcome variable and indicate the average difference between the observed and predicted values. Lower RMSE indicates greater accuracy. *R*^2^ indicates the proportion of outcome variance explained by the model. Confidence intervals are presented for AUCs, RMSE, and *R*^2^ to facilitate comparison across models and resampling methods. For each outcome, the one standard error rule was used to select the optimal model (i.e. the model that is most parsimonious and whose error is no more than one standard error of the best-fitting model).

#### Predictor importance

ML analyses are computationally heavy and certain models have limited interpretability and vague clinical implications. To increase the clinical utility of results, we identified the most important predictors for each optimal model and for each resampling method using the caret package. For logistic, linear, and elastic net regressions, variable importance was calculated from the absolute values of each parameter's *t* test statistic, such that higher values indicate more important variables. For random forests, variable importance was calculated based on how much model fit changed if a predictor's input were permuted over all trees. Results across resampling methods were similar and we averaged predictor importance across resampling methods for each model type. Variable importance calculations do not identify the directionality of associations; thus, regression coefficients for logistic, linear, and elastic net regressions are shown in online Supplementary Tables S2–S6 (directionality is not modeled with random forests).

## Results

Table 1 summarizes demographic, baseline clinical characteristics, and treatment outcomes. Table 2 shows AUC values for categorical outcomes and RMSE and *R*^2^ values for continuous outcomes.

**Table 2.** Model performance for categorical outcomes as indicated by area under the receiver operator characteristic curve values, and continuous outcomes as indicated by root mean square error and *R*^2^ values

	AUC (95% CI)		
	Logistic	Elastic net	Random forest
Binge-eating abstinence			
10 repeated CV	0.50 (0.48–0.52)	0.51 (0.48–0.53)	0.49 (0.47–0.52)
Bootstrap	0.50 (0.49–0.52)	0.53 (0.52–0.54)	0.50 (0.49–0.51)
Optimism bootstrap	0.60 (0.59–0.61)	0.59 (0.58–0.60)	0.93 (0.92–0.94)
Weight loss ⩾5%			
10 repeated CV	0.66 (0.64–0.69)	0.68 (0.66–0.70)	0.59 (0.56–0.61)
Bootstrap	0.61 (0.59–0.62)	0.63 (0.62–0.65)	0.56 (0.55–0.57)
Optimism bootstrap	0.71 (0.70–0.73)	0.73 (0.72–0.74)	0.94 (0.93–0.95)

	RMSE (95% CI)			*R*^2^ (95% CI)		
	Linear	Elastic net	Random forest	Linear	Elastic net	Random forest
Binge-eating reduction % (*log*)						
10 repeated CV	0.42 (0.36–0.48)	0.29 (0.22–0.36)	0.28 (0.21–0.35)	0.04 (0.03–0.05)	0.04 (0.03–0.05)	0.05 (0.04–0.06)
Bootstrap	0.53 (0.50–0.57)	0.35 (0.30–0.40)	0.35 (0.30–0.40)	0.012 (0.008–0.015)	0.012 (0.008–0.015)	0.011 (0.009–0.013)
Optimism bootstrap	0.51 (0.48–0.55)	0.48 (0.44–0.52)	0.37 (0.32–0.42)	0.009 (0.007–0.013)	0.049 (0.048–0.052)	0.634 (0.632–0.636)
Eating-disorder psychopathology						
10 repeated CV	0.81 (0.79–0.84)	0.73 (0.71–0.75)	0.73 (0.71–0.76)	0.18 (0.15–0.21)	0.27 (0.24–0.30)	0.25 (0.22–0.28)
Bootstrap	0.91 (0.90–0.93)	0.74 (0.73–0.75)	0.75 (0.74–0.76)	0.12 (0.10–0.13)	0.23 (0.21–0.24)	0.21 (0.20–0.23)
Optimism bootstrap	0.81 (0.79–0.82)	0.74 (0.73–0.75)	0.61 (0.60–0.62)	0.17 (0.16–0.19)	0.23 (0.22–0.24)	0.63 (0.62–0.64)
Weight loss %						
10 repeated CV	7.39 (7.18–7.59)	7.19 (6.97–7.41)	7.07 (6.90–7.24)	0.12 (0.10–0.14)	0.12 (0.10–0.15)	0.05 (0.04–0.07)
Bootstrap	8.29 (8.15–8.43)	7.61 (7.48–7.74)	7.30 (7.19–7.42)	0.07 (0.06–0.08)	0.056 (0.047–0.065)	0.021 (0.017–0.026)
Optimism bootstrap	7.34 (7.20–7.48)	7.08 (6.95–7.21)	5.88 (5.78–6.02)	0.10 (0.09–0.11)	0.10 (0.09–0.11)	0.592 (0.586–0.594)

AUC. area under the receiver operator characteristic curve; RMSE, root mean square error, 10 repeated CV, repeated 10-fold cross-validation.

*Note*: Higher AUC values indicate greater predictive accuracy; lower RMSE values and higher *R^2^* values indicate greater predictive accuracy.

Across resampling methods, logistic regressions had extremely poor performance for prediction of binge-eating abstinence and poor to fair prediction of ⩾5% weight loss. Relative to logistic regressions and across resampling methods, elastic nets had similarly poor prediction of binge-eating abstinence and >=5% weight loss. Random forests with repeated 10-fold cross-validation and bootstrapping had similar AUCs as logistic regression with the same resampling methods in the prediction of binge-eating abstinence but lower AUCs than logistic regression in the prediction of ⩾5% weight loss. Random forests with optimism-corrected bootstrapping had excellent predictive performance.

Across resampling methods, for the prediction of binge-eating reduction, eating-disorder psychopathology, and weight loss, overall, RMSE values were significantly lower for elastic net and random forest than for linear regression (though exceptions were (1) elastic net with optimism corrected bootstrapping in predicting binge-eating reduction and (2) elastic net and random forest with 10-fold cross-validation in predicting weight loss). For *R*^2^ values, elastic nets and random forests with 10-fold cross-validation and bootstrapping had similar *R*^2^ as linear regression in predicting eating-disorder psychopathology but higher values in predicting binge-eating reduction. *R*^2^ for random forests with optimism-corrected bootstrapping across outcomes were significantly higher than all other models and resampling methods.

**Fig. 1.**
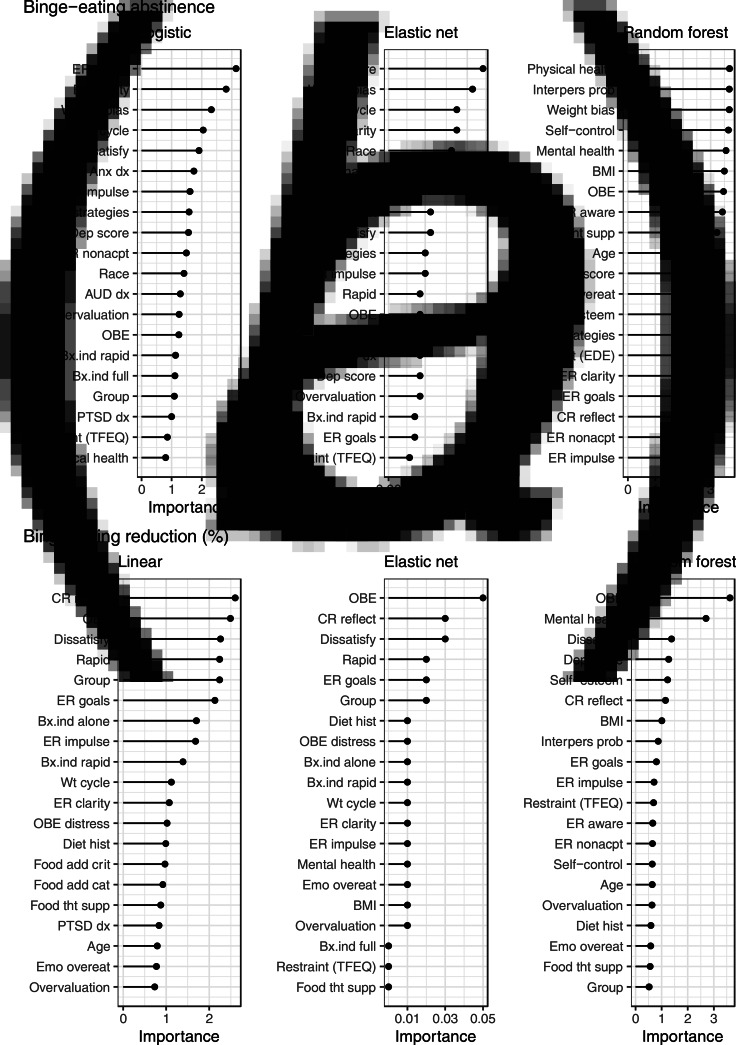
Top 20 average variable importance scores across resampling methods for each model type in the prediction of (*a*) binge-eating abstinence and (*b*) binge-eating reduction (%). *Note.* ER = emotion regulation, Weight bias = weight bias internalization, Wt cycle = weight cycling, Dissatisfy = weight/shape dissatisfaction, Anx dx = anxiety disorder, Dep score = depression score, nonacpt = nonacceptance, AUD dx = alcohol use disorder, overvaluation = weight/shape overvaluation, OBE = objective binge episode, Bx.ind = binge-eating disorder behavioral indicator, TFEQ = Three Factor Eating Questionnaire, rapid = rapid treatment response, Interpers prob = interpersonal problems, BMI = body mass index, Food tht supp = Food thought suppression, Emo overeat = emotional overeating, CR = cognitive rumination, Diet hist = diet history, Food add crit = food addiction criteria, Food add cat = food addiction category. Each *x* axis has a unique scale. Despite differing scales, interpretation remains consistent where higher variable importance corresponds to greater importance in predictive accuracy.

**Fig. 2.**
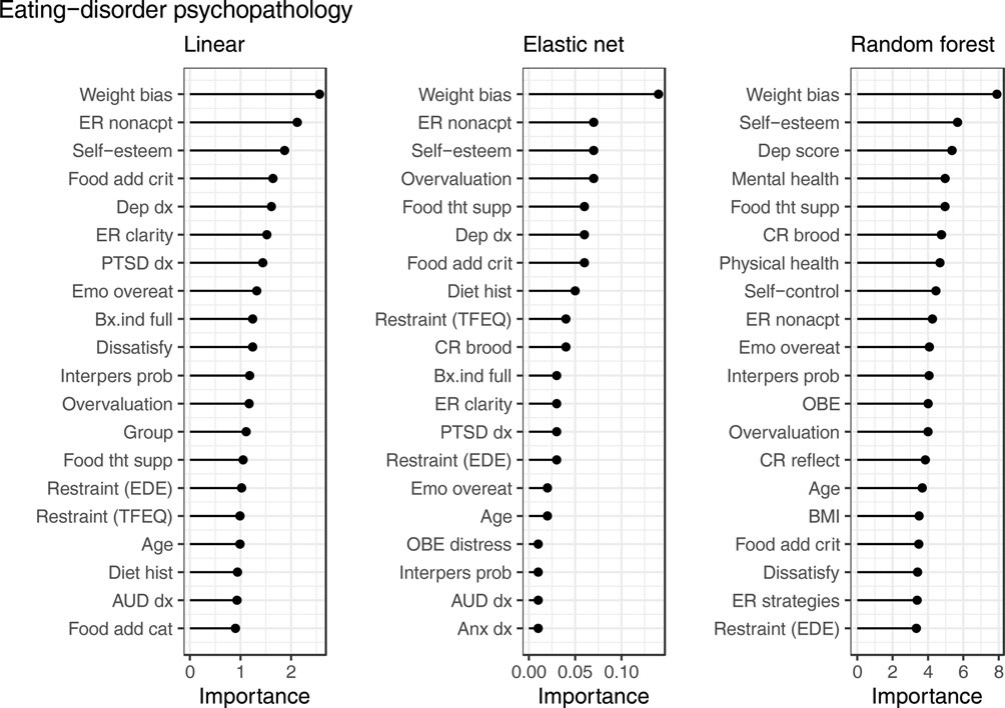
Top 20 average variable importance across resampling methods for each model type in the prediction of eating-disorder psychopathology. *Note.* Weight bias = weight bias internalization, ER = emotion regulation, Food add crit = food addiction criteria, Dep dx = depressive disorder, Emo overeat = emotional overeating, Bx.ind = binge-eating disorder behavioral indicator, Dissatisfy = weight/shape dissatisfaction, Interpers prob = interpersonal problems, overvaluation = weight/shape overvaluation, Food tht supp = food thought suppression, EDE = Eating Disorder Examination, TFEQ = Three Factor Eating Questionnaire, Diet hist = diet history, AUD dx = alcohol use disorder, Food add cat = food addiction category, CR = cognitive rumination, Anx dx = anxiety disorder, Dep score = depression score, OBE = objective binge episode. Each *x* axis has a unique scale. Despite differing scales, interpretation remains consistent where higher variable importance corresponds to greater importance in predictive accuracy.

**Fig. 3.**
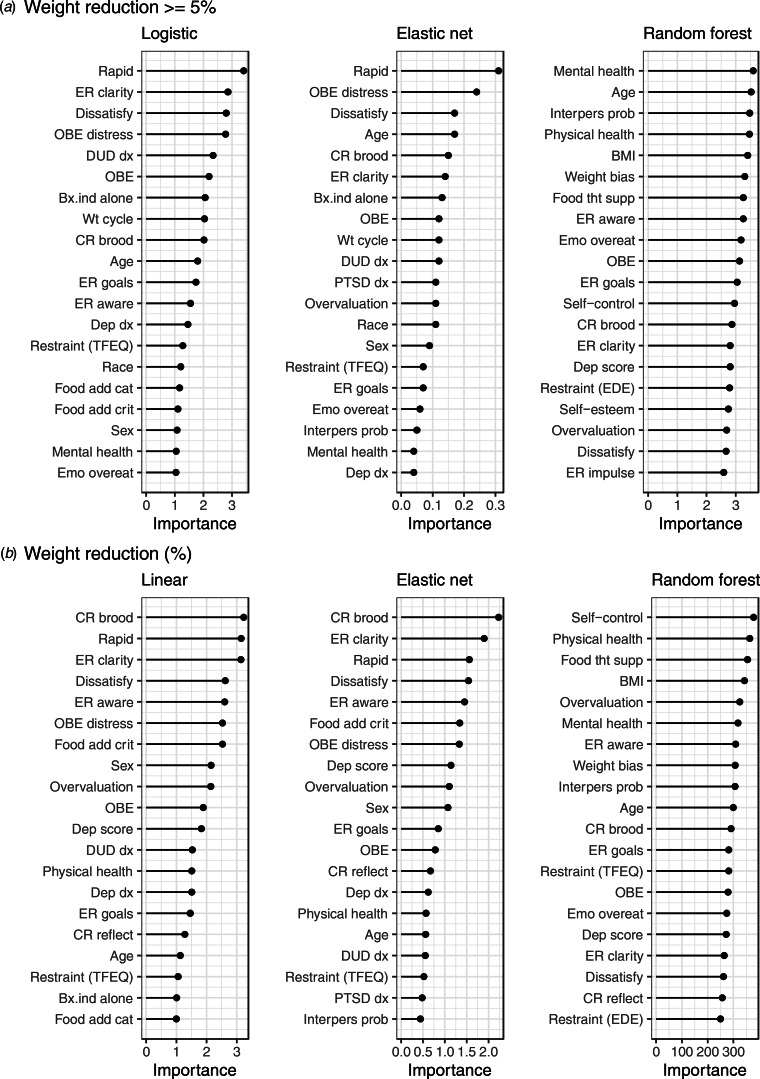
Top 20 average variable importance across resampling methods for each model type in the prediction of (*a*) weight reduction ⩾5% and (*b*) weight reduction (%). *Note*. Rapid = rapid treatment response, ER = emotion regulation, Dissatisfy = weight/shape dissatisfaction, OBE = objective binge episode, DUD dx = drug use disorder diagnosis, Bx.ind = binge-eating disorder behavioral indicator, Wt cycle = weight cycling, CR = cognitive rumination, Dep dx = depressive disorder, TFEQ = Three Factor Eating Questionnaire, Food add cat = food addiction category, Food add crit = Food addiction criteria, Emo overeat = emotional overeating, Overvaluation = weight/shape overvaluation, Interpers prob = interpersonal problems, BMI = body mass index, Food tht supp = Food thought suppression, Dep score = depression score,. Each *x* axis has a unique scale. Despite differing scales, interpretation remains consistent where higher variable importance corresponds to greater importance in predictive accuracy.

The 20 predictors with the highest average importance across resampling methods are shown in Figs 1–3. The strongest predictors of binge-eating abstinence (Fig. 1) were: low weight bias internalization (logistic, elastic net, and random forest), low lack of awareness of emotions (logistic and elastic net), physical health composite (random forest), and interpersonal problems (random forest). The strongest predictors of binge-eating reduction (Fig. 1) were: higher binge-eating baseline frequency (logistic, elastic net, and random forest), higher weight/shape dissatisfaction (logistic, elastic net, and random forest), lower reflecting cognitive rumination (linear and elastic net), and mental health composite (random forest).

The strongest predictors of eating-disorder psychopathology (Fig. 2) were: higher weight bias internalization (linear, elastic net, and random forest), higher self-esteem (linear, elastic net, and random forest), and higher nonacceptance of emotions (linear and elastic net).

The strongest predictors of ⩾5% weight loss (Fig. 3) were a rapid response to treatment (linear and elastic net) and mental health composite (random forest). The strongest predictors of weight loss (Fig. 3) were: lower brooding cognitive rumination (linear and elastic net), rapid treatment response (linear and elastic net), higher emotional clarity (linear and elastic net), self-control (random forest), and physical health composite (random forest).

## Discussion

This study examined how accurately combinations of traditional *v.* ML models and resampling methods predicted BED treatment outcomes. ML models showed little advantage over traditional models in predictive accuracy across BED outcomes (binge-eating, eating-disorder psychopathology, and weight loss). Although the different analytic models revealed some important predictors of key outcomes, their accuracy was modest. In cases where elastic net regressions and random forests showed greater predictive accuracy than traditional models, the overall prediction was still poor. ML using random forests with optimism-corrected bootstrapping yielded greater model prediction accuracy than all other models.

The superior and seemingly excellent prediction stemming from random forests with optimism-corrected bootstrapping, however, is likely inflated and may not reflect true model accuracy (Jacobucci et al., 2021; Tantithamthavorn et al., 2017). This inflation is a consequence of pairing random forests with optimism-corrected bootstrapping (Tantithamthavorn et al., 2017). We emphasize this to highlight a potential problem with the emerging ML literature in clinical psychology. Specifically, the initial ML applications predicting self-injurious behaviors, which suggested the high potential promise of ML for improving the prediction of relevant outcomes in clinical psychology, used random forests with optimism-corrected bootstrapping (Fox et al., 2019; Huang et al., 2020; Walsh et al., 2017). Thus, replication of those findings may prove difficult with unbiased resampling methods. Indeed, Jacobucci et al. (2021) found that random forests with non-inflated resampling methods (i.e. repeated 10-fold cross-validation and bootstrapping) in the prediction of suicide attempts yielded similar AUCs as traditional logistic regression.

While we recognize that our random forest with optimism-corrected bootstrapping results are inflated and did not plan on interpreting these results, we present them for two reasons. First, given the novelty of ML in clinical psychological/behavioral medicine, we wanted to provide an example of marked differences that emerge when different resampling methods are used with different ML models. Second, these findings echo Jacobucci et al. (2021) findings and recommendation that when using random forests, repeated 10-fold cross-validation or bootstrapping should be used as the resampling methods.

Our findings are consistent with emerging reports, within and outside of the eating disorders field, indicating that at least within the constraints of current psychological studies, non-inflated ML models perform comparably to traditional statistical methods (Buckman et al., in press; Espel-Huynh et al., 2021; Jacobucci et al., 2021; Littlefield et al., 2021; Zuromski et al., 2019). There are, however, some examples of ML outperforming traditional models (Haynos et al., in press; Kessler et al., 2015; Wang et al., 2021; for a review, see Chekroud et al. 2021). These examples offer points of consideration related to predictor selection and sample sizes that may be necessary for ML to achieve greater potential in clinical areas (Chekroud et al., 2021; Dwyer et al., 2018). Regarding predictors, although we included 42 predictors in analyses (i.e. including many more predictors than generally considered in traditional statistical approaches), we were limited to baseline RCT data. In contrast, for example, Kessler et al. (2015) used electronic health records to predict with high accuracy suicide deaths among psychiatrically hospitalized service members. Kessler et al. (2015) considered a total of 421 variables of multiple types (e.g. self-report, demographics, etc.) to include as potential predictors, and the final models included 73 predictors. Thus, increasing the number and/or variety of predictors may prove useful (Chekroud et al., 2021) to enhance accuracy. Regarding sample size, although *N* = 191 is the largest single-site RCT for BED, it is relatively small for ML algorithms. While small sample sizes can be partly overcome through methodological decisions (e.g. using repeated cross-validation), they can be problematic when they limit external validation. External validation is critical to assess the utility and generalizability of a specific ML algorithm. Thus, collecting larger samples or combining multiple samples to train, test, and validate models is a possible next step (Wang, 2021). Finally, ML may more accurately predict treatment outcomes with time-series predictors *v.* baseline data alone (e.g. Espel-Huynh et al., 2021; Wang, et al. (2021)). Overall, we believe that larger sample sizes, greater numbers of and variability in predictors, and repeated observations are important future directions in predicting eating-disorder treatment outcomes.

Our predictor importance analyses yielded evidence that adds to the limited eating disorder literature (Linardon et al., 2017); most clearly, findings provide further empirical confirmation for the positive prognostic significance of rapid response to treatment for BED (Grilo, White, Gueorguieva, Wilson, & Masheb, 2013; Grilo, White, Wilson, Gueorguieva, & Masheb, 2012b; Masheb & Grilo, 2007). Inspection of regression coefficients (online Supplementary Tables S5 and S6) indicates that patients with rapid response were more likely than those without rapid response to attain weight reduction ⩾5% and experience greater weight loss. These findings provide further confidence for using rapid response to treatment to inform stepped-care algorithms in BED treatment (Grilo et al., 2012b, 2020).

In addition, weight bias internalization was consistently among the strongest predictors of both binge-eating abstinence and eating-disorder psychopathology. Inspection of regression coefficients (online Supplementary Tables S2 and S4) indicates greater baseline weight bias internalization was prospectively associated with a lower likelihood of binge-eating abstinence and higher eating-disorder psychopathology at post-treatment. This is the first study to find that weight bias internalization may negatively impact BED treatment response; our findings (across multiple analyses) extend the cross-sectional associations between weight bias internalization with eating-disorder psychopathology in BED (Durso et al., 2012) and obesity (Pearl & Puhl, 2018). Pending external validation, our finding that greater weight bias internalization was associated with poorer eating-disorder outcomes following behaviorally based weight-loss treatments for BED could inform future treatment research testing the potential utility of incorporating cognitive interventions to address such internalized beliefs into behaviorally based interventions.

Strengths of this study include the rigorous assessment methods including the independent assessors administering investigator-based interviews and objective weight measurements. The analyses encapsulated nine models for each outcome to ensure that we identified any differences that occurred across various combinations of ML models resampling methods. We also highlight that while we considered 42 predictors given the goals of optimizing prediction and comparing models, we additionally performed logistic and linear regressions using only 10 predictors selected conceptually/empirically from the literature (plus to reduce type-I errors). The results of those reduced models yielded similar predictive performance to the models with all 42 predictors (see online Supplementary Table S7).

Several limitations are noteworthy. First, while we briefly interpret the variable importance results, we did this cautiously because predictive accuracy was roughly comparable across models (Fisher, Rudin, & Dominici, 2019). Second, even though some significant predictors emerged, their importance is relative and overall model predictions were limited. Third, the sample was primarily White, non-Hispanic, and well-educated and findings may not generalize to people with other characteristics. Fourth, while our predictor variables were quite broad and multimodal, they were not exhaustive. Finally, given the small sample size, we were unable to externally validate algorithms.

In summary, ML models with unbiased resampling methods provided a minimal advantage over traditional models in predictive accuracy for BED treatment outcomes. Improving prediction accuracy for eating disorder treatment outcomes remains a priority.

## Footnotes

† The notes appear after the main text.

## Note

1 Given that logistic and linear regressions are not well suited for analyzing large numbers of predictors, we also completed logistic and linear regressions using only 10 predictors (selected conceptually or empirically based on their associations with BED treatment response; see Grilo et al., 2012a; Linardon et al., 2017). These reduced models yielded similar predictive performance as the models with all 42 predictors. Given the consistency between the 10-predictor and 42-predictor traditional models, we report below the results of the 42-predictor models; findings from the 10-predictor traditional models are in the online Supplementary Material.

## Supplementary material

For supplementary material accompanying this paper visit https://doi.org/10.1017/S0033291721004748. click here to view supplementary material
