# Supplementary material for: Machine learning v. traditional regression models predicting treatment outcomes for binge-eating disorder from a randomized controlled trial
Source: Psychol Med. 2021 Nov 25;53(7):2777–88. doi: 10.1017/S0033291721004748 (PMC9130342; doi:10.1017/S0033291721004748)
Supplement: Supplementary file 1 [file S0033291721004748sup001.docx]

Machine learning versus traditional regression models predicting treatment outcomes for binge-eating disorder from a randomized controlled trial

**Supplemental Material**

**Measures**

A battery of established interviews and self-report questionnaires from the eating/weight literature were used to assess the predictor variables of eating disorder- and weight-related treatment outcomes. The measures were selected to assess a broad range of specific eating-disorder and eating behavior/weight-related constructs as well as associated psychological symptoms/features relevant (theoretically or empirically) to BED and—in some instances—because they were found to be associated with outcomes in previous studies. We emphasize that while we cite empirical support for the selected predictors, it is critical to keep in mind that—in most instances—the evidence is mixed for these and other potential variables, as no reliable predictors of outcomes (other than early rapid response to treatment) have been identified (Linardon et al., 2017; Vall & Wade, 2015). These measures, listed in the Methods section of the manuscript, are described below along with the rationale for their selection. We note that the number of predictor variables was intentionally large (relative to studies that use traditional statistical approaches) given that machine learning models are designed specifically to include/accommodate many predictors.

***Eating Disorder Examination***

Eating-disorder psychopathology was assessed with the Eating Disorder Examination (EDE; Fairburn, 2008). The EDE is a 28-item clinical interview that assesses both cognitive and behavioral eating disorder symptoms. The EDE is a widely-used measure that is traditionally scored with four subscales (restraint, eating concern, weight concern, and shape concern) in addition to a global score. While the global score indexes overall eating-disorder psychopathology, the subscales were intended to provide more fine-grained information on specific groupings of eating-disorder symptoms. EDE global and scale scores, which have been tested as predictors of outcome in several studies across eating disorders, have received mixed support overall (Vall & Wade, 2015) and in studies with BED (Grilo et al., 2021). However, the factor structure for the four subscales of the EDE has been consistently found to be a poor fit for data (e.g., Grilo et al., 2010). Thus, to understand how specific types of eating-disorder symptoms may predict outcomes, we used the modified three-factor, seven-item structure (Grilo et al., 2010) to assess weight and shape overvaluation ($\alpha$=.80), weight and shape dissatisfaction ($\alpha$=.90), and dietary restraint ($\alpha$=.67). We also included past-month binge-eating frequency at baseline as a predictor.

***Questionnaire on Eating and Weight Patterns***

The Questionnaire on Eating and Weight Patterns–Revised (QEWP; Spitzer, Yanovski, & Marcus, 1994) is a 27-item self-report measure used to screen for BED. Eight items from the QEWP were included as potential predictors: the five behavioral indicators for determining loss-of-control aspect of binge eating diagnosis (scored as present or absent), the level of distress about binge eating over the past six months (scored 1 [*not at all*] to 5 [*extremely*]), frequency of weight cycling (i.e., deliberately losing 20 pounds or more and gaining it back shortly thereafter; scored 1 [*never*] to 4 [*five times or more*]), and the proportion of time in adulthood spent dieting (scored 1 [*none or hardly any of the time*] to 5 [*nearly all of the time*]). Research has supported the validity of the five behavioral indicators (White & Grilo, 2011) and the distress criterion (Grilo & White, 2011) for BED.

***Emotional Overeating Questionnaire***

The Emotional Overeating Questionnaire (Masheb & Grilo, 2006) is a nine-item self-report measure that assesses how frequently in the last 28 days people overate in response to eight different emotional states (anxiety, sadness, loneliness, tiredness, anger, happiness, boredom, guilt) or in response to physical pain. Items are scored on a 0 (*0 days*) to 6 (*28 days*) scale. Items are averaged, where higher average scores indicate a higher frequency of overeating in response to emotions or pain ($\alpha$=.85). This was included as a predictor given empirical support for affect regulation models of binge eating, which propose that people engage in such overeating behaviors partly in response to emotional states (e.g., Grilo, Masheb, & Wilson, 2001; Smith et al., 2020).

***Food Thought Suppression Inventory***

The Food Thought Suppression Inventory (Barnes, Fisak, & Tantleff-Dunn, 2010) is an 11-item self-report questionnaire that assesses how frequently people attempt to avoid thinking about food. Items are scored on a 1 (*strongly disagree*) to 5 (*strongly agree*) scale. Items are summed, where higher scores indicate greater food thought suppression ($\alpha$=.92). Food thought suppression was included as a predictor given its positive association with eating-disorder psychopathology among patients with binge-eating disorder (Barnes, Masheb, White, & Grilo, 2013).

***Yale Food Addiction Scale***

Food addiction criteria and diagnosis were assessed using the Yale Food Addiction Scale (YFAS; Gearhardt, Corbin, & Brownell, 2009). The YFAS is a 25-item self-report measure that assesses seven criteria for addiction plus clinical impairment, all as they relate to food. Each YFAS criterion has its own scoring that results in a dichotomous score of being present or not. The criteria are summed. The presence of three or more criteria is categorized as food addiction being present. We included two variables related to food addiction as predictors: whether someone was categorized as having food addiction and the number of food addiction criteria that were present ($\alpha$=.67). While food addiction remains a controversial construct (Gearhardt & Hebebrand, 2021), we included these food addiction variables because this construct is relevant for potential mechanisms underlying binge eating (Schulte, Grilo, & Gearhardt, 2016) is and has been found to be associated with poor BED treatment outcomes (Romero et al., 2019).

***Difficulties in Emotion Regulation Scale***

Emotion regulation difficulties were assessed with the 36-item self-report Difficulties in Emotion Regulation Scale (Gratz & Roemer, 2004). Items assess how frequently people experience a particular aspect of emotion regulation on a 1 (*almost never*) to 5 (*almost always*) scale. Items are summed into six subscales: nonacceptance of emotions ($\alpha$=.92), difficulties meeting goals due to intense emotions ($\alpha$=.88), impulse control problems when experiencing intense emotions ($\alpha$=.90), low emotional awareness ($\alpha$=.85), limited emotion regulation strategies ($\alpha$=.84), and low emotional clarity ($\alpha$=.89). In general, emotion regulation difficulties were included as predictors because emotion dysregulation is implicated in several prominent theories of binge eating (Fairburn, Cooper, & Shafran, 2003). We included individual subscales as predictors given that emotion regulation is a multidimensional construct and recent emerging research using ecological momentary assessment has revealed complex associations between several aspects of emotion regulation, negative affect, and binge eating (Smith et al., 2020; Smith et al., 2021).

***Self-Control Scale–Brief***

Perceived self-control was assessed with the Self-Control Scale–Brief (Tagney, Baumesiter, & Boone, 2004). This is a 13-item self-report measure that assesses the degree of one’s perceived self-control in general. Participants indicate how much each statement reflects how they typically are, and items are scored on a 1 (*not at all*) to 5 (*very much*) Likert-type scale. Items are averaged ($\alpha$=.74), where higher scores indicate greater perceived self-control. Perceived self-control was included as a predictor due to its emergence as a predictor of binge-eating frequency in BED treatments (Anderson et al., 2020).

***Weight Bias Internalization Scale***

Weight bias internalization was assessed with the Weight Bias Internalization Scale (Durso & Latner, 2008). This is an 11-item self-report measure that assesses the degree to which individuals have internalized negative beliefs about overweight or obesity, such as perceiving oneself being less attractive because of one’s weight. Participants indicate how much they agree with each item on a 1 (*strongly disagree*) to 7 (*strongly agree*) scale. Items are averaged ($\alpha$=.85), where higher scores indicate greater weight bias internalization. Weight bias internalization was included as a predictor because it is consistently associated with greater eating disorder symptoms (e.g., Pearl & Puhl, 2018).

***Beck Depression Inventory***

Depression symptoms/levels were assessed with the Beck Depression Inventory (Beck & Steer, 1987). This is a 21-item self-report measure that assesses depressive symptoms experienced in the past week. Each item is scored on a 0–3 scale. Items are summed ($\alpha$=.88), where higher scores indicate greater symptom severity/level. Depression score was included as a predictor as it has received mixed support across treatment studies for eating disorders (Valle & Wade, 2015) including BED (Grilo, Masheb, & Crosby, 2012; Grilo et al., 2021).

***Rosenberg Self-Esteem Scale***

Self-esteem was assessed with the 11-item self-report Rosenberg Self-Esteem Scale (Rosenberg, 1989). Items present descriptions of views of oneself and are rated on a 1 (*strongly agree*) to 4 (*strongly disagree*). Some items are reverse scored and all items are summed ($\alpha$=.92), where higher scores indicate more negative views of oneself. Poor self-esteem is posited to be one factor that may contribute to eating disorder psychopathology (Fairburn, Cooper, & Shafran, 2003), has received mixed support as a predictor of outcomes across eating disorders (Vall & Wade, 2015) including BED (Masheb & Grilo, 2008).

***Inventory of Interpersonal Problems***

Interpersonal problems were assed with the Inventory of Interpersonal Problems (Barkham, Hardy, & Startup, 1996). This is a 32-item self-report measure assessing the extent to which people experience difficulties in their interpersonal relationships. Items are scored on a 0 (*not at all*) to 4 (*extremely*) scale and averaged, where higher scores indicate greater intensity of interpersonal problems ($\alpha$=.93). Interpersonal problems were included as a predictor because interpersonal functioning is central to the interpersonal model of the development and maintenance of binge eating (Ansell, Grilo, & White, 2012; Blomquist et al., 2012) and its effects have been found to be partly mediated by negative/depressive affect in BED (Ansell et al., 2012).

***Ruminative Responses Scale***

Two types of cognitive rumination, reflecting and brooding, were assessed with the Ruminative Responses Scale (Treynor, Gonzalez, & Nolen-Hoeksema, 2003). This is a 10-item self-report measure that assesses, in general, how often people think about their thoughts and feelings in response to experiencing stress or depression. The reflecting subscale includes five items that assess valence-neutral experiences, such as writing down what one is thinking to analyze it ($\alpha$=.72). The brooding subscale assesses negatively-valenced experiences, such as wondering what one is doing to deserve a negative experience ($\alpha$=.83). The items comprising each subscale are averaged, where higher scores indicate greater frequency of cognitive rumination. We included these types of cognitive rumination as predictors because cognitive rumination is a transdiagnostic risk factor for internalizing psychopathology (McLaughlin & Nolen-Hoeksema, 2011) that is positively associated with eating disorder symptoms among patients with BED and obesity (Wang, Lydecker, & Grilo, 2017). Moreover, rumination has been found to show a close interplay with negative affect in triggering binge eating (Smith et al., 2021).

***Short Form Health Survey***

Eating disorders impact both psychological and physical functioning. Thus, we included the Short Form Health Survey (Ware & Sherbourne, 1992), which is a 36-item self-report measure assessing multiple domains of physical and psychological functioning and quality of life. The physical health and mental health component scores ($\alpha$=.85) were included as predictors because both of these domains of health (Masheb & Grilo, 2004) and associated functional impairments (Udo & Grilo, 2018) are salient concerns for people with binge-eating disorder and obesity

**Missing Data**

We compared results with the subsample who completed the post-treatment assessment (*n*=171). The pattern of results was highly similar across analytic methods (see **Supplemental Table 1)**. Exhaustive exploratory analyses revealed that participants who completed vs. did not complete the post-treatment assessment differed on only 4 of the 42 demographic and clinical variables. Participants who did not complete the post-treatment assessment reported higher binge-eating distress, lower reflecting cognitive rumination, were less likely to exhibit rapid treatment response, and were less likely to have a Bachelor’s degree or more. Overall, there was strong evidence that the pattern of results presented below was not meaningfully impacted by whether participants completed the post-treatment assessment, and completion of the post-treatment assessment did not reliably differ by symptom severity. Accordingly, we thus present analyses using the full *N*=191.

**Machine Learning Models**

Elastic net is a linear regression method that can predict both categorical and continuous outcomes. It contains two regularization parameters, lambda and alpha, that are tuned to increase model prediction and achieve the best variance–bias tradeoff. The lambda penalty shrinks parameter estimates closer to zero in order to reduce variance, while introducing bias. The higher the lambda value, the more shrinkage will occur. The alpha penalty is a mixing parameter, where values range from 0–1. A value of 0 means that coefficients will be penalized, but not shrunk to exactly 0 and therefore all predictors will be retained in the model. An alpha value of 1 means that certain coefficients will be penalized to exactly 0 and therefore some predictors will be removed from the model. An alpha value between 0 or 1 indicates a mix between these two ends of the spectrum. Lambda and alpha values are tuned to achieve the best model prediction. In elastic nets, lambda was tuned across *n*=40 values ranging from .01–.20 and alpha was tuned across *n* = 7 values ranging from 0–1.

Random forests are a non-linear ensemble method that can predict both categorical and continuous outcomes. Random forests are comprised of individual trees, where the data are recursively partitioned to find the predictor and the predictor’s specific value that divides the data into two groups with the smallest sums of squares error values. This process of creating subgroups within subgroups is repeated until further splits do not result in improved model fit. Because individual decision trees can be biased and highly influenced by anomalies in the data, random forests estimate hundreds of individual decision tress (in this case, *n*=500). Each tree in the forest is estimated from a random subset of predictors, which means that the individual trees within the forest are diverse and are not highly correlated with one another. While it is common practice to set the number of predictors considered at each split (referred to as the *mtry* parameter) to the square root of the number of predictors, it has been argued that *mtry* should be tuned to identify the value that is most optimal (Kuhn & Johnson, 2013). Thus, in the current study with *n*=42 predictors, mtry was tuned across *n*=6 values ranging from 2 to 40. To identify overall predictive performance, the results of the forest of trees are aggregated.

**Resampling Methods**

Repeated 10-fold cross-validation splits the dataset into 10 equal-sized folds (e.g., when *N*=191 is divided into 10 folds, each fold’s *n*=19–20). Nine folds are used to train the model on the data and one fold is used to test the model and evaluate its performance. This process is repeated 10 times, with a separate fold held out as the test set each time. Across these 10 repetitions, results are averaged to indicate overall model performance. Repeated 10-fold cross-validation does not include replacement, meaning that each observation is represented across folds only once. For example, consider a dataset comprised of participants A–Z and 10-fold cross-validation were performed. If participant A was represented in Fold 1, participant A would not then be represented again in Folds 2–10.

Bootstrapping in a resampling context is similar to bootstrapping in other contexts, where a bootstrap sample is drawn repeatedly from an overall sample. Bootstrapping includes replacement, meaning that in the example above of participants A–Z, participant A may be included in the first bootstrap sample and may be selected again to be included in other bootstrap samples. Adding onto this example, participant D and participant N may not be included in any bootstrap samples. This means that the model will be trained on the bootstrap samples that include participants A–C, E–M, and O–Z and model performance will then be evaluated using the participants D and N (i.e., participants who were not included in the bootstrap samples).

**Traditional Models with Reduced Predictors**

In addition to running logistic and linear regressions with the full predictor set used in the machine learning models (*n* = 42 predictors), we also ran these traditional regression models with only 10 of the original predictors, given that the models are not well suited to large numbers of predictors. We selected the 10 predictors based on an overview of the conceptual plus empirical literature findings reviewed above and elsewhere (Vall & Wade, 2015; Linardon et al., 2017). These predictors were: sex, race, body mass index, binge-eating frequency, shape/weight overvaluation, Eating Disorder Examination Global score, any comorbid disorder, Beck Depression Inventory score, treatment group (stepped vs. standard care), and rapid response status (defined as reduction in eating binges ≥ 65% at treatment week 4). AUCs, RMSE, and *R*^2^ values are presented in **Supplemental Table 7**.

**Supplemental Material References**

Ansell, E. B., Grilo, C. M., & White, M. A. (2012). Examining the interpersonal model of binge eating and loss of control over eating in women. *International Journal of Eating Disorders, 45,* 43 – 50.

Barkham, M., Hardy, G. E., & Startup, M. (1996). The IIP-32: A short version of the Inventory of Interpersonal Problems. *British Journal of Clinical Psychology, 35,* 21–35.

Barnes, R. D., Fisak, B., & Tantleff-Dunn, S. (2010). Validation of the food thought suppression inventory. *Journal of Health Psychology, 15,* 373–381.

Barnes, R. D., Masheb, R. M., White, M. A., & Grilo, C. M. (2013). Examining the relationship between food thought suppression and binge eating disorder. *Comprehensive Psychiatry, 54,* 1077–1081.

Beck, A. T., & Steer, R. A. (1987). Manual for the Beck Depression Inventory. New York: Psychological Corporation.

Blomquist, K., Ansell, E. B., White, M. A., Masheb, R. M., & Grilo, C. M. (2012). Interpersonal problems and developmental trajectories of binge eating disorder. *Comprehensive Psychiatry, 53,* 1088 – 1095.

Durso, L. E., & Latner, J. D. (2008). Understanding self-directed stigma: Development of the weight bias internalization scale. *Obesity, 16 Suppl2,* S80–S86.

Fairburn, C. G., Cooper, Z., & O’Connor, M. (2008). The Eating Disorder Examination (16.0D). In C. G. Fairburn (Ed.), *Cognitive behavior therapy and eating disorders* (pp. 265–308). New York, NY: Guilford Press.

Fairburn, C. G., Cooper, Z., & Shafran, R. (2003). Cognitive behaviour therapy for eating disorders: a “transdiagnostic” theory and treatment. *Behaviour Research and Therapy, 41,* 509–528. DOI: 10.1016/S0005-7967(02)00088-8

Gearhardt, A. M., & Hebebrand, J. (2021). The concept of “food addiction” helps inform the understanding of overeating and obesity: Debate consensus. *American Journal of Clinical Nutrition, 113,* 274–276.

Gratz, K., & Roemer, L. (2004). Multidimensional assessment of emotion regulation and dysregulation: Development, factor structure, and initial validation of the Difficulties in Emotion Regulation Scale. *Journal of Psychopathology and Behavioral Assessment, 26,* 41–54.

Grilo, C. M., & White, M. A. (2011). A controlled evaluation of the distress criterion for binge eating disorder. *Journal of Consulting and Clinical Psychology, 79,* 509 – 514.

Grilo, C. M., Crosby, R. D., Peterson, C. B., Masheb, R. M., White, M. A., Crow, S. J., Wonderlich, S. A., & Mtichell, J. E. (2010). Factor structure of the eating disorder examination interview in patients with binge-eating disorder. *Obesity, 18,* 977–981.

Grilo, C. M., Gueorguieva, R., & Pittman, B. (2021). Examining depression scores as predictors and moderators of treatment outcomes in patients with binge-eating disorder. *International Journal of Eating Disorders, 54,* 1555 – 1559.

Grilo, C. M., Masheb, R. M., & Crosby, R. D. (2012). Predictors and moderators of response to cognitive behavioral therapy and medication for the treatment of binge-eating disorder. *Journal of Consulting and Clinical Psychology, 80,* 897–906.

Grilo, C. M., Masheb, R. M., & Wilson, G. T. (2001). Subtyping binge eating disorder. *Journal of Consulting and Clinical Psychology, 69,* 1066 – 1072.

Grilo, C. M., Thompson-Brenner, H., Shingleton, R. M., Thompson, D. R., & Franko, D. L. (2021). Clinical moderators and predictors of cognitive-behavioral therapy by guided-self-help versus therapist-led for binge-eating disorder: Analysis of aggregated clinical trials. *International Journal of Eating Disorders, 54,* 1875 – 1880.

Kuhn, M., & Johnson, K. (2013). *Applied predictive modeling.* New York: Springer.

Linardon, J., Garcia, X. D. L., & Brennan, L. (2017). Predictors, moderators, and mediators of treatment outcome following manualised cognitive-behavioural therapy for eating disorders: A systematic review. *European Eaitng Disorders Review, 25,* 3 – 12.

Masheb, R. M., & Grilo, C. M. (2004). Quality of life in patients with binge eating disorder. *Eating and Weight Disorders, 9,* 194 – 199.

Masheb, R. M., & Grilo, C. M. (2006). Emotional overeating and its association with eating disorder psychopathology among overweight patients with binge eating disorder. *International Journal of Eating Disorders, 39,* 141–146.

Masheb, R. M., & Grilo, C. M. (2008). Examination of predictors and moderators of self-help treatments of binge-eating disorder. *Journal of Consulting and Clinical Psychology, 76,* 900–904.

McLaughlin, K. A., & Nolen-Hoeksema, S. (2011). Rumination as a transdiagnostic factor in depression and anxiety. *Behaviour Research and Therapy, 49,* 186–193.

Pearl, R. L., & Puhl, R. M. (2018). Weight bias internalization and health: A systematic review. *Obesity Reviews, 19,* 1141–1163.

Romero, X., Agüera, Z., Granero, R., Sánchez, I., Riesco, N., Jiménez-Murcia, S., … & Fernández-Aranda, F. (2019). Is food addiction a predictor of treatment outcome among patients with eating disorder? *European Eating Disorders Review, 27,* 700–711.

Rosenberg, M. (1989). *Society and the adolescent self-image, revised edition.* Middletown, CT: Wesleyan University Press.

Schulte, E. M., Grilo, C. M., & Gearhardt, A. N. (2016). Shared and unique mechanisms underlying binge eating disorder and addictive disorders. *Clinical Psychology Review, 44,* 125 – 139.

Smith, K. E., Mason, T. B., Reilly, E. E., Hazzard, V. M., Borg, S. L., Dvorak, R., Crosby, R. D., & Wonderlich, S. A. (2021). Examining prospective mediational relationships between momentary rumination, negative affect, and binge eating using ecological momentary assessment. *Journal of Affective Disorders Reports, 5,* 100138.

Smith, K. E., Mason, T. B., Schaefer, L. M., Anderson, L. M., Hazzard, V. M., Crosby, R. D., … & Peterson, C. B. (2020). Micro-level de-coupling of negative affect and binge eating in relationship to macro-level outcomes in binge eating disorder treatment. *Psychological Medicine, 29,* 1–9.

Spitzer, R. L., Yanovski, S. Z., & Marcus, M. D. (1994). *Questionnaire on Eating and Weight Patterns, Revised.* Pittsburgh, PA: Behavioral Measurement Database Services (Producer).

Tagney, J. P., Baumeister, R. F., & Boone, A. L. (2004). High self-control predicts good adjustment, less pathology, better grades, and interpersonal success. *Journal of Personality, 72,* 271–324.

Treynor, W., Gonzalez, R., & Nolen-Hoeksema, S. (2003). Rumination reconsidered: A psychometric analysis. *Cognitive Therapy and Research, 27,* 247–259.

Udo, T., & Grilo, C. M. (2018). Prevalence and correlates of DSM-5 defined eating disorders in a nationally representative sample of U.S. adults. *Biological Psychiatry, 84,* 345 – 354.

Vall, E., & Wade, T. D. (2015). Predictors of treatment outcome in individuals with eating disorders: A systematic review and meta-analysis. *International Journal of Eating Disorders, 48,* 946 – 971.

Wang, S. B., Lydecker, J. A., & Grilo, C. M. (2017). Rumination in patients with binge-eating disorder and obesity: Associations with eating disorder psychopathology and weight-bias internalization. *European Eating Disorders Review, 25,* 98–103.

Ware, J. E., & Sherbourne, C. D. (1992). The MOS 36-item Short-Form Health Survey (SF-36): I. Conceptual framework and item selection. *Medical Care, 30,* 473–483.

White, M. A., & Grilo, C. M. (2011). Diagnostic efficiency of DSM-IV indicators for binge eating episodes. *Journal of Consulting and Clinical Psychology, 79,* 75 – 83.

**Supplemental Table 1.** *Comparison of predictor variables between people who completed the post-treatment assessment vs. did not complete the post-treatment assessment*

|  | Completed post-treatment assessment  *n* = 171 | No post-treatment assessment  *n* = 20 |  | |  |
| --- | --- | --- | --- | --- | --- |
|  | *n* (%) or  *M* (*SD*) | *n* (%) or  *M* (*SD*) | $\chi$^2^ or *t* | | *p* |
| Demographic characteristics |  |  |  | |  |
| Gender |  |  | 0.42 | | .62 |
| Man | 48 (28.07) | 7 (35.00) |  | |  |
| Woman | 123 (71.93) | 13 (65.00) |  | |  |
| Race |  |  | 0.55 | | .55 |
| Black or Asian | 28 (16.37) | 2 (10.00) |  | |  |
| White | 143 (83.63) | 18 (90.00) |  | |  |
| Ethnicity |  |  | 0.73 | | .87 |
| Not Hispanic or Latino | 157 (91.81) | 19 (95.00) |  | |  |
| Hispanic or Latino | 8 (4.68) | 1 (5.00) |  | |  |
| Not reported | 6 (3.51) | 0 (0) |  | |  |
| **Education** |  |  | **4.69** | | **.04** |
| Less than Bachelor’s degree | 76 (44.44) | 14 (70.00) |  | |  |
| Bachelor’s degree or more | 95 (55.56) | 6 (30.00) |  | |  |
| Age | 48.11 (9.33) | 50.15 (10.34) | 0.84 | | .41 |
| Body mass index | 38.82 (5.75) | 40.40 (5.68) | 0.89 | | .38 |
| Lifetime psychiatric comorbidities |  |  |  | |  |
| Depressive disorder | 85 (49.71) | 14 (70.00) | 2.95 | | .10 |
| Anxiety disorder | 57 (33.33) | 8 (40.00) | 0.35 | | .63 |
| Posttraumatic stress disorder | 12 (7.02) | 3 (15.00) | 1.58 | | .36 |
| Drug use disorder | 29 (16.96) | 2 (10.00) | 0.64 | | .55 |
| Alcohol use disorder | 39 (22.81) | 4 (20.00) | 0.08 | | .80 |
| Eating-related psychopathology |  |  | |  |  |
| Binge-eating frequency past month (EDE) | 19.67 (14.81) | 20.55 (13.38) | | 0.27 | .79 |
| Overvaluation (EDE) | 4.52 (1.04) | 4.97 (0.99) | | 1.93 | .07 |
| Dissatisfaction (EDE) | 3.82 (1.72) | 4.00 (1.95) | | 0.40 | .69 |
| Restraint (EDE) | 2.54 (1.88) | 2.88 (2.17) | | 0.68 | .51 |
| Restraint (TFEQ) | 21.92 (13.12) | 16.46 (16.14) | | -1.46 | .16 |
| Behavioral indicator: Eat rapidly (QEWP) | 123 (75.00) | 14 (73.68) | | 0.02 | 1.00 |
| Behavioral indicator: Eat until uncomfortably full  (QEWP) | 151 (90.96) | 18 (90.00) | | 0.02 | 1.00 |
| Behavioral indicator: Eat alone because embarrassed  (QEWP) | 107 (64.46) | 15 (75.00) | | 0.88 | .45 |
| **Distress about binge eating (QEWP)** | **3.95 (0.98)** | **4.55 (0.76)** | | **3.21** | **.003** |
| Weight cycling (QEWP) | 2.89 (1.03) | 3.30 (0.92) | | 1.84 | .07 |
| Diet history (QEWP) | 3.31 (1.42) | 3.70 (1.34) | | 1.21 | .24 |
| Emotional overeating (EOQ) | 1.74 (1.18) | 2.26 (1.33) | | 1.68 | .11 |
| Food thought suppression (FTSI) | 33.31 (11.91) | 32.95 (14.49) | | -0.11 | .92 |
| Other psychological symptoms and features |  |  | |  |  |
| Food addiction “category” (YFAS) | 100 (59.88) | 14 (70.00) | | 0.77 | .49 |
| Number of food addiction criteria met (YFAS) | 4.70 (1.78) | 5.30 (1.92) | | 1.33 | .20 |
| Emotion regulation: Nonacceptance (DERS) | 12.75 (5.18) | 12.20 (5.46) | | 0.68 | .50 |
| Emotion regulation: Difficulties with goals (DERS) | 12.29 (5.31) | 12.45 (5.36) | | -0.43 | .67 |
| Emotion regulation: Impulse control difficulties (DERS) | 16.50 (5.49) | 18.80 (5.79) | | 0.12 | .90 |
| Emotion regulation: Lack of awareness (DERS) | 15.45 (6.51) | 15.90 (7.66) | | 1.67 | .11 |
| Emotion regulation: Limited access to strategies (DERS) | 10.26 (3.68) | 10.70 (4.00) | | 0.25 | .80 |
| Emotion regulation: Lack of clarity (DERS) | 3.00 (0.64) | 3.14 (0.47) | | 0.46 | .65 |
| Self-control (BSCS) | 4.53 (1.25) | 5.02 (1.15) | | 1.23 | .23 |
| Weight bias internalization (WBIS) | 14.75 (8.42) | 17.35 (10.60) | | 1.78 | .09 |
| Depression score (BDI) | 19.79 (6.51) | 21.90 (6.67) | | 1.06 | .30 |
| Self-esteem (RSES) | 1.03 (0.64) | 1.20 (0.67) | | 1.34 | .19 |
| Interpersonal problems (IIP) | 2.18 (0.78) | 2.05 (0.68) | | 1.06 | .30 |
| Cognitive rumination: Brooding (RRS) | 1.92 (0.67) | 1.56 (0.46) | | -0.82 | .42 |
| **Cognitive rumination: Reflecting (RRS)** | **43.01 (10.99)** | **40.94 (12.50)** | | **-3.13** | **.004** |
| Mental health composite (SF36) | 44.19 (10.28) | 43.30 (10.66) | | -0.71 | .49 |
| Physical health composite (SF36) | 4.70 (1.78) | 5.30 (1.92) | | -0.36 | .72 |
| Treatment condition |  |  | | 3.27 | .08 |
| Standard care | 38 (22.22) | 1 (5.00) | |  |  |
| Stepped care | 133 (77.78) | 19 (95.00) | |  |  |
| **Rapid response (≥65% reduction binge-eating by week 4)** | **113 (59.33)** | **7 (38.89)** | | **6.72** | **.02** |

*Note.* SCID-I/P = Structured Clinical Interview for DSM Diagnosis, EDE = Eating Disorder Examination, TFEQ = Three Factor Eating Questionnaire, QEWP = Questionnaire on Eating and Weight Pattern, EOQ = Emotional Overeating Questionnaire, FTSI = Food Thought Suppression Inventory, YFAS = Yale Food Addiction Scale, DERS = Difficulties in Emotion Regulation Scale, BSCS = Self-Control Scale, WBIS = Weight Bias Internalization Scale, BDI = Beck Depression Inventory, RSES = Rosenberg Self-Esteem Scale, IIP = Inventory of Interpersonal Problems, RRS = Rumination Scale. Binge-eating percent reduction was log-transformed for analyses, though raw values are presented here for ease of interpretation.

**Supplemental Table 2.** *Average regression coefficients and variable importance in prediction of binge-eating abstinence*

|  | Logistic  (*b*) | Elastic net  (*b*) | Random forest (importance) |
| --- | --- | --- | --- |
| Intercept | 0.31 | 0.24 | - |
| Sex | -0.07 | -0.02 | 0.75 |
| Race | -0.32 | -0.12 | 0.66 |
| Education | 0.08 | 0 | 0.73 |
| Age (years) | 0.02 | 0.01 | 3.17 |
| Body mass index | 0.14 | 0.03 | 3.50 |
| Depressive disorders | -0.08 | -0.02 | 0.75 |
| Anxiety disorders | -0.34 | -0.08 | 0.82 |
| Posttraumatic stress disorder | -0.20 | -0.06 | 0.45 |
| Drug use disorder | -0.14 | -0.03 | 0.69 |
| Alcohol use disorder | -0.27 | -0.08 | 0.93 |
| Binge-eating frequency past month (EDE) | -0.26 | -0.06 | 3.47 |
| Overvaluation (EDE) | -0.27 | -0.06 | 2.36 |
| Dissatisfaction (EDE) | 0.46 | 0.08 | 2.28 |
| Restraint (EDE) | 0.13 | 0.01 | 2.87 |
| Restraint (TFEQ) | 0.20 | 0.04 | 2.58 |
| Bx indicator: Eat rapidly (QEWP) | -0.22 | -0.05 | 0.74 |
| Bx indicator: Eat until uncomfortably full (QEWP) | 0.21 | 0.06 | 0.45 |
| Bx indicator: Eat alone because embarrassed (QEWP) | 0.07 | 0.01 | 0.76 |
| Distress about binge eating (QEWP) | -0.13 | -0.03 | 1.76 |
| Weight cycling (QEWP) | -0.46 | -0.13 | 1.74 |
| Diet history (QEWP) | 0.04 | 0.03 | 1.83 |
| Emotional overeating (EOQ) | 0.03 | 0.01 | 3.12 |
| Food thought suppression (FTSI) | 0.04 | 0 | 3.23 |
| Food addiction “category” (YFAS) | -0.04 | -0.01 | 0.70 |
| Number of food addiction criteria met (YFAS) | 0.02 | 0 | 2.45 |
| Emotion regulation: Nonacceptance (DERS) | -0.43 | -0.10 | 2.78 |
| Emotion regulation: Difficulties with goals (DERS) | 0.14 | 0.05 | 2.79 |
| Emotion regulation: Impulse control difficulties (DERS) | -0.48 | -0.07 | 2.68 |
| Emotion regulation: Lack of awareness (DERS) | -0.99 | -0.18 | 3.43 |
| Emotion regulation: Limited access to strategies (DERS) | 0.63 | 0.07 | 3.04 |
| Emotion regulation: Lack of clarity (DERS) | 0.92 | 0.13 | 2.84 |
| Self-control (BSCS) | 0.06 | 0.01 | 3.65 |
| Weight bias internalization (WBIS) | -0.68 | -0.16 | 3.68 |
| Depression score (BDI) | 0.61 | 0.06 | 3.13 |
| Self-esteem (RSES) | 0.03 | -0.02 | 3.06 |
| Interpersonal problems (IIP) | 0 | 0 | 3.68 |
| Cognitive rumination: Brooding (RRS) | -0.27 | -0.03 | 2.68 |
| Cognitive rumination: Reflecting (RRS) | -0.09 | -0.01 | 2.79 |
| Mental health composite (SF36) | 0.07 | -0.01 | 3.56 |
| Physical health composite (SF36) | 0.19 | 0.02 | 3.69 |
| Treatment group | 0.21 | 0.03 | 0.69 |
| Rapid response | 0.14 | 0.06 | 0.91 |

*Note.* EDE = Eating Disorder Examination, TFEQ = Three Factor Eating Questionnaire, Bx ind = behavioral indicator for binge-eating disorder diagnosis, QEWP = Questionnaire on Eating and Weight Pattern, EOQ = Emotional Overeating Questionnaire, FTSI = Food Thought Suppression Inventory, YFAS = Yale Food Addiction Scale, DERS = Difficulties in Emotion Regulation Scale, BSCS = Self-Control Scale, WBIS = Weight Bias Internalization Scale, BDI = Beck Depression Inventory, RSES = Rosenberg Self-Esteem Scale, IIP = Inventory of Interpersonal Problems, RRS = Rumination Scale.

**Supplemental Table 3.** *Average regression coefficients and variable importance in prediction of binge-eating reduction (%)*

|  | Logistic  (*b*) | Elastic net  (*b*) | Random forest (importance) |
| --- | --- | --- | --- |
| Intercept | 5.62 | 0 | 0 |
| Sex | -0.01 | 0 | 0.05 |
| Race | 0.02 | 0 | 0.07 |
| Education | -0.02 | 0 | 0.13 |
| Age (years) | -0.03 | 0 | 0.64 |
| Body mass index | -0.02 | -0.01 | 1.01 |
| Depressive disorders | 0.02 | 0 | 0.15 |
| Anxiety disorders | -0.01 | 0 | 0.07 |
| Posttraumatic stress disorder | 0.03 | 0 | 0.02 |
| Drug use disorder | -0.02 | 0 | 0.07 |
| Alcohol use disorder | 0.03 | 0 | 0.03 |
| Binge-eating frequency past month (EDE) | 0.09 | 0.05 | 3.62 |
| Overvaluation (EDE) | -0.03 | -0.01 | 0.63 |
| Dissatisfaction (EDE) | 0.10 | 0.03 | 1.38 |
| Restraint (EDE) | -0.01 | 0 | 0.27 |
| Restraint (TFEQ) | 0.03 | 0 | 0.69 |
| Bx indicator: Eat rapidly (QEWP) | -0.05 | -0.01 | 0.06 |
| Bx indicator: Eat until uncomfortably full (QEWP) | 0 | 0 | 0 |
| Bx indicator: Eat alone because embarrassed (QEWP) | 0.06 | 0.01 | 0.19 |
| Distress about binge eating (QEWP) | -0.04 | -0.01 | 0.20 |
| Weight cycling (QEWP) | -0.04 | -0.01 | 0.20 |
| Diet history (QEWP) | -0.04 | -0.01 | 0.59 |
| Emotional overeating (EOQ) | 0.03 | 0.01 | 0.58 |
| Food thought suppression (FTSI) | 0.04 | 0 | 0.56 |
| Food addiction “category” (YFAS) | 0.04 | 0 | 0.16 |
| Number of food addiction criteria met (YFAS) | -0.05 | 0 | 0.35 |
| Emotion regulation: Nonacceptance (DERS) | 0 | 0 | 0.65 |
| Emotion regulation: Difficulties with goals (DERS) | -0.12 | -0.02 | 0.80 |
| Emotion regulation: Impulse control difficulties (DERS) | 0.09 | 0.01 | 0.71 |
| Emotion regulation: Lack of awareness (DERS) | -0.03 | 0 | 0.66 |
| Emotion regulation: Limited access to strategies (DERS) | -0.01 | 0 | 0.47 |
| Emotion regulation: Lack of clarity (DERS) | 0.06 | 0.01 | 0.50 |
| Self-control (BSCS) | 0.01 | 0 | 0.64 |
| Weight bias internalization (WBIS) | -0.02 | 0 | 0.34 |
| Depression score (BDI) | 0 | 0 | 1.27 |
| Self-esteem (RSES) | -0.04 | 0 | 1.23 |
| Interpersonal problems (IIP) | 0.02 | 0 | 0.87 |
| Cognitive rumination: Brooding (RRS) | 0.04 | 0 | 0.37 |
| Cognitive rumination: Reflecting (RRS) | -0.13 | -0.03 | 1.15 |
| Mental health composite (SF36) | -0.03 | -0.01 | 2.70 |
| Physical health composite (SF36) | 0.02 | 0 | 0.47 |
| Treatment group | 0.08 | 0.02 | 0.52 |
| Rapid response | 0.08 | 0.02 | 0.43 |

*Note.* EDE = Eating Disorder Examination, TFEQ = Three Factor Eating Questionnaire, Bx ind = behavioral indicator for binge-eating disorder diagnosis, QEWP = Questionnaire on Eating and Weight Pattern, EOQ = Emotional Overeating Questionnaire, FTSI = Food Thought Suppression Inventory, YFAS = Yale Food Addiction Scale, DERS = Difficulties in Emotion Regulation Scale, BSCS = Self-Control Scale, WBIS = Weight Bias Internalization Scale, BDI = Beck Depression Inventory, RSES = Rosenberg Self-Esteem Scale, IIP = Inventory of Interpersonal Problems, RRS = Rumination Scale.

**Supplemental Table 4.** *Average* *regression coefficients and variable importance in prediction of eating-disorder psychopathology*

|  | Logistic  (*b*) | Elastic net  (*b*) | Random forest (importance) |
| --- | --- | --- | --- |
| Intercept | 1.77 | 0 | 0 |
| Sex | 0.01 | -0.01 | 0.75 |
| Race | -0.05 | -0.01 | 0.45 |
| Education | 0.05 | 0.01 | 0.78 |
| Age (years) | -0.06 | -0.02 | 3.68 |
| Body mass index | 0 | 0 | 3.50 |
| Depressive disorders | 0.10 | 0.06 | 1.25 |
| Anxiety disorders | -0.03 | -0.01 | 0.79 |
| Posttraumatic stress disorder | -0.09 | -0.03 | 0.28 |
| Drug use disorder | 0.02 | 0 | 0.39 |
| Alcohol use disorder | 0.06 | 0.01 | 0.62 |
| Binge-eating frequency past month (EDE) | 0 | 0 | 4.01 |
| Overvaluation (EDE) | 0.08 | 0.07 | 4.00 |
| Dissatisfaction (EDE) | -0.09 | 0 | 3.41 |
| Restraint (EDE) | 0.07 | 0.03 | 3.34 |
| Restraint (TFEQ) | 0.07 | 0.04 | 3.24 |
| Bx indicator: Eat rapidly (QEWP) | -0.02 | 0 | 0.57 |
| Bx indicator: Eat until uncomfortably full (QEWP) | -0.07 | -0.03 | 0.47 |
| Bx indicator: Eat alone because embarrassed (QEWP) | -0.04 | 0 | 0.82 |
| Distress about binge eating (QEWP) | 0.05 | 0.01 | 1.80 |
| Weight cycling (QEWP) | -0.01 | 0 | 1.56 |
| Diet history (QEWP) | 0.07 | 0.05 | 2.48 |
| Emotional overeating (EOQ) | 0.09 | 0.02 | 4.08 |
| Food thought suppression (FTSI) | 0.08 | 0.06 | 4.96 |
| Food addiction “category” (YFAS) | -0.07 | 0 | 1.07 |
| Number of food addiction criteria met (YFAS) | 0.13 | 0.06 | 3.48 |
| Emotion regulation: Nonacceptance (DERS) | 0.19 | 0.07 | 4.25 |
| Emotion regulation: Difficulties with goals (DERS) | 0.03 | 0 | 3.28 |
| Emotion regulation: Impulse control difficulties (DERS) | 0.02 | 0 | 2.84 |
| Emotion regulation: Lack of awareness (DERS) | 0.08 | 0 | 2.84 |
| Emotion regulation: Limited access to strategies (DERS) | -0.10 | 0 | 3.39 |
| Emotion regulation: Lack of clarity (DERS) | -0.14 | -0.03 | 2.49 |
| Self-control (BSCS) | 0.03 | 0 | 4.45 |
| Weight bias internalization (WBIS) | 0.23 | 0.14 | 7.90 |
| Depression score (BDI) | -0.10 | 0 | 5.36 |
| Self-esteem (RSES) | 0.22 | 0.07 | 5.67 |
| Interpersonal problems (IIP) | -0.12 | -0.01 | 4.06 |
| Cognitive rumination: Brooding (RRS) | 0.07 | 0.04 | 4.76 |
| Cognitive rumination: Reflecting (RRS) | 0.04 | 0.01 | 3.85 |
| Mental health composite (SF36) | 0 | 0 | 4.97 |
| Physical health composite (SF36) | -0.02 | 0 | 4.68 |
| Treatment group | -0.06 | -0.01 | 0.47 |
| Rapid response | -0.02 | 0 | 0.65 |

*Note.* EDE = Eating Disorder Examination, TFEQ = Three Factor Eating Questionnaire, Bx ind = behavioral indicator for binge-eating disorder diagnosis, QEWP = Questionnaire on Eating and Weight Pattern, EOQ = Emotional Overeating Questionnaire, FTSI = Food Thought Suppression Inventory, YFAS = Yale Food Addiction Scale, DERS = Difficulties in Emotion Regulation Scale, BSCS = Self-Control Scale, WBIS = Weight Bias Internalization Scale, BDI = Beck Depression Inventory, RSES = Rosenberg Self-Esteem Scale, IIP = Inventory of Interpersonal Problems, RRS = Rumination Scale.

**Supplemental Table 5.** *Average regression coefficients and variable importance in prediction of weight loss ≥ 5%*

|  | Logistic  (*b*) | Elastic net  (*b*) | Random forest (importance) |
| --- | --- | --- | --- |
| Intercept | -0.49 | -0.32 | 0 |
| Sex | 0.27 | 0.09 | 0.90 |
| Race | 0.29 | 0.11 | 0.78 |
| Education | 0.03 | -0.01 | 0.83 |
| Age (years) | 0.43 | 0.17 | 3.53 |
| Body mass index | -0.17 | -0.02 | 3.41 |
| Depressive disorders | -0.34 | -0.04 | 0.74 |
| Anxiety disorders | -0.01 | 0 | 0.66 |
| Posttraumatic stress disorder | -0.22 | -0.11 | 0.50 |
| Drug use disorder | 0.57 | 0.12 | 0.76 |
| Alcohol use disorder | -0.12 | 0 | 0.77 |
| Binge-eating frequency past month (EDE) | 0.51 | 0.12 | 3.13 |
| Overvaluation (EDE) | -0.20 | -0.11 | 2.69 |
| Dissatisfaction (EDE) | 0.77 | 0.17 | 2.67 |
| Restraint (EDE) | 0.04 | 0.02 | 2.79 |
| Restraint (TFEQ) | 0.34 | 0.07 | 2.54 |
| Bx indicator: Eat rapidly (QEWP) | 0.04 | 0 | 0.73 |
| Bx indicator: Eat until uncomfortably full (QEWP) | 0.11 | 0.01 | 0.42 |
| Bx indicator: Eat alone because embarrassed (QEWP) | -0.50 | -0.13 | 1.14 |
| Distress about binge eating (QEWP) | -0.73 | -0.24 | 2.26 |
| Weight cycling (QEWP) | 0.51 | 0.12 | 1.59 |
| Diet history (QEWP) | -0.02 | -0.01 | 1.92 |
| Emotional overeating (EOQ) | -0.27 | -0.06 | 3.18 |
| Food thought suppression (FTSI) | -0.07 | -0.02 | 3.26 |
| Food addiction “category” (YFAS) | 0.34 | 0.03 | 0.70 |
| Number of food addiction criteria met (YFAS) | -0.32 | -0.02 | 2.00 |
| Emotion regulation: Nonacceptance (DERS) | 0.14 | 0.01 | 2.46 |
| Emotion regulation: Difficulties with goals (DERS) | 0.64 | 0.07 | 3.05 |
| Emotion regulation: Impulse control difficulties (DERS) | -0.24 | -0.02 | 2.59 |
| Emotion regulation: Lack of awareness (DERS) | -0.51 | -0.03 | 3.26 |
| Emotion regulation: Limited access to strategies (DERS) | 0.03 | 0.01 | 2.56 |
| Emotion regulation: Lack of clarity (DERS) | 1.06 | 0.14 | 2.81 |
| Self-control (BSCS) | 0 | -0.01 | 2.96 |
| Weight bias internalization (WBIS) | - 0.04 | 0 | 3.31 |
| Depression score (BDI) | 0.41 | 0.01 | 2.81 |
| Self-esteem (RSES) | -0.15 | -0.02 | 2.75 |
| Interpersonal problems (IIP) | 0.26 | 0.05 | 3.48 |
| Cognitive rumination: Brooding (RRS) | -0.92 | -0.15 | 2.87 |
| Cognitive rumination: Reflecting (RRS) | 0.12 | 0 | 2.55 |
| Mental health composite (SF36) | 0.37 | 0.04 | 3.60 |
| Physical health composite (SF36) | 0.21 | 0 | 3.47 |
| Treatment group | 0.12 | 0 | 0.67 |
| Rapid response | 0.75 | 0.31 | 2.03 |

*Note.* EDE = Eating Disorder Examination, TFEQ = Three Factor Eating Questionnaire, Bx ind = behavioral indicator for binge-eating disorder diagnosis, QEWP = Questionnaire on Eating and Weight Pattern, EOQ = Emotional Overeating Questionnaire, FTSI = Food Thought Suppression Inventory, YFAS = Yale Food Addiction Scale, DERS = Difficulties in Emotion Regulation Scale, BSCS = Self-Control Scale, WBIS = Weight Bias Internalization Scale, BDI = Beck Depression Inventory, RSES = Rosenberg Self-Esteem Scale, IIP = Inventory of Interpersonal Problems, RRS = Rumination Scale.

**Supplemental Table 6.** *Average regression coefficients and variable importance in prediction of weight loss (%)*

|  | Logistic  (*b*) | Elastic net  (*b*) | Random forest (importance) |
| --- | --- | --- | --- |
| Intercept | 4.21 | 0 | 0 |
| Sex | 1.29 | 1.07 | 176.58 |
| Race | 0.44 | 0.3 | 42.33 |
| Education | 0.55 | 0.43 | 63.23 |
| Age (years) | 0.63 | 0.56 | 299.37 |
| Body mass index | -0.04 | 0 | 342.80 |
| Depressive disorders | -0.87 | -0.62 | 64.99 |
| Anxiety disorders | -0.09 | -0.01 | 51.42 |
| Posttraumatic stress disorder | -0.48 | -0.48 | 47.10 |
| Drug use disorder | 0.89 | 0.55 | 58.20 |
| Alcohol use disorder | -0.21 | -0.01 | 80.78 |
| Binge-eating frequency past month (EDE) | 1.05 | 0.78 | 279.18 |
| Overvaluation (EDE) | -1.30 | -1.10 | 324.39 |
| Dissatisfaction (EDE) | 1.73 | 1.54 | 261.51 |
| Restraint (EDE) | -0.41 | -0.28 | 249.79 |
| Restraint (TFEQ) | 0.69 | 0.52 | 281.63 |
| Bx indicator: Eat rapidly (QEWP) | 0.34 | 0.22 | 45.35 |
| Bx indicator: Eat until uncomfortably full (QEWP) | -0.42 | -0.31 | 38.76 |
| Bx indicator: Eat alone because embarrassed (QEWP) | -0.57 | -0.39 | 76.53 |
| Distress about binge eating (QEWP) | -1.57 | -1.33 | 206.34 |
| Weight cycling (QEWP) | 0.28 | 0.26 | 117.43 |
| Diet history (QEWP) | 0.54 | 0.39 | 139.53 |
| Emotional overeating (EOQ) | -0.08 | 0 | 274.06 |
| Food thought suppression (FTSI) | 0.45 | 0.14 | 354.79 |
| Food addiction “category” (YFAS) | 0.68 | 0.40 | 50.60 |
| Number of food addiction criteria met (YFAS) | -1.78 | -1.34 | 193.68 |
| Emotion regulation: Nonacceptance (DERS) | 0.66 | 0.40 | 213.37 |
| Emotion regulation: Difficulties with goals (DERS) | 1.25 | 0.85 | 281.67 |
| Emotion regulation: Impulse control difficulties (DERS) | -0.68 | -0.18 | 236.77 |
| Emotion regulation: Lack of awareness (DERS) | -2.09 | -1.45 | 308.8 |
| Emotion regulation: Limited access to strategies (DERS) | 0.29 | 0.07 | 235.23 |
| Emotion regulation: Lack of clarity (DERS) | 2.68 | 1.90 | 264.22 |
| Self-control (BSCS) | 0.16 | 0.01 | 378.27 |
| Weight bias internalization (WBIS) | 0.65 | 0.37 | 306.76 |
| Depression score (BDI) | 2.02 | 1.14 | 271.88 |
| Self-esteem (RSES) | -0.62 | -0.20 | 229.99 |
| Interpersonal problems (IIP) | 0.53 | 0.44 | 306.17 |
| Cognitive rumination: Brooding (RRS) | -3.27 | -2.23 | 290.79 |
| Cognitive rumination: Reflecting (RRS) | 0.98 | 0.67 | 257.02 |
| Mental health composite (SF36) | 0.15 | 0 | 317.56 |
| Physical health composite (SF36) | 1.01 | 0.57 | 363.51 |
| Treatment group | 0.30 | 0.17 | 40.02 |
| Rapid response | 1.67 | 1.56 | 203.11 |

*Note.* EDE = Eating Disorder Examination, TFEQ = Three Factor Eating Questionnaire, Bx ind = behavioral indicator for binge-eating disorder diagnosis, QEWP = Questionnaire on Eating and Weight Pattern, EOQ = Emotional Overeating Questionnaire, FTSI = Food Thought Suppression Inventory, YFAS = Yale Food Addiction Scale, DERS = Difficulties in Emotion Regulation Scale, BSCS = Self-Control Scale, WBIS = Weight Bias Internalization Scale, BDI = Beck Depression Inventory, RSES = Rosenberg Self-Esteem Scale, IIP = Inventory of Interpersonal Problems, RRS = Rumination Scale.

**Supplemental Table 7.** *Model accuracy for logistic and linear regressions with 10 predictors*

|  | AUC (95% CI) |  |
| --- | --- | --- |
| Binge-eating abstinence |  |  |
| 10 repeated CV | .52 (.50, .55) |  |
| Bootstrap | .52 (.51, .53) |  |
| Optimism bootstrap | .55 (.54, .56) |  |
| Weight reduction ≥ 5% |  |  |
| 10 repeated CV | .68 (.65, .70) |  |
| Bootstrap | .66 (.65, .67) |  |
| Optimism bootstrap | .69 (.68, .70) |  |
|  | RMSE (95% CI) | *R*^2^ (95% CI) |
| Binge-eating reduction % (log) |  |  |
| 10 repeated CV | 0.33 (.26, .39) | .04 (.03, .05) |
| Bootstrap | 0.41 (.37, .46) | .006 (.004, .008) |
| Optimism bootstrap | 0.48 (.43, .52) | .030 (.028, .032) |
| Eating-disorder psychopathology |  |  |
| 10 repeated CV | 0.76 (0.73, 0.79) | .20 (.17, .23) |
| Bootstrap | 0.79 (0.78, 0.81) | .14 (.13, .16) |
| Optimism bootstrap | 0.77 (0.76, 0.78) | .16 (.15, .17) |
| Weight reduction % |  |  |
| 10 repeated CV | 7.03 (6.85, 7.21) | .11 (.09, .13) |
| Bootstrap | 7.36 (7.24, 7.48) | .053 (.045, .061) |
| Optimism bootstrap | 7.14 (7.02, 7.26) | .058 (.05, .066) |

*Note.* AUC = area under the receiver operator characteristic curve, RMSE = root mean square error, 10 repeated CV = repeated 10-fold cross-validation. Higher AUC values indicate greater predictive accuracy; lower RMSE values and higher *R^2^* values indicate greater predictive accuracy.

**Supplemental Table 8.** *Results of logistic regression predicting binge-eating abstinence*

|  | *b* | *SE* | *t* | *p* |
| --- | --- | --- | --- | --- |
| (Intercept) | 2.02 | 1.55 | 1.31 | 0.19 |
| Sex (1 = male) | -0.24 | 0.35 | -0.70 | 0.48 |
| Race (1 = white) | -0.83 | 0.47 | -1.77 | 0.08 |
| BMI | 0.01 | 0.03 | 0.50 | 0.62 |
| Binge-eating frequency | -0.24 | 0.26 | -0.94 | 0.35 |
| Overvaluation | -0.20 | 0.19 | -1.07 | 0.29 |
| EDE Global (7-item) | 0.07 | 0.18 | 0.40 | 0.69 |
| Any comorbid diagnosis (1 = yes) | -0.28 | 0.37 | -0.76 | 0.45 |
| BDI total | -0.02 | 0.02 | -1.08 | 0.28 |
| Group (1 = Stepped Care) | 0.05 | 0.38 | 0.13 | 0.90 |
| Rapid response (1 = rapid) | 0.43 | 0.33 | 1.30 | 0.19 |

*Note.* BMI = body mass index, EDE = Eating Disorder Examination, BDI = Beck Depression Inventory

**Supplemental Table 9.** *Results of logistic regression predicting weight reduction ≥ 5%*

|  | *b* | *SE* | *t* | *p* |
| --- | --- | --- | --- | --- |
| (Intercept) | -2.47 | 1.64 | -1.51 | 0.13 |
| **Sex (1 = male)** | **0.79** | **0.38** | **2.10** | **0.04** |
| **Race (1 = white)** | **1.13** | **0.48** | **2.36** | **0.02** |
| BMI | 0.01 | 0.03 | 0.27 | 0.78 |
| Binge-eating frequency | 0.29 | 0.27 | 1.05 | 0.29 |
| **Overvaluation** | **-0.52** | **0.21** | **-2.51** | **0.01** |
| **EDE Global (7-item)** | **0.41** | **0.20** | **2.05** | **0.04** |
| Any comorbid diagnosis (1 = yes) | 0.08 | 0.39 | 0.19 | 0.85 |
| BDI total | -0.01 | 0.02 | -0.59 | 0.56 |
| Group (1 = Stepped Care) | -0.28 | 0.41 | -0.69 | 0.49 |
| **Rapid response (1 = rapid)** | **1.55** | **0.38** | **4.05** | **< .001** |

*Note.* BMI = body mass index, EDE = Eating Disorder Examination, BDI = Beck Depression Inventory

**Supplemental Table 10.** *Results of linear regression predicting percent binge-eating reduction*

|  | *b* | *SE* | *t* | *p* |
| --- | --- | --- | --- | --- |
| (Intercept) | 283.96 | 26.88 | 10.56 | < .001 |
| Sex (1 = male) | 3.43 | 6.22 | 0.55 | 0.58 |
| Race (1 = white) | -3.58 | 7.68 | -0.47 | 0.64 |
| BMI | -0.59 | 0.47 | -1.24 | 0.21 |
| Binge-eating frequency | 8.28 | 4.52 | 1.83 | 0.07 |
| Overvaluation | -2.63 | 3.32 | -0.79 | 0.43 |
| EDE Global (7-item) | 2.05 | 3.26 | 0.63 | 0.53 |
| Any comorbid diagnosis (1 = yes) | 0.95 | 6.50 | 0.15 | 0.88 |
| BDI total | -0.26 | 0.35 | -0.72 | 0.47 |
| Group (1 = Stepped Care) | 3.28 | 6.82 | 0.48 | 0.63 |
| **Rapid response (1 = rapid)** | **13.01** | **5.92** | **2.20** | **0.03** |

*Note.* BMI = body mass index, EDE = Eating Disorder Examination, BDI = Beck Depression Inventory

**Supplemental Table 11.** *Results of linear regression predicting eating-disorder psychopathology*

|  | *b* | *SE* | *t* | *p* |
| --- | --- | --- | --- | --- |
| (Intercept) | 0.42 | 0.55 | 0.77 | 0.44 |
| Sex (1 = male) | -0.18 | 0.13 | -1.45 | 0.15 |
| Race (1 = white) | -0.02 | 0.16 | -0.15 | 0.88 |
| BMI | 0.00 | 0.01 | -0.42 | 0.68 |
| Binge-eating frequency | 0.05 | 0.09 | 0.53 | 0.60 |
| Overvaluation | 0.13 | 0.07 | 1.89 | 0.06 |
| **EDE Global (7-item)** | **0.14** | **0.07** | **2.06** | **0.04** |
| Any comorbid diagnosis (1 = yes) | 0.26 | 0.13 | 2.00 | 0.05 |
| **BDI total** | **0.02** | **0.01** | **2.24** | **0.03** |
| Group (1 = Stepped Care) | -0.06 | 0.14 | -0.42 | 0.68 |
| Rapid response (1 = rapid) | -0.05 | 0.12 | -0.40 | 0.69 |

*Note.* BMI = body mass index, EDE = Eating Disorder Examination, BDI = Beck Depression Inventory

**Supplemental Table 12.** *Results of linear regression predicting precent weight reduction*

|  | *b* | *SE* | *t* | *p* |
| --- | --- | --- | --- | --- |
| (Intercept) | -1.05 | 4.98 | -0.21 | 0.83 |
| **Sex (1 = male)** | **2.54** | **1.15** | **2.2** | **0.03** |
| Race (1 = white) | 2.16 | 1.42 | 1.52 | 0.13 |
| BMI | 0.05 | 0.09 | 0.57 | 0.57 |
| Binge-eating frequency | 0.68 | 0.84 | 0.81 | 0.42 |
| **Overvaluation** | **-1.61** | **0.61** | **-2.62** | **0.01** |
| EDE Global (7-item) | 0.86 | 0.6 | 1.42 | 0.16 |
| Any comorbid diagnosis (1 = yes) | -0.33 | 1.21 | -0.28 | 0.78 |
| BDI total | 0.06 | 0.07 | 0.98 | 0.33 |
| Group (1 = Stepped Care) | -0.25 | 1.26 | -0.20 | 0.84 |
| **Rapid response (1 = rapid)** | **3.90** | **1.10** | **3.55** | **< .001** |

*Note.* BMI = body mass index, EDE = Eating Disorder Examination, BDI = Beck Depression Inventory

R code (please contact Lauren Forrest for a copy of the R file)

# Machine learning predicting post-tx response stepped care study

# Lauren Forrest, Valentina Ivezaj, & Carlos Grilo

#----------------0. load needed packages----------------

library("haven")

library("dplyr")

library("e1071")

library("caret")

library("stats")

library("tidyverse")

library("pROC")

library("RANN")

library("boot")

library("mice")

library("Rmisc")

#----------------1. read and clean data----------------

#---------1a. create dummy vars---------

#sc2 has categorical predictors in 0/1 format (not as factors; for enets)

sc2 <- sc %>%

select(c(Age, Group, Sex, race, Ethnicity, Education, ede_ov, ede_bd, ede_rest,

bdi_tot, base.bmi, scs_tot, wbis_tot, rs_brood,

rs_ponder, yfas_crit, yfas_dx, dep_any, bipolar_any, anx_any, ptsd,

sud_any, aud, OBE.EDEInt, EOQ_tot, tsf36mcs, tsf36pcs, RSES_tot, IIP_tot,

DERS_nonaccept, DERS_goals, DERS_impulse, DERS_aware, DERS_strat, DERS_clar,

FTSI_tot, TFEQ_rest, qewp9, qewp13a, qewp13b, qewp13c, qewp13d, qewp13e, qewp16, qewp25,

rapid, rapid.itt, remit.post, remit.post.itt,

remit.post.lnf, remit.post.lnf.itt, post.OBE_pct, post.OBE_pct.itt, Total.EDEint.post, Total.EDEint.post.itt, post.wtloss.5pct,

post.wtloss.5pct.itt, post.wtloss_pct, post.wtloss_pct.itt)) %>%

dplyr::rename(group = Group,

sex = Sex,

ethn = Ethnicity,

edu = Education,

wtcycl = qewp9,

bxind_rapid = qewp13a,

bxind_full = qewp13b,

bxind_hungry = qewp13c,

bxind_alone = qewp13d,

bxind_disgust = qewp13e,

obe_distress = qewp16,

diethx = qewp25,

sf_mh = tsf36mcs,

sf_ph = tsf36pcs)

sc2$edu[is.na(sc2$edu)] <- 1

#make factors for imputation purposes, then will convert back to numeric for sc2

sc2$group <- factor(sc2$group, levels = c(0, 1))

sc2$edu <- factor(sc2$edu, levels = c(0,1))

sc2$sex <- factor(sc2$sex, levels = c(0, 1))

sc2$race <- factor(sc2$race, levels = c(0, 1))

sc2$ethn <- factor(sc2$ethn, levels = c(0, 1))

sc2$yfas_dx <- factor(sc2$yfas_dx, levels = c(0,1))

sc2$dep_any <- factor(sc2$dep_any, levels = c(0,1))

sc2$bipolar_any <- factor(sc2$bipolar_any, levels = c(0,1))

sc2$anx_any <- factor(sc2$anx_any, levels = c(0,1))

sc2$ptsd <- factor(sc2$ptsd, levels = c(0,1))

sc2$sud_any <- factor(sc2$sud_any, levels = c(0,1))

sc2$aud <- factor(sc2$aud, levels = c(0,1))

sc2$bxind_rapid <- factor(sc2$bxind_rapid, levels = c(0,1))

sc2$bxind_full <- factor(sc2$bxind_full, levels = c(0,1))

sc2$bxind_hungry <- factor(sc2$bxind_hungry, levels = c(0,1))

sc2$bxind_alone <- factor(sc2$bxind_alone, levels = c(0,1))

sc2$bxind_disgust <- factor(sc2$bxind_disgust, levels = c(0,1))

#---------1b. near-zero variance predictors---------

pred <- sc2 %>%

select(-c(remit.post, remit.post.itt, remit.post.lnf, remit.post.lnf.itt,

post.OBE_pct, post.OBE_pct.itt, Total.EDEint.post, Total.EDEint.post.itt, post.wtloss.5pct,

post.wtloss.5pct.itt, post.wtloss_pct, post.wtloss_pct.itt))

# freqCut = 10, uniqueCut = 20,

nzvar <- nearZeroVar(pred[, ],saveMetrics = TRUE)

nzvar

table(sc2$race)

table(sc2$ethn)

table(sc2$bipolar_any)

table(sc2$ptsd)

table(sc2$sud_any)

table(sc2$bxind_hungry)

table(sc2$bxind_disgust)

sc2<- sc2 %>%

select(-c(ethn, bipolar_any, bxind_hungry, bxind_disgust))

#---------1c. find highly correlated variables and assess VIF---------

str(sc2)

sc2 = as.data.frame(sc2)

sc_numeric <- sc2 %>%

select(Age, ede_ov, ede_bd, ede_rest,

bdi_tot, base.bmi,scs_tot, wbis_tot, rs_brood,

rs_ponder, yfas_crit, OBE.EDEInt, EOQ_tot, tsf36mcs, tsf36pcs, RSES_tot, IIP_tot,

DERS_nonaccept, DERS_goals, DERS_impulse, DERS_aware, DERS_strat, DERS_clar,

FTSI_tot, TFEQ_rest, wtcycl, bxind_rapid, bxind_full, bxind_alone, obe_distress, diethx,

post.OBE_pct.itt, Total.EDEint.post.itt, post.wtloss_pct.itt)

corrs <- cor(sc_numeric, use = "na.or.complete")

highCorr <- findCorrelation(corrs, cutoff = .9)

length(highCorr) #0

summary(corrs[upper.tri(corrs)]) #max corr = .80

#---------1d. skewness, only predictor w/ significant skew = OBE---------

#----transforming OBE pct and wt loss pct----

skew <- apply(sc_numeric, 2, skewness, na.rm = TRUE)

sc_numeric$OBE.EDEInt2 <- log(sc_numeric$OBE.EDEInt)

skew

sc2$OBE.EDEInt2 <- log(sc2$OBE.EDEInt)

sc2$OBE.EDEInt <- NULL

sc2$post.OBE_pct.itt <- log(sc2$post.OBE_pct.itt + 201)

#---------1e. impute ---------

imp <- mice(sc2, m=1, maxit = 50, method = 'pmm', seed = 500)

summary(imp)

sc2 <- complete(imp,1)

save(sc2, file = "sc_complete.Rdata")

sc2$group <- as.numeric(as.character(sc2$group))

sc2$edu <- as.numeric(as.character(sc2$edu))

sc2$sex <- as.numeric(as.character(sc2$sex))

sc2$race <- as.numeric(as.character(sc2$race))

sc2$yfas_dx <- as.numeric(as.character(sc2$yfas_dx))

sc2$dep_any <- as.numeric(as.character(sc2$dep_any))

sc2$anx_any <- as.numeric(as.character(sc2$anx_any))

sc2$ptsd <- as.numeric(as.character(sc2$ptsd))

sc2$sud_any <- as.numeric(as.character(sc2$sud_any))

sc2$aud <- as.numeric(as.character(sc2$aud))

sc2$bxind_rapid <- as.numeric(as.character(sc2$bxind_rapid))

sc2$bxind_full <- as.numeric(as.character(sc2$bxind_full))

sc2$bxind_alone <- as.numeric(as.character(sc2$bxind_alone))

#sc2.1 has categorical predictors as factors (for logisitc/linear regression and RFs)

sc2.1 <- sc2

sc2.1$group <- factor(sc2.1$group, levels = c(0, 1),

labels= c("standard", "stepped"))

sc2.1$edu <- factor(sc2.1$edu, levels = c(0,1),

labels = c("no_BS", "BS"))

sc2.1$sex <- factor(sc2.1$sex, levels = c(0, 1),

labels = c("woman", "man"))

sc2.1$race <- factor(sc2.1$race, levels = c(0, 1),

labels = c("Non-white", "White"))

sc2.1$yfas_dx <- factor(sc2.1$yfas_dx, levels = c(0,1),

labels = c("absent", "present"))

sc2.1$dep_any <- factor(sc2.1$dep_any, levels = c(0,1),

labels = c("absent", "present"))

sc2.1$anx_any <- factor(sc2.1$anx_any, levels = c(0,1),

labels = c("absent", "present"))

sc2.1$ptsd <- factor(sc2.1$ptsd, levels = c(0,1),

labels = c("absent", "present"))

sc2.1$sud_any <- factor(sc2.1$sud_any, levels = c(0,1),

labels = c("absent", "present"))

sc2.1$aud <- factor(sc2.1$aud, levels = c(0,1),

labels = c("absent", "present"))

sc2.1$bxind_rapid <- factor(sc2.1$bxind_rapid, levels = c(0,1),

labels = c("absent", "present"))

sc2.1$bxind_full <- factor(sc2.1$bxind_full, levels = c(0,1),

labels = c("absent", "present"))

sc2.1$bxind_alone <- factor(sc2.1$bxind_alone, levels = c(0,1),

labels = c("absent", "present"))

#2.-------------------split into individual data frames for each outcome-------------------

#ITT samples (i.e., full sample, n = 191)

remit.lnf.itt2.1 <- sc2.1 %>%

select(-c(remit.post.itt,

post.OBE_pct.itt, Total.EDEint.post.itt,

post.wtloss.5pct.itt, post.wtloss_pct.itt))

obe.pct.itt2.1 <- sc2.1 %>%

select(-c(remit.post.itt, remit.post.lnf.itt,

Total.EDEint.post.itt,

post.wtloss.5pct.itt, post.wtloss_pct.itt))

ede.itt2.1 <- sc2.1 %>%

select(-c(remit.post.itt, remit.post.lnf.itt,

post.OBE_pct.itt,

post.wtloss.5pct.itt, post.wtloss_pct.itt))

wtloss.5pct.itt2.1 <- sc2.1 %>%

select(-c(remit.post.itt,remit.post.lnf.itt,

post.OBE_pct.itt, Total.EDEint.post.itt,

post.wtloss_pct.itt))

wtloss.pct.itt2.1 <- sc2.1 %>%

select(-c(remit.post.itt, remit.post.lnf.itt,

post.OBE_pct.itt, Total.EDEint.post.itt,

post.wtloss.5pct.itt))

remit.lnf.itt2.1 <- as.data.frame(remit.lnf.itt2.1)

obe.pct.itt2.1 <- as.data.frame(obe.pct.itt2.1)

ede.itt2.1 <- as.data.frame(ede.itt2.1)

wtloss.5pct.itt2.1 <- as.data.frame(wtloss.5pct.itt2.1)

wtloss.pct.itt2.1 <- as.data.frame(wtloss.pct.itt2.1)

#-------------------3. Traditional linear/logistic regressions-------------------

#-----set control functions-----

control_cv <- trainControl(

method = "repeatedcv",

number = 10,

repeats = 10,

savePredictions = "final",

classProbs = TRUE,

selectionFunction = "oneSE",

allowParallel = TRUE,

summaryFunction = twoClassSummary)

control_boot <- trainControl(

method = "boot",

number = 100,

savePredictions = "final",

classProbs = TRUE,

selectionFunction = "oneSE",

allowParallel = TRUE,

summaryFunction = twoClassSummary)

control_opt <- trainControl(method = "optimism_boot",

number = 100,

savePredictions = "final",

classProbs = TRUE,

selectionFunction = "oneSE",

allowParallel = TRUE,

summaryFunction = twoClassSummary)

#-------------3. Logistic: categorical-------------

options(scipen = 999) #turn off scientific notation

#-----3a. remit.lnf.itt----

set.seed(825)

remit.lnf.ittFit.l <- train(remit.post.lnf.itt ~., data = remit.lnf.itt2.1,

method = "glm",

family = "binomial",

trControl = control_cv,

preProcess = c("center", "scale"),

metric = "ROC")

remit.lnf.ittFit.l

CI(remit.lnf.ittFit.l$resample$ROC, ci = 0.95)

remit.lnf.ittFit.l.df <- as.data.frame(remit.lnf.ittFit.l$finalModel$coefficients)

View(remit.lnf.ittFit.l.df)

set.seed(825)

remit.lnf.ittFit_boot.l <- train(remit.post.lnf.itt ~., data = remit.lnf.itt2.1,

method = "glm",

family = "binomial",

trControl = control_boot,

preProcess = c("center", "scale"),

metric = "ROC")

remit.lnf.ittFit_boot.l

CI(remit.lnf.ittFit_boot.l$resample$ROC, ci = 0.95)

remit.lnf.ittFit_boot.l.df <- as.data.frame(remit.lnf.ittFit_boot.l$finalModel$coefficients)

View(remit.lnf.ittFit_boot.l.df)

set.seed(825)

remit.lnf.ittFit_opt.l <- train(remit.post.lnf.itt ~., data = remit.lnf.itt2.1,

method = "glm",

family = "binomial",

trControl = control_opt,

preProcess = c("center", "scale"),

metric = "ROC")

remit.lnf.ittFit_opt.l

remit.lnf.ittFit_opt.l_me <- qt(.975, 99)*(remit.lnf.ittFit_opt.l$results$ROCSD/sqrt(100))

mean(remit.lnf.ittFit_opt.l$results$ROC) - remit.lnf.ittFit_opt.l_me

mean(remit.lnf.ittFit_opt.l$results$ROC)+remit.lnf.ittFit_opt.l_me

remit.lnf.ittFit_opt.l.df <- as.data.frame(remit.lnf.ittFit_opt.l$finalModel$coefficients)

View(remit.lnf.ittFit_opt.l.df)

#-----3b. wtloss.5pct.itt----

set.seed(825)

wtloss.5pct.ittFit.l <- train(post.wtloss.5pct.itt ~., data = wtloss.5pct.itt2.1,

method = "glm",

family = "binomial",

trControl = control_cv,

preProcess = c("center", "scale"),

metric = "ROC")

wtloss.5pct.ittFit.l

CI(wtloss.5pct.ittFit.l$resample$ROC, ci = 0.95)

wtloss.5pct.ittFit.l.df <- as.data.frame(wtloss.5pct.ittFit.l$finalModel$coefficients)

View(wtloss.5pct.ittFit.l.df)

set.seed(825)

wtloss.5pct.ittFit_boot.l <- train(post.wtloss.5pct.itt~., data = wtloss.5pct.itt2.1,

method = "glm",

family = "binomial",

trControl = control_boot,

preProcess = c("center", "scale"),

metric = "ROC")

wtloss.5pct.ittFit_boot.l

CI(wtloss.5pct.ittFit_boot.l$resample$ROC, ci = 0.95)

wtloss.5pct.ittFit_boot.l.df <- as.data.frame(wtloss.5pct.ittFit_boot.l$finalModel$coefficients)

View(wtloss.5pct.ittFit_boot.l.df)

set.seed(825)

wtloss.5pct.ittFit_opt.l <- train(post.wtloss.5pct.itt ~., data = wtloss.5pct.itt2.1,

method = "glm",

family = "binomial",

trControl = control_opt,

preProcess = c("center", "scale"),

metric = "ROC")

wtloss.5pct.ittFit_opt.l

wtloss.5pct.ittFit_opt.l_me <- qt(.975, 99)*(wtloss.5pct.ittFit_opt.l$results$ROCSD/sqrt(100))

mean(wtloss.5pct.ittFit_opt.l$results$ROC) - wtloss.5pct.ittFit_opt.l_me

mean(wtloss.5pct.ittFit_opt.l$results$ROC)+wtloss.5pct.ittFit_opt.l_me

wtloss.5pct.ittFit_opt.l.df <- as.data.frame(wtloss.5pct.ittFit_opt.l$finalModel$coefficients)

View(wtloss.5pct.ittFit_opt.l.df)

#-------------------4.elastic nets: categorical-------------------

remit.lnf.itt <- sc2 %>%

select(-c(remit.post.itt,

post.OBE_pct.itt, Total.EDEint.post.itt,

post.wtloss.5pct.itt, post.wtloss_pct.itt))

wtloss.5pct.itt <- sc2 %>%

select(-c(remit.post.itt, remit.post.lnf.itt,

post.OBE_pct.itt,Total.EDEint.post.itt,

post.wtloss_pct.itt))

remit.lnf.itt <- as.data.frame(remit.lnf.itt)

wtloss.5pct.itt <- as.data.frame(wtloss.5pct.itt)

remit.lnf.itt <- remit.lnf.itt %>%

mutate(rapid = ifelse(rapid.itt == "nonrapid", 0, 1)) %>%

select(-rapid.itt)

wtloss.5pct.itt <- wtloss.5pct.itt %>%

mutate(rapid = ifelse(rapid.itt == "nonrapid", 0, 1)) %>%

select(-rapid.itt)

enetGrid <- expand.grid (.alpha = c (0, .1, .2, .4, .6, .8, 1), .lambda = seq (.01, .2, length = 40))

#-----4a. remit.lnf.itt----

set.seed(825)

remit.lnf.ittFit <- train(remit.post.lnf.itt ~., data = remit.lnf.itt,

method = "glmnet",

family = "binomial",

trControl = control_cv,

tuneGrid = enetGrid,

preProcess = c("center", "scale"),

metric = "ROC")

remit.lnf.ittFit$bestTune

mean(remit.lnf.ittFit$resample$ROC)

CI(remit.lnf.ittFit$resample$ROC, ci = 0.95)

remit.lnf.ittFit_coef <- predict(remit.lnf.ittFit$finalModel, s = remit.lnf.ittFit$finalModel$lambdaOpt, type = "coefficients")

remit.lnf.ittFit_coef <- as.data.frame(as.matrix(remit.lnf.ittFit_coef))

View(remit.lnf.ittFit$results) #bestTune ROC = .53

set.seed(825)

remit.lnf.ittFit_boot <- train(remit.post.lnf.itt ~., data = remit.lnf.itt,

method = "glmnet",

family = "binomial",

trControl = control_boot,

tuneGrid = enetGrid,

preProcess = c("center", "scale"),

metric = "ROC")

remit.lnf.ittFit_boot$bestTune

mean(remit.lnf.ittFit_boot$resample$ROC)

CI(remit.lnf.ittFit_boot$resample$ROC, ci = 0.95)

remit.lnf.ittFit_boot_coef<-predict(remit.lnf.ittFit_boot$finalModel, s = remit.lnf.ittFit_boot$finalModel$lambdaOpt, type = "coefficients")

remit.lnf.ittFit_boot_coef <- as.data.frame(as.matrix(remit.lnf.ittFit_boot_coef))

View(remit.lnf.ittFit_boot$results) #bestTune ROC = .54

set.seed(825)

remit.lnf.ittFit_opt <- train(remit.post.lnf.itt ~., data = remit.lnf.itt,

method = "glmnet",

family = "binomial",

trControl = control_opt,

tuneGrid = enetGrid,

preProcess = c("center", "scale"),

metric = "ROC")

remit.lnf.ittFit_opt$bestTune

View(remit.lnf.ittFit_opt$results) #bestTune ROC = .59

remit.lnf.ittFit_opt_me <- qt(.975, 99)*(.06/sqrt(100))

.59 - remit.lnf.ittFit_opt_me

.59 + remit.lnf.ittFit_opt_me

remit.lnf.ittFit_opt_coef <- predict(remit.lnf.ittFit_opt$finalModel, s = remit.lnf.ittFit_opt$finalModel$lambdaOpt, type = "coefficients")

remit.lnf.ittFit_opt_coef <- as.data.frame(as.matrix(remit.lnf.ittFit_opt_coef))

#-----4b. wtloss.5pct.itt----

set.seed(825)

wtloss.5pct.ittFit <- train(post.wtloss.5pct.itt ~., data = wtloss.5pct.itt,

method = "glmnet",

family = "binomial",

trControl = control_cv,

tuneGrid = enetGrid,

preProcess = c("center", "scale"),

metric = "ROC")

wtloss.5pct.ittFit$bestTune

mean(wtloss.5pct.ittFit$resample$ROC)

CI(wtloss.5pct.ittFit$resample$ROC, ci = 0.95)

wtloss.5pct.ittFit_coef <- predict(wtloss.5pct.ittFit$finalModel, s = wtloss.5pct.ittFit$finalModel$lambdaOpt, type = "coefficients")

wtloss.5pct.ittFit_coef <- as.data.frame(as.matrix(wtloss.5pct.ittFit_coef))

View(wtloss.5pct.ittFit$results) #bestTune ROC = .65

set.seed(825)

wtloss.5pct.ittFit_boot <- train(post.wtloss.5pct.itt~., data = wtloss.5pct.itt,

method = "glmnet",

family = "binomial",

trControl = control_boot,

tuneGrid = enetGrid,

preProcess = c("center", "scale"),

metric = "ROC")

wtloss.5pct.ittFit_boot$bestTune

mean(wtloss.5pct.ittFit_boot$resample$ROC)

CI(wtloss.5pct.ittFit_boot$resample$ROC, ci = 0.95)

wtloss.5pct.ittFit_boot_coef <- predict(wtloss.5pct.ittFit_boot$finalModel, s = wtloss.5pct.ittFit_boot$finalModel$lambdaOpt, type = "coefficients")

wtloss.5pct.ittFit_boot_coef <- as.data.frame(as.matrix(wtloss.5pct.ittFit_boot_coef))

View(wtloss.5pct.ittFit_boot$results) #bestTune ROC = .62

set.seed(825)

wtloss.5pct.ittFit_opt <- train(post.wtloss.5pct.itt ~., data = wtloss.5pct.itt,

method = "glmnet",

family = "binomial",

trControl = control_opt,

tuneGrid = enetGrid,

preProcess = c("center", "scale"),

metric = "ROC")

wtloss.5pct.ittFit_opt$bestTune

View(wtloss.5pct.ittFit_opt$results) #bestTune ROC = .68

wtloss.5pct.ittFit_opt_me <- qt(.975, 99)*(.06/sqrt(100))

.73 - wtloss.5pct.ittFit_opt_me

.73 + wtloss.5pct.ittFit_opt_me

wtloss.5pct.ittFit_opt_coef <- predict(wtloss.5pct.ittFit_opt$finalModel, s = wtloss.5pct.ittFit_opt$finalModel$lambdaOpt, type = "coefficients")

wtloss.5pct.ittFit_opt_coef <- as.data.frame(as.matrix(wtloss.5pct.ittFit_opt_coef))

#-----------------5. Random forests: categorical-----------------

rfGrid <- expand.grid(mtry = seq(2, 40, length = 6))

#-----5a. remit.lnf.itt----

set.seed(825)

remit.lnf.ittFit_rf <- train(remit.post.lnf.itt ~., data = remit.lnf.itt2.1,

method = "rf",

ntree = 500,

trControl = control_cv,

tuneGrid = rfGrid,

preProcess = c("center", "scale"),

metric = "ROC")

remit.lnf.ittFit_rf #bestTune mtry = 2, ROC = .56

CI(remit.lnf.ittFit_rf$resample$ROC, ci = 0.95)

remit.lnf.ittFit_rf.df <- as.data.frame(varImp(remit.lnf.ittFit_rf$finalModel))

View(remit.lnf.ittFit_rf.df)

set.seed(825)

remit.lnf.ittFit_boot_rf <- train(remit.post.lnf.itt ~., data = remit.lnf.itt2.1,

method = "rf",

ntree = 500,

trControl = control_boot,

tuneGrid = rfGrid,

preProcess = c("center", "scale"),

metric = "ROC")

remit.lnf.ittFit_boot_rf #bestTune mtry = 2, ROC = .56

CI(remit.lnf.ittFit_boot_rf$resample$ROC, ci = 0.95)

remit.lnf.ittFit_boot_rf.df <- as.data.frame(varImp(remit.lnf.ittFit_boot_rf$finalModel))

View(remit.lnf.ittFit_boot_rf.df)

set.seed(825)

remit.lnf.ittFit_opt_rf <- train(remit.post.lnf.itt ~., data = remit.lnf.itt2.1,

method = "rf",

ntree = 500,

trControl = control_opt,

tuneGrid = rfGrid,

preProcess = c("center", "scale"),

metric = "ROC")

remit.lnf.ittFit_opt_rf #bestTune mtry = 2, ROC = .94

View(remit.lnf.ittFit_opt_rf$results)

remit.lnf.ittFit_opt_rf_me <- qt(.975, 99)*(.05/sqrt(100))

.93- remit.lnf.ittFit_opt_rf_me

.93+remit.lnf.ittFit_opt_rf_me

remit.lnf.ittFit_opt_rf.df <- as.data.frame(varImp(remit.lnf.ittFit_opt_rf$finalModel))

View(remit.lnf.ittFit_opt_rf.df)

#----5b. wtloss.5pct.itt----

set.seed(825)

wtloss.5pct.ittFit_rf <- train(post.wtloss.5pct.itt ~., data = wtloss.5pct.itt2.1,

method = "rf",

ntree = 500,

trControl = control_cv,

tuneGrid = rfGrid,

preProcess = c("center", "scale"),

metric = "ROC")

wtloss.5pct.ittFit_rf #bestTune mtry = 2, ROC = .60

CI(wtloss.5pct.ittFit_rf$resample$ROC, ci = 0.95)

wtloss.5pct.ittFit_rf.df <- as.data.frame(varImp(wtloss.5pct.ittFit_rf$finalModel))

View(wtloss.5pct.ittFit_rf.df)

set.seed(825)

wtloss.5pct.ittFit_boot_rf <- train(post.wtloss.5pct.itt ~., data = wtloss.5pct.itt2.1,

method = "rf",

ntree = 500,

trControl = control_boot,

tuneGrid = rfGrid,

preProcess = c("center", "scale"),

metric = "ROC")

wtloss.5pct.ittFit_boot_rf #bestTune mtry = 4, ROC = .59

CI(wtloss.5pct.ittFit_boot_rf$resample$ROC, ci = 0.95)

wtloss.5pct.ittFit_boot_rf.df <- as.data.frame(varImp(wtloss.5pct.ittFit_boot_rf$finalModel))

View(wtloss.5pct.ittFit_boot_rf.df)

set.seed(825)

wtloss.5pct.ittFit_opt_rf <- train(post.wtloss.5pct.itt ~., data = wtloss.5pct.itt2.1,

method = "rf",

ntree = 500,

trControl = control_opt,

tuneGrid = rfGrid,

preProcess = c("center", "scale"),

metric = "ROC")

wtloss.5pct.ittFit_opt_rf #bestTune mtry = 2, ROC = .94

View(wtloss.5pct.ittFit_opt_rf$results)

wtloss.5pct.ittFit_opt_rf_me <- qt(.975, 99)*(.06/sqrt(100))

.94 - wtloss.5pct.ittFit_opt_rf_me

.94+wtloss.5pct.ittFit_opt_rf_me

wtloss.5pct.ittFit_opt_rf.df <- as.data.frame(varImp(wtloss.5pct.ittFit_opt_rf$finalModel))

View(wtloss.5pct.ittFit_opt_rf.df)

#-------------------6. Linear: Continuous---------------

###set control functions for 10 repeated CV and standard bootstrapping

control.cont_cv <- trainControl(

method = "repeatedcv",

number = 10,

repeats = 10,

savePredictions = "final",

selectionFunction = "oneSE",

allowParallel = TRUE)

control.cont_boot <- trainControl(

##boot

method = "boot",

number = 100,

savePredictions = "final",

selectionFunction = "oneSE",

allowParallel = TRUE)

control.cont_opt <- trainControl(method = "optimism_boot",

number = 100,

savePredictions = "final",

selectionFunction = "oneSE",

allowParallel = TRUE)

#-----6a. OBE % reduction.itt----

set.seed(825)

obe.ittFit.l <- train(post.OBE_pct.itt ~., obe.pct.itt2.1,

method = "glm",

family = "gaussian",

preProcess = c("center", "scale"),

trControl = control.cont_cv)

obe.ittFit.l #R2 = .03, RMSE = 37.41

CI(obe.ittFit.l$resample$Rsquared, ci = 0.95)

CI(obe.ittFit.l$resample$RMSE, ci = 0.95)

obe.ittFit.l.df <- as.data.frame(obe.ittFit.l$finalModel$coefficients)

View(obe.ittFit.l.df)

set.seed(825)

obe.ittFit_boot.l <- train(post.OBE_pct.itt ~., data = obe.pct.itt2.1,

method = "glm",

family = "gaussian",

preProcess = c("center", "scale"),

trControl = control.cont_boot)

obe.ittFit_boot.l #R2 = .01, RMSE = 43.16

CI(obe.ittFit_boot.l$resample$Rsquared, ci = 0.95)

CI(obe.ittFit_boot.l$resample$RMSE, ci = 0.95)

obe.ittFit_boot.l.df <- as.data.frame(obe.ittFit_boot.l$finalModel$coefficients)

View(obe.ittFit_boot.l.df)

set.seed(825)

obe.ittFit_opt.l <- train(post.OBE_pct.itt ~., obe.pct.itt2.1,

method = "glm",

family = "gaussian",

preProcess = c("center", "scale"),

trControl = control.cont_opt)

obe.ittFit_opt.l #R2 = -.04, RMSE = 40.83

obe.ittFit_opt.l.r2_me <- qt(.975, 99)*(obe.ittFit_opt.l$results$RsquaredSD/sqrt(100))

mean(obe.ittFit_opt.l$results$Rsquared) - obe.ittFit_opt.l.r2_me

mean(obe.ittFit_opt.l$results$Rsquared)+obe.ittFit_opt.l.r2_me

obe.ittFit_opt.l.rm_me <- qt(.975, 99)*(obe.ittFit_opt.l$results$RMSESD/sqrt(100))

mean(obe.ittFit_opt.l$results$RMSE) - obe.ittFit_opt.l.rm_me

mean(obe.ittFit_opt.l$results$RMSE)+obe.ittFit_opt.l.rm_me

obe.ittFit_opt.l.df <- as.data.frame(obe.ittFit_opt.l$finalModel$coefficients)

View(obe.ittFit_opt.l.df)

#-----6b. EDE at post.itt----

set.seed(825)

ede.ittFit.l <- train(Total.EDEint.post.itt ~., data = ede.itt2.1,

method = "glm",

family = "gaussian",

preProcess = c("center", "scale"),

trControl = control.cont_cv)

ede.ittFit.l #R2 = .26, RMSE = .75

CI(ede.ittFit.l$resample$Rsquared, ci = 0.95)

CI(ede.ittFit.l$resample$RMSE, ci = 0.95)

ede.ittFit.l.df <- as.data.frame(ede.ittFit.l$finalModel$coefficients)

View(ede.ittFit.l.df)

set.seed(825)

ede.ittFit_boot.l <- train(Total.EDEint.post.itt ~., data = ede.itt2.1,

method = "glm",

family = "gaussian",

preProcess = c("center", "scale"),

trControl = control.cont_boot)

ede.ittFit_boot.l #R2 = .17, RMSE = .82

CI(ede.ittFit_boot.l$resample$Rsquared, ci = 0.95)

CI(ede.ittFit_boot.l$resample$RMSE, ci = 0.95)

ede.ittFit_boot.l.df <- as.data.frame(ede.ittFit_boot.l$finalModel$coefficients)

View(ede.ittFit_boot.l.df)

set.seed(825)

ede.ittFit_opt.l <- train(Total.EDEint.post.itt ~., data = ede.itt2.1,

method = "glm",

family = "gaussian",

preProcess = c("center", "scale"),

trControl = control.cont_opt)

ede.ittFit_opt.l #R2 = .23, RMSE = .75

ede.ittFit_opt.l.r2_me <- qt(.975, 99)*(ede.ittFit_opt.l$results$RsquaredSD/sqrt(100))

mean(ede.ittFit_opt.l$results$Rsquared) - ede.ittFit_opt.l.r2_me

mean(ede.ittFit_opt.l$results$Rsquared)+ede.ittFit_opt.l.r2_me

ede.ittFit_opt.l.rm_me <- qt(.975, 99)*(ede.ittFit_opt.l$results$RMSESD/sqrt(100))

mean(ede.ittFit_opt.l$results$RMSE) - ede.ittFit_opt.l.rm_me

mean(ede.ittFit_opt.l$results$RMSE)+ede.ittFit_opt.l.rm_me

ede.ittFit_opt.l.df <- as.data.frame(ede.ittFit_opt.l$finalModel$coefficients)

View(ede.ittFit_opt.l.df)

#-----6c. wt loss %.itt----

set.seed(825)

wtloss.ittFit.l <- train(post.wtloss_pct.itt ~., data = wtloss.pct.itt2.1,

method = "glm",

family = "gaussian",

preProcess = c("center", "scale"),

trControl = control.cont_cv)

wtloss.ittFit.l #R2 = .11, RMSE = 7.23

CI(wtloss.ittFit.l$resample$Rsquared, ci = 0.95)

CI(wtloss.ittFit.l$resample$RMSE, ci = 0.95)

wtloss.ittFit.l.df <- as.data.frame(wtloss.ittFit.l$finalModel$coefficients)

View(wtloss.ittFit.l.df)

set.seed(825)

wtloss.ittFit_boot.l <- train(post.wtloss_pct.itt ~., data = wtloss.pct.itt2.1,

method = "glm",

family = "gaussian",

preProcess = c("center", "scale"),

trControl = control.cont_boot)

wtloss.ittFit_boot.l #R2 = .06, RMSE = 7.83

CI(wtloss.ittFit_boot.l$resample$Rsquared, ci = 0.95)

CI(wtloss.ittFit_boot.l$resample$RMSE, ci = 0.95)

wtloss.ittFit_boot.l.df <- as.data.frame(wtloss.ittFit_boot.l$finalModel$coefficients)

View(wtloss.ittFit_boot.l.df)

set.seed(825)

wtloss.ittFit_opt.l <- train(post.wtloss_pct.itt ~., data = wtloss.pct.itt2.1,

method = "glm",

family = "gaussian",

preProcess = c("center", "scale"),

trControl = control.cont_opt)

wtloss.ittFit_opt.l #R2 = .07, RMSE = 7.24

wtloss.ittFit_opt.l.r2_me <- qt(.975, 99)*(wtloss.ittFit_opt.l$results$RsquaredSD/sqrt(100))

mean(wtloss.ittFit_opt.l$results$Rsquared) - wtloss.ittFit_opt.l.r2_me

mean(wtloss.ittFit_opt.l$results$Rsquared)+wtloss.ittFit_opt.l.r2_me

wtloss.ittFit_opt.l.rm_me <- qt(.975, 99)*(wtloss.ittFit_opt.l$results$RMSESD/sqrt(100))

mean(wtloss.ittFit_opt.l$results$RMSE) - wtloss.ittFit_opt.l.rm_me

mean(wtloss.ittFit_opt.l$results$RMSE)+wtloss.ittFit_opt.l.rm_me

wtloss.ittFit_opt.l.df <- as.data.frame(wtloss.ittFit_opt.l$finalModel$coefficients)

View(wtloss.ittFit_opt.l.df)

#---------------7. elastic nets: continuous----------------

#continuous: ITT

#centering outcomes for elastic nets

sc2 <- sc2 %>%

mutate(post.OBE_pct.itt = post.OBE_pct.itt - mean(post.OBE_pct.itt),

Total.EDEint.post.itt = Total.EDEint.post.itt - mean(Total.EDEint.post.itt),

post.wtloss_pct.itt = post.wtloss_pct.itt - mean(post.wtloss_pct.itt))

obe.pct.itt <- sc2 %>%

select(-c(remit.post.itt, remit.post.lnf.itt,

Total.EDEint.post.itt,

post.wtloss.5pct.itt, post.wtloss_pct.itt))

ede.itt <- sc2 %>%

select(-c(remit.post.itt, remit.post.lnf.itt,

post.OBE_pct.itt,

post.wtloss.5pct.itt, post.wtloss_pct.itt))

wtloss.pct.itt <- sc2 %>%

select(-c(remit.post.itt, remit.post.lnf.itt,

post.OBE_pct.itt, Total.EDEint.post.itt,

post.wtloss.5pct.itt))

obe.pct.itt <- as.data.frame(obe.pct.itt)

ede.itt <- as.data.frame(ede.itt)

wtloss.pct.itt <- as.data.frame(wtloss.pct.itt)

obe.pct.itt <- obe.pct.itt %>%

mutate(rapid = ifelse(rapid.itt == "nonrapid", 0, 1)) %>%

select(-rapid.itt)

ede.itt <- ede.itt %>%

mutate(rapid = ifelse(rapid.itt == "nonrapid", 0, 1)) %>%

select(-rapid.itt)

wtloss.pct.itt <- wtloss.pct.itt %>%

mutate(rapid = ifelse(rapid.itt == "nonrapid", 0, 1)) %>%

select(-rapid.itt)

#-----7a. OBE % reduction.itt----

set.seed(825)

obe.ittFit <- train(post.OBE_pct.itt ~., obe.pct.itt,

method = "glmnet",

family = "gaussian",

trControl = control.cont_cv,

preProcess = c("center", "scale"),

tuneGrid = enetGrid)

obe.ittFit$bestTune

View(obe.ittFit$results) #R2 = .03, RMSE = 37.10

CI(obe.ittFit$resample$Rsquared, ci = 0.95)

CI(obe.ittFit$resample$RMSE, ci = 0.95)

obe.ittFit_coef <- predict(obe.ittFit$finalModel, s = obe.ittFit$finalModel$lambdaOpt, type = "coefficients")

obe.ittFit_coef <- as.data.frame(as.matrix(obe.ittFit_coef))

set.seed(825)

obe.ittFit_boot <- train(post.OBE_pct.itt ~., data = obe.pct.itt,

method = "glmnet",

family = "gaussian",

trControl = control.cont_boot,

preProcess = c("center", "scale"),

tuneGrid = enetGrid)

obe.ittFit_boot$bestTune

View(obe.ittFit_boot$results) #R2 = .01, RMSE = 42.49

r2 <- obe.ittFit_boot$resample %>%

dplyr::filter(!is.na(obe.ittFit_boot$resample$Rsquared))

CI(r2$Rsquared, ci = 0.95)

CI(obe.ittFit_boot$resample$RMSE, ci = 0.95)

obe.ittFit_boot_coef <- predict(obe.ittFit_boot$finalModel, s = obe.ittFit_boot$finalModel$lambdaOpt, type = "coefficients")

obe.ittFit_boot_coef <- as.data.frame(as.matrix(obe.ittFit_boot_coef))

set.seed(825)

obe.ittFit_opt <- train(post.OBE_pct.itt ~., obe.pct.itt,

method = "glmnet",

family = "gaussian",

trControl = control.cont_opt,

preProcess = c("center", "scale"),

tuneGrid = enetGrid)

obe.ittFit_opt$bestTune

View(obe.ittFit_opt$results)

obe.ittFit_opt.r2_me <- qt(.975, 99)*(.01/sqrt(100))

.05 - obe.ittFit_opt.r2_me

.05+obe.ittFit_opt.r2_me

obe.ittFit_opt.rm_me <- qt(.975, 99)*(.22/sqrt(100))

0.48 - obe.ittFit_opt.rm_me

0.48+obe.ittFit_opt.rm_me

obe.ittFit_opt_coef <- predict(obe.ittFit_opt$finalModel, s = obe.ittFit_opt$finalModel$lambdaOpt, type = "coefficients")

obe.ittFit_opt_coef <- as.data.frame(as.matrix(obe.ittFit_opt_coef))

#-----7b. EDE at post.itt----

set.seed(825)

ede.ittFit <- train(Total.EDEint.post.itt ~., data = ede.itt,

method = "glmnet",

family = "gaussian",

trControl = control.cont_cv,

preProcess = c("center", "scale"),

tuneGrid = enetGrid)

ede.ittFit$bestTune

View(ede.ittFit$results) #R2 = .30, RMSE = .71

CI(ede.ittFit$resample$Rsquared, ci = 0.95)

CI(ede.ittFit$resample$RMSE, ci = 0.95)

ede.ittFit_coef <- predict(ede.ittFit$finalModel, s = ede.ittFit$finalModel$lambdaOpt, type = "coefficients")

ede.ittFit_coef <- as.data.frame(as.matrix(ede.ittFit_coef))

set.seed(825)

ede.ittFit_boot <- train(Total.EDEint.post.itt ~., data = ede.itt,

method = "glmnet",

family = "gaussian",

trControl = control.cont_boot,

preProcess = c("center", "scale"),

tuneGrid = enetGrid)

ede.ittFit_boot$bestTune

View(ede.ittFit_boot$results) #R2 = .25, RMSE = .74

CI(ede.ittFit_boot$resample$Rsquared, ci = 0.95)

CI(ede.ittFit_boot$resample$RMSE, ci = 0.95)

ede.ittFit_boot_coef <- predict(ede.ittFit_boot$finalModel, s = ede.ittFit_boot$finalModel$lambdaOpt, type = "coefficients")

ede.ittFit_boot_coef <- as.data.frame(as.matrix(ede.ittFit_boot_coef))

set.seed(825)

ede.ittFit_opt <- train(Total.EDEint.post.itt ~., data = ede.itt,

method = "glmnet",

family = "gaussian",

trControl = control.cont_opt,

preProcess = c("center", "scale"),

tuneGrid = enetGrid)

ede.ittFit_opt$bestTune

View(ede.ittFit_opt$results) #R2 = .30, RMSE = .71

ede.ittFit_opt.r2_me <- qt(.975, 99)*(.07/sqrt(100))

.23 - ede.ittFit_opt.r2_me

.23+ede.ittFit_opt.r2_me

ede.ittFit_opt.rm_me <- qt(.975, 99)*(.05/sqrt(100))

.74 - ede.ittFit_opt.rm_me

.74 +ede.ittFit_opt.rm_me

ede.ittFit_opt_coef <- predict(ede.ittFit_opt$finalModel, s = ede.ittFit_opt$finalModel$lambdaOpt, type = "coefficients")

ede.ittFit_opt_coef <- as.data.frame(as.matrix(ede.ittFit_opt_coef))

#-----7c. wt loss %.itt----

set.seed(825)

wtloss.ittFit <- train(post.wtloss_pct.itt ~., data = wtloss.pct.itt,

method = "glmnet",

family = "gaussian",

trControl = control.cont_cv,

preProcess = c("center", "scale"),

tuneGrid = enetGrid)

wtloss.ittFit$bestTune

View(wtloss.ittFit$results) #R2 = .11, RMSE = 7.18

CI(wtloss.ittFit$resample$Rsquared, ci = 0.95)

CI(wtloss.ittFit$resample$RMSE, ci = 0.95)

wtloss.ittFit_coef <- predict(wtloss.ittFit$finalModel, s = wtloss.ittFit$finalModel$lambdaOpt, type = "coefficients")

wtloss.ittFit_coef <- as.data.frame(as.matrix(wtloss.ittFit_coef))

set.seed(825)

wtloss.ittFit_boot <- train(post.wtloss_pct.itt ~., data = wtloss.pct.itt,

method = "glmnet",

family = "gaussian",

trControl = control.cont_boot,

preProcess = c("center", "scale"),

tuneGrid = enetGrid)

wtloss.ittFit_boot$bestTune

View(wtloss.ittFit_boot$results) #R2 = .05, RMSE = 7.57

CI(wtloss.ittFit_boot$resample$Rsquared, ci = 0.95)

CI(wtloss.ittFit_boot$resample$RMSE, ci = 0.95)

wtloss.ittFit_boot_coef <- predict(wtloss.ittFit_boot$finalModel, s = wtloss.ittFit_boot$finalModel$lambdaOpt, type = "coefficients")

wtloss.ittFit_boot_coef <- as.data.frame(as.matrix(wtloss.ittFit_boot_coef))

set.seed(825)

wtloss.ittFit_opt <- train(post.wtloss_pct.itt ~., data = wtloss.pct.itt,

method = "glmnet",

family = "gaussian",

trControl = control.cont_opt,

preProcess = c("center", "scale"),

tuneGrid = enetGrid)

wtloss.ittFit_opt$bestTune

View(wtloss.ittFit_opt$results) #R2 = .11, RMSE = 7.18

wtloss.ittFit_opt.r2_me <- qt(.975, 99)*(.05/sqrt(100))

.10 - wtloss.ittFit_opt.r2_me

.10+wtloss.ittFit_opt.r2_me

wtloss.ittFit_opt.rm_me <- qt(.975, 99)*(.65/sqrt(100))

7.08 - wtloss.ittFit_opt.rm_me

7.08 +wtloss.ittFit_opt.rm_me

wtloss.ittFit_opt_coef <- predict(wtloss.ittFit_opt$finalModel, s = wtloss.ittFit_opt$finalModel$lambdaOpt, type = "coefficients")

wtloss.ittFit_opt_coef <- as.data.frame(as.matrix(wtloss.ittFit_opt_coef))

#-----------------8. Random forests: continuous-----------------

#-----8a. OBE % reduction.itt----

set.seed(825)

obe.ittFit_rf <- train(post.OBE_pct.itt ~., obe.pct.itt2.1,

method = "rf",

ntree = 500,

trControl = control.cont_cv,

preProcess = c("center", "scale"),

tuneGrid = rfGrid)

obe.ittFit_rf #R2 = .07, RMSE = 33.22

CI(obe.ittFit_rf$resample$Rsquared, ci = 0.95)

CI(obe.ittFit_rf$resample$RMSE, ci = 0.95)

obe.ittFit_rf.df <- as.data.frame(varImp(obe.ittFit_rf$finalModel))

View(obe.ittFit_rf.df)

set.seed(825)

obe.ittFit_boot_rf <- train(post.OBE_pct.itt ~., data = obe.pct.itt2.1,

method = "rf",

ntree = 500,

trControl = control.cont_boot,

preProcess = c("center", "scale"),

tuneGrid = rfGrid)

obe.ittFit_boot_rf #R2 = .03, RMSE = 36.66

CI(obe.ittFit_boot_rf$resample$Rsquared, ci = 0.95)

CI(obe.ittFit_boot_rf$resample$RMSE, ci = 0.95)

obe.ittFit_boot_rf.df <- as.data.frame(varImp(obe.ittFit_boot_rf$finalModel))

View(obe.ittFit_boot_rf.df)

set.seed(825)

obe.ittFit_opt_rf <- train(post.OBE_pct.itt ~., data = obe.pct.itt2.1,

method = "rf",

ntree = 500,

trControl = control.cont_opt,

preProcess = c("center", "scale"),

tuneGrid = rfGrid)

obe.ittFit_opt_rf #R2 = .56, RMSE = 32.68

View(obe.ittFit_opt_rf$results)

obe.ittFit_opt_rf.r2_me <- qt(.975, 99)*(.01/sqrt(100))

.634 - obe.ittFit_opt_rf.r2_me

.634+obe.ittFit_opt_rf.r2_me

obe.ittFit_opt_rf.rm_me <- qt(.975, 99)*(.24/sqrt(100))

.37 - obe.ittFit_opt_rf.rm_me

.37+obe.ittFit_opt_rf.rm_me

obe.ittFit_opt_rf.df <- as.data.frame(varImp(obe.ittFit_opt_rf$finalModel))

View(obe.ittFit_opt_rf.df)

#-----8b. EDE at post.itt----

set.seed(825)

ede.ittFit_rf <- train(Total.EDEint.post.itt ~., data = ede.itt2.1,

method = "rf",

ntree = 500,

trControl = control.cont_cv,

preProcess = c("center", "scale"),

tuneGrid = rfGrid)

ede.ittFit_rf #R2 = .29, RMSE = .72

CI(ede.ittFit_rf$resample$Rsquared, ci = 0.95)

CI(ede.ittFit_rf$resample$RMSE, ci = 0.95)

ede.ittFit_rf.df <- as.data.frame(varImp(ede.ittFit_rf$finalModel))

View(ede.ittFit_rf.df)

set.seed(825)

ede.ittFit_boot_rf <- train(Total.EDEint.post.itt ~., data = ede.itt2.1,

method = "rf",

ntree = 500,

trControl = control.cont_boot,

preProcess = c("center", "scale"),

tuneGrid = rfGrid)

ede.ittFit_boot_rf #R2 = .25, RMSE = .74

CI(ede.ittFit_boot_rf$resample$Rsquared, ci = 0.95)

CI(ede.ittFit_boot_rf$resample$RMSE, ci = 0.95)

ede.ittFit_boot_rf.df <- as.data.frame(varImp(ede.ittFit_boot_rf$finalModel))

View(ede.ittFit_boot_rf.df)

set.seed(825)

ede.ittFit_opt_rf <- train(Total.EDEint.post.itt ~., data = ede.itt2.1,

method = "rf",

ntree = 500,

trControl = control.cont_opt,

preProcess = c("center", "scale"),

tuneGrid = rfGrid)

ede.ittFit_opt_rf #R2 = .64, RMSE = .63

ede.ittFit_opt_rf$results

obe.ittFit_opt_rf.r2_me <- qt(.975, 99)*(.07/sqrt(100))

.63 - obe.ittFit_opt_rf.r2_me

.63+obe.ittFit_opt_rf.r2_me

obe.ittFit_opt_rf.rm_me <- qt(.975, 99)*(.05/sqrt(100))

.61 - obe.ittFit_opt_rf.rm_me

.61 +obe.ittFit_opt_rf.rm_me

ede.ittFit_opt_rf.df <- as.data.frame(varImp(ede.ittFit_opt_rf$finalModel))

View(ede.ittFit_opt_rf.df)

#-----8c. wt loss %.itt----

set.seed(825)

wtloss.ittFit_rf <- train(post.wtloss_pct.itt ~., data = wtloss.pct.itt2.1,

method = "rf",

ntree = 500,

trControl = control.cont_cv,

preProcess = c("center", "scale"),

tuneGrid = rfGrid)

wtloss.ittFit_rf #R2 = .09, RMSE = 6.94

CI(wtloss.ittFit_rf$resample$Rsquared, ci = 0.95)

CI(wtloss.ittFit_rf$resample$RMSE, ci = 0.95)

wtloss.ittFit_rf.df <- as.data.frame(varImp(wtloss.ittFit_rf$finalModel))

View(wtloss.ittFit_rf.df)

set.seed(825)

wtloss.ittFit_boot_rf <- train(post.wtloss_pct.itt ~., data = wtloss.pct.itt2.1,

method = "rf",

ntree = 500,

trControl = control.cont_boot,

preProcess = c("center", "scale"),

tuneGrid = rfGrid)

wtloss.ittFit_boot_rf #R2 = .04, RMSE = 7.22

CI(wtloss.ittFit_boot_rf$resample$Rsquared, ci = 0.95)

CI(wtloss.ittFit_boot_rf$resample$RMSE, ci = 0.95)

wtloss.ittFit_boot_rf.df <- as.data.frame(varImp(wtloss.ittFit_boot_rf$finalModel))

View(wtloss.ittFit_boot_rf.df)

set.seed(825)

wtloss.ittFit_opt_rf <- train(post.wtloss_pct.itt ~., data = wtloss.pct.itt2.1,

method = "rf",

ntree = 500,

trControl = control.cont_opt,

preProcess = c("center", "scale"),

tuneGrid = rfGrid)

wtloss.ittFit_opt_rf #R2 = .61, RMSE = 6.08

wtloss.ittFit_opt_rf$results

wtloss.ittFit_opt_rf.r2_me <- qt(.975, 99)*(.02/sqrt(100))

.59 - wtloss.ittFit_opt_rf.r2_me

.59+wtloss.ittFit_opt_rf.r2_me

wtloss.ittFit_opt_rf.rm_me <- qt(.975, 99)*(.59/sqrt(100))

5.90 - wtloss.ittFit_opt_rf.rm_me

5.90+wtloss.ittFit_opt_rf.rm_me

wtloss.ittFit_opt_rf.df <- as.data.frame(varImp(wtloss.ittFit_opt_rf$finalModel))

View(wtloss.ittFit_opt_rf.df)

#-----------9. graphs------------

#variable importance figures

library(gridExtra)

#-----2. remit-----

imp.remit.l.10cv <- as.data.frame(varImp(remit.lnf.ittFit.l, scale = FALSE)$importance)

imp.remit.l.boot <- as.data.frame(varImp(remit.lnf.ittFit_boot.l, scale = FALSE)$importance)

imp.remit.l.opt <- as.data.frame(varImp(remit.lnf.ittFit_opt.l, scale = FALSE)$importance)

imp.remit.l <- bind_cols(imp.remit.l.10cv, imp.remit.l.boot, imp.remit.l.opt)

imp.remit.l$avg <- round(rowMeans(imp.remit.l), digits = 2)

imp.remit.l$variable <- c("Age", "Group", "Sex", "Race", "Edu", "Overvaluation", "Dissatisfy", "Restraint (EDE)", "Dep score", "BMI",

"Self-control", "Weight bias", "CR brood", "CR reflect", "Food add crit", "Food add cat", "Dep dx",

"Anx dx", "PTSD dx", "DUD dx", "AUD dx", "Emo overeat", "Mental health", "Physical health", "Self-esteem", "Interpers prob", "ER nonacpt",

"ER goals", "ER impulse", "ER aware", "ER strategies", "ER clarity", "Food tht supp", "Restraint (TFEQ)",

"Wt cycle", "Bx.ind rapid", "Bx.ind full", "Bx.ind alone", "OBE distress", "Diet hist", "Rapid",

"OBE")

imp.remit.l = imp.remit.l[,c(4,5)]

imp.remit.l <- imp.remit.l %>%

arrange(avg) %>%

tail(20)

imp.remit.l$number = seq.int(nrow(imp.remit.l))

View(imp.remit.enet)

imp.remit.enet.10cv <- as.data.frame(varImp(remit.lnf.ittFit, scale = FALSE)$importance)

imp.remit.enet.boot <- as.data.frame(varImp(remit.lnf.ittFit_boot, scale = FALSE)$importance)

imp.remit.enet.opt <- as.data.frame(varImp(remit.lnf.ittFit_opt, scale = FALSE)$importance)

imp.remit.enet <- bind_cols(imp.remit.enet.10cv, imp.remit.enet.boot, imp.remit.enet.opt)

imp.remit.enet$avg <- round(rowMeans(imp.remit.enet), digits = 2)

imp.remit.enet$variable <- c("Age", "Group", "Sex", "Race", "Edu", "Overvaluation", "Dissatisfy", "Restraint (EDE)", "Dep score", "BMI",

"Self-control", "Weight bias", "CR brood", "CR reflect", "Food add crit", "Food add cat", "Dep dx",

"Anx dx", "PTSD dx", "DUD dx", "AUD dx", "Emo overeat", "Mental health", "Physical health", "Self-esteem", "Interpers prob", "ER nonacpt",

"ER goals", "ER impulse", "ER aware", "ER strategies", "ER clarity", "Food tht supp", "Restraint (TFEQ)",

"Wt cycle", "Bx.ind rapid", "Bx.ind full", "Bx.ind alone", "OBE distress", "Diet hist", "OBE", "Rapid")

imp.remit.enet = imp.remit.enet[,c(4,5)]

imp.remit.enet <- imp.remit.enet %>%

arrange(avg) %>%

tail(20)

imp.remit.enet$number = seq.int(nrow(imp.remit.enet))

imp.remit.rf.10cv <- as.data.frame(varImp(remit.lnf.ittFit_rf, scale = FALSE)$importance)

imp.remit.rf.boot <- as.data.frame(varImp(remit.lnf.ittFit_boot_rf, scale = FALSE)$importance)

imp.remit.rf.opt <- as.data.frame(varImp(remit.lnf.ittFit_opt_rf, scale = FALSE)$importance)

imp.remit.rf <- bind_cols(imp.remit.rf.10cv, imp.remit.rf.boot, imp.remit.rf.opt)

imp.remit.rf$avg <- round(rowMeans(imp.remit.rf), digits = 2)

imp.remit.rf$variable <- c("Age", "Group", "Sex", "Race", "Edu", "Overvaluation", "Dissatisfy", "Restraint (EDE)", "Dep score", "BMI",

"Self-control", "Weight bias", "CR brood", "CR reflect", "Food add crit", "Food add cat", "Dep dx",

"Anx dx", "PTSD dx", "DUD dx", "AUD dx", "Emo overeat", "Mental health", "Physical health", "Self-esteem", "Interpers prob", "ER nonacpt",

"ER goals", "ER impulse", "ER aware", "ER strategies", "ER clarity", "Food tht supp", "Restraint (TFEQ)",

"Wt cycle", "Bx.ind rapid", "Bx.ind full", "Bx.ind alone", "OBE distress", "Diet hist", "Rapid",

"OBE")

imp.remit.rf = imp.remit.rf[,c(4,5)]

imp.remit.rf <- imp.remit.rf %>%

arrange(avg) %>%

tail(20)

imp.remit.rf$number = seq.int(nrow(imp.remit.rf))

View(imp.remit.rf)

fig2a <- ggplot2::ggplot(imp.remit.l) +

geom_segment(aes(x = number, y = 0, xend = number, yend = avg)) +

geom_point(aes(x = number, y = avg),

show.legend = F) +

scale_x_continuous(breaks=c(1:20), labels=imp.remit.l$variable)+

labs(title = "Logistic", y = "Importance", x = "")+

coord_flip() +

theme_bw()+

theme(plot.title = element_text(size=11))

fig2b <- ggplot2::ggplot(imp.remit.enet) +

geom_segment(aes(x = number, y = 0, xend = number, yend = avg)) +

geom_point(aes(x = number, y = avg),

show.legend = F) +

scale_x_continuous(breaks=c(1:20), labels=imp.remit.enet$variable)+

scale_y_continuous(breaks=c(0.00, 0.07, 0.15))+

labs(title = "Elastic net", y = "Importance", x = "")+

coord_flip() +

theme_bw()+

theme(plot.title = element_text(size=11))

fig2c <- ggplot2::ggplot(imp.remit.rf) +

geom_segment(aes(x = number, y = 0, xend = number, yend = avg)) +

geom_point(aes(x = number, y = avg),

show.legend = F) +

scale_x_continuous(breaks=c(1:20), labels=imp.remit.rf$variable)+

labs(title = "Random forest", y = "Importance", x = "")+

coord_flip() +

theme_bw()+

theme(plot.title = element_text(size=11))

pdf("fig_remit.pdf")

fig2 <- grid.arrange(fig2a, fig2b, fig2c, nrow = 1,

top = grid::textGrob("A. Binge-eating abstinence", x = 0, hjust = 0))

dev.off()

#-----3. OBE-----

imp.obe.l.10cv <- as.data.frame(varImp(obe.ittFit.l, scale = FALSE)$importance)

imp.obe.l.boot <- as.data.frame(varImp(obe.ittFit_boot.l, scale = FALSE)$importance)

imp.obe.l.opt <- as.data.frame(varImp(obe.ittFit_opt.l, scale = FALSE)$importance)

imp.obe.l <- bind_cols(imp.obe.l.10cv, imp.obe.l.boot, imp.obe.l.opt)

imp.obe.l$avg <- round(rowMeans(imp.obe.l), digits = 2)

imp.obe.l$variable <- c("Age", "Group", "Sex", "Race", "Edu", "Overvaluation", "Dissatisfy", "Restraint (EDE)", "Dep score", "BMI",

"Self-control", "Weight bias", "CR brood", "CR reflect", "Food add crit", "Food add cat", "Dep dx",

"Anx dx", "PTSD dx", "DUD dx", "AUD dx", "Emo overeat", "Mental health", "Physical health", "Self-esteem", "Interpers prob", "ER nonacpt",

"ER goals", "ER impulse", "ER aware", "ER strategies", "ER clarity", "Food tht supp", "Restraint (TFEQ)",

"Wt cycle", "Bx.ind rapid", "Bx.ind full", "Bx.ind alone", "OBE distress", "Diet hist", "Rapid",

"OBE")

imp.obe.l = imp.obe.l[,c(4,5)]

imp.obe.l <- imp.obe.l %>%

arrange(avg) %>%

tail(20)

imp.obe.l$number = seq.int(nrow(imp.obe.l))

imp.obe.enet.10cv <- as.data.frame(varImp(obe.ittFit, scale = FALSE)$importance)

imp.obe.enet.boot <- as.data.frame(varImp(obe.ittFit_boot, scale = FALSE)$importance)

imp.obe.enet.opt <- as.data.frame(varImp(obe.ittFit_opt, scale = FALSE)$importance)

imp.obe.enet <- bind_cols(imp.obe.enet.10cv, imp.obe.enet.boot, imp.obe.enet.opt)

imp.obe.enet$avg <- round(rowMeans(imp.obe.enet), digits = 2)

imp.obe.enet$variable <- c("Age", "Group", "Sex", "Race", "Edu", "Overvaluation", "Dissatisfy", "Restraint (EDE)", "Dep score", "BMI",

"Self-control", "Weight bias", "CR brood", "CR reflect", "Food add crit", "Food add cat", "Dep dx",

"Anx dx", "PTSD dx", "DUD dx", "AUD dx", "Emo overeat", "Mental health", "Physical health", "Self-esteem", "Interpers prob", "ER nonacpt",

"ER goals", "ER impulse", "ER aware", "ER strategies", "ER clarity", "Food tht supp", "Restraint (TFEQ)",

"Wt cycle", "Bx.ind rapid", "Bx.ind full", "Bx.ind alone", "OBE distress", "Diet hist", "OBE", "Rapid")

imp.obe.enet = imp.obe.enet[,c(4,5)]

imp.obe.enet <- imp.obe.enet %>%

arrange(avg) %>%

tail(20)

imp.obe.enet$number = seq.int(nrow(imp.obe.enet))

View(imp.obe.enet)

imp.obe.rf.10cv <- as.data.frame(varImp(obe.ittFit_rf, scale = FALSE)$importance)

imp.obe.rf.boot <- as.data.frame(varImp(obe.ittFit_boot_rf, scale = FALSE)$importance)

imp.obe.rf.opt <- as.data.frame(varImp(obe.ittFit_opt_rf, scale = FALSE)$importance)

imp.obe.rf <- bind_cols(imp.obe.rf.10cv, imp.obe.rf.boot, imp.obe.rf.opt)

imp.obe.rf$avg <- round(rowMeans(imp.obe.rf), digits = 2)

imp.obe.rf$variable <- c("Age", "Group", "Sex", "Race", "Edu", "Overvaluation", "Dissatisfy", "Restraint (EDE)", "Dep score", "BMI",

"Self-control", "Weight bias", "CR brood", "CR reflect", "Food add crit", "Food add cat", "Dep dx",

"Anx dx", "PTSD dx", "DUD dx", "AUD dx", "Emo overeat", "Mental health", "Physical health", "Self-esteem", "Interpers prob", "ER nonacpt",

"ER goals", "ER impulse", "ER aware", "ER strategies", "ER clarity", "Food tht supp", "Restraint (TFEQ)",

"Wt cycle", "Bx.ind rapid", "Bx.ind full", "Bx.ind alone", "OBE distress", "Diet hist", "Rapid",

"OBE")

imp.obe.rf = imp.obe.rf[,c(4,5)]

imp.obe.rf <- imp.obe.rf %>%

arrange(avg) %>%

tail(20)

imp.obe.rf$number = seq.int(nrow(imp.obe.rf))

View(imp.obe.rf)

fig3a <- ggplot2::ggplot(imp.obe.l) +

geom_segment(aes(x = number, y = 0, xend = number, yend = avg)) +

geom_point(aes(x = number, y = avg),

show.legend = F) +

scale_x_continuous(breaks=c(1:20), labels=imp.obe.l$variable)+

labs(title = "Linear", y = "Importance", x = "")+

coord_flip() +

theme_bw()+

theme(plot.title = element_text(size=11))

fig3b <- ggplot2::ggplot(imp.obe.enet) +

geom_segment(aes(x = number, y = 0, xend = number, yend = avg)) +

geom_point(aes(x = number, y = avg),

show.legend = F) +

scale_x_continuous(breaks=c(1:20), labels=imp.obe.enet$variable)+

scale_y_continuous(breaks=c(0.01, 0.03, 0.05))+

labs(title = "Elastic net", y = "Importance", x = "")+

coord_flip() +

theme_bw()+

theme(plot.title = element_text(size=11))

fig3c <- ggplot2::ggplot(imp.obe.rf) +

geom_segment(aes(x = number, y = 0, xend = number, yend = avg)) +

geom_point(aes(x = number, y = avg),

show.legend = F) +

scale_x_continuous(breaks=c(1:20), labels=imp.obe.rf$variable)+

labs(title = "Random forest", y = "Importance", x = "")+

coord_flip() +

theme_bw()+

theme(plot.title = element_text(size=11))

pdf("fig_obe.pdf")

fig3 <- grid.arrange(fig3a, fig3b, fig3c, nrow = 1,

top = grid::textGrob("B. Binge eating reduction (%)", x = 0, hjust = 0))

dev.off()

#-----4. EDE-----

imp.ede.l.10cv <- as.data.frame(varImp(ede.ittFit.l, scale = FALSE)$importance)

imp.ede.l.boot <- as.data.frame(varImp(ede.ittFit_boot.l, scale = FALSE)$importance)

imp.ede.l.opt <- as.data.frame(varImp(ede.ittFit_opt.l, scale = FALSE)$importance)

imp.ede.l <- bind_cols(imp.ede.l.10cv, imp.ede.l.boot, imp.ede.l.opt)

imp.ede.l$avg <- round(rowMeans(imp.ede.l), digits = 2)

imp.ede.l$variable <- c("Age", "Group", "Sex", "Race", "Edu", "Overvaluation", "Dissatisfy", "Restraint (EDE)", "Dep score", "BMI",

"Self-control", "Weight bias", "CR brood", "CR reflect", "Food add crit", "Food add cat", "Dep dx",

"Anx dx", "PTSD dx", "DUD dx", "AUD dx", "Emo overeat", "Mental health", "Physical health", "Self-esteem", "Interpers prob", "ER nonacpt",

"ER goals", "ER impulse", "ER aware", "ER strategies", "ER clarity", "Food tht supp", "Restraint (TFEQ)",

"Wt cycle", "Bx.ind rapid", "Bx.ind full", "Bx.ind alone", "OBE distress", "Diet hist", "Rapid",

"OBE")

imp.ede.l = imp.ede.l[,c(4,5)]

imp.ede.l <- imp.ede.l %>%

arrange(avg) %>%

tail(20)

imp.ede.l$number = seq.int(nrow(imp.ede.l))

imp.ede.enet.10cv <- as.data.frame(varImp(ede.ittFit, scale = FALSE)$importance)

imp.ede.enet.boot <- as.data.frame(varImp(ede.ittFit_boot, scale = FALSE)$importance)

imp.ede.enet.opt <- as.data.frame(varImp(ede.ittFit_opt, scale = FALSE)$importance)

imp.ede.enet <- bind_cols(imp.ede.enet.10cv, imp.ede.enet.boot, imp.ede.enet.opt)

imp.ede.enet$avg <- round(rowMeans(imp.ede.enet), digits = 2)

imp.ede.enet$variable <- c("Age", "Group", "Sex", "Race", "Edu", "Overvaluation", "Dissatisfy", "Restraint (EDE)", "Dep score", "BMI",

"Self-control", "Weight bias", "CR brood", "CR reflect", "Food add crit", "Food add cat", "Dep dx",

"Anx dx", "PTSD dx", "DUD dx", "AUD dx", "Emo overeat", "Mental health", "Physical health", "Self-esteem", "Interpers prob", "ER nonacpt",

"ER goals", "ER impulse", "ER aware", "ER strategies", "ER clarity", "Food tht supp", "Restraint (TFEQ)",

"Wt cycle", "Bx.ind rapid", "Bx.ind full", "Bx.ind alone", "OBE distress", "Diet hist", "OBE", "Rapid")

imp.ede.enet = imp.ede.enet[,c(4,5)]

imp.ede.enet <- imp.ede.enet %>%

arrange(avg) %>%

tail(20)

imp.ede.enet$number = seq.int(nrow(imp.ede.enet))

View(imp.ede.enet)

imp.ede.rf.10cv <- as.data.frame(varImp(ede.ittFit_rf, scale = FALSE)$importance)

imp.ede.rf.boot <- as.data.frame(varImp(ede.ittFit_boot_rf, scale = FALSE)$importance)

imp.ede.rf.opt <- as.data.frame(varImp(ede.ittFit_opt_rf, scale = FALSE)$importance)

imp.ede.rf <- bind_cols(imp.ede.rf.10cv, imp.ede.rf.boot, imp.ede.rf.opt)

imp.ede.rf$avg <- round(rowMeans(imp.ede.rf), digits = 2)

imp.ede.rf$variable <- c("Age", "Group", "Sex", "Race", "Edu", "Overvaluation", "Dissatisfy", "Restraint (EDE)", "Dep score", "BMI",

"Self-control", "Weight bias", "CR brood", "CR reflect", "Food add crit", "Food add cat", "Dep dx",

"Anx dx", "PTSD dx", "DUD dx", "AUD dx", "Emo overeat", "Mental health", "Physical health", "Self-esteem", "Interpers prob", "ER nonacpt",

"ER goals", "ER impulse", "ER aware", "ER strategies", "ER clarity", "Food tht supp", "Restraint (TFEQ)",

"Wt cycle", "Bx.ind rapid", "Bx.ind full", "Bx.ind alone", "OBE distress", "Diet hist", "Rapid",

"OBE")

imp.ede.rf = imp.ede.rf[,c(4,5)]

imp.ede.rf <- imp.ede.rf %>%

arrange(avg) %>%

tail(20)

imp.ede.rf$number = seq.int(nrow(imp.ede.rf))

View(imp.ede.rf)

fig4a <- ggplot2::ggplot(imp.ede.l) +

geom_segment(aes(x = number, y = 0, xend = number, yend = avg)) +

geom_point(aes(x = number, y = avg),

show.legend = F) +

scale_x_continuous(breaks=c(1:20), labels=imp.ede.l$variable)+

labs(title = "Linear", y = "Importance", x = "")+

coord_flip() +

theme_bw()+

theme(plot.title = element_text(size=11))

fig4b <- ggplot2::ggplot(imp.ede.enet) +

geom_segment(aes(x = number, y = 0, xend = number, yend = avg)) +

geom_point(aes(x = number, y = avg),

show.legend = F) +

scale_x_continuous(breaks=c(1:20), labels=imp.ede.enet$variable)+

labs(title = "Elastic net", y = "Importance", x = "")+

coord_flip() +

theme_bw()+

theme(plot.title = element_text(size=11))

fig4c <- ggplot2::ggplot(imp.ede.rf) +

geom_segment(aes(x = number, y = 0, xend = number, yend = avg)) +

geom_point(aes(x = number, y = avg),

show.legend = F) +

scale_x_continuous(breaks=c(1:20), labels=imp.ede.rf$variable)+

labs(title = "Random forest", y = "Importance", x = "")+

coord_flip() +

theme_bw()+

theme(plot.title = element_text(size=11))

pdf("fig_ede.pdf")

fig4 <- grid.arrange(fig4a, fig4b, fig4c, nrow = 1,

top = grid::textGrob("Eating-disorder psychopathology", x = 0, hjust = 0))

dev.off()

#-----5. wtloss 5pct-----

imp.wtloss.5pct.l.10cv <- as.data.frame(varImp(wtloss.5pct.ittFit.l, scale = FALSE)$importance)

imp.wtloss.5pct.l.boot <- as.data.frame(varImp(wtloss.5pct.ittFit_boot.l, scale = FALSE)$importance)

imp.wtloss.5pct.l.opt <- as.data.frame(varImp(wtloss.5pct.ittFit_opt.l, scale = FALSE)$importance)

imp.wtloss.5pct.l <- bind_cols(imp.wtloss.5pct.l.10cv, imp.wtloss.5pct.l.boot, imp.wtloss.5pct.l.opt)

imp.wtloss.5pct.l$avg <- round(rowMeans(imp.wtloss.5pct.l), digits = 2)

imp.wtloss.5pct.l$variable <- c("Age", "Group", "Sex", "Race", "Edu", "Overvaluation", "Dissatisfy", "Restraint (EDE)", "Dep score", "BMI",

"Self-control", "Weight bias", "CR brood", "CR reflect", "Food add crit", "Food add cat", "Dep dx",

"Anx dx", "PTSD dx", "DUD dx", "AUD dx", "Emo overeat", "Mental health", "Physical health", "Self-esteem", "Interpers prob", "ER nonacpt",

"ER goals", "ER impulse", "ER aware", "ER strategies", "ER clarity", "Food tht supp", "Restraint (TFEQ)",

"Wt cycle", "Bx.ind rapid", "Bx.ind full", "Bx.ind alone", "OBE distress", "Diet hist", "Rapid",

"OBE")

imp.wtloss.5pct.l = imp.wtloss.5pct.l[,c(4,5)]

imp.wtloss.5pct.l <- imp.wtloss.5pct.l %>%

arrange(avg) %>%

tail(20)

imp.wtloss.5pct.l$number = seq.int(nrow(imp.wtloss.5pct.l))

imp.wtloss.5pct.enet.10cv <- as.data.frame(varImp(wtloss.5pct.ittFit, scale = FALSE)$importance)

imp.wtloss.5pct.enet.boot <- as.data.frame(varImp(wtloss.5pct.ittFit_boot, scale = FALSE)$importance)

imp.wtloss.5pct.enet.opt <- as.data.frame(varImp(wtloss.5pct.ittFit_opt, scale = FALSE)$importance)

imp.wtloss.5pct.enet <- bind_cols(imp.wtloss.5pct.enet.10cv, imp.wtloss.5pct.enet.boot, imp.wtloss.5pct.enet.opt)

imp.wtloss.5pct.enet$avg <- round(rowMeans(imp.wtloss.5pct.enet), digits = 2)

imp.wtloss.5pct.enet$variable <- c("Age", "Group", "Sex", "Race", "Edu", "Overvaluation", "Dissatisfy", "Restraint (EDE)", "Dep score", "BMI",

"Self-control", "Weight bias", "CR brood", "CR reflect", "Food add crit", "Food add cat", "Dep dx",

"Anx dx", "PTSD dx", "DUD dx", "AUD dx", "Emo overeat", "Mental health", "Physical health", "Self-esteem", "Interpers prob", "ER nonacpt",

"ER goals", "ER impulse", "ER aware", "ER strategies", "ER clarity", "Food tht supp", "Restraint (TFEQ)",

"Wt cycle", "Bx.ind rapid", "Bx.ind full", "Bx.ind alone", "OBE distress", "Diet hist", "OBE", "Rapid")

imp.wtloss.5pct.enet = imp.wtloss.5pct.enet[,c(4,5)]

imp.wtloss.5pct.enet <- imp.wtloss.5pct.enet %>%

arrange(avg) %>%

tail(20)

imp.wtloss.5pct.enet$number = seq.int(nrow(imp.wtloss.5pct.enet))

View(imp.wtloss.5pct.enet)

imp.wtloss.5pct.rf.10cv <- as.data.frame(varImp(wtloss.5pct.ittFit_rf, scale = FALSE)$importance)

imp.wtloss.5pct.rf.boot <- as.data.frame(varImp(wtloss.5pct.ittFit_boot_rf, scale = FALSE)$importance)

imp.wtloss.5pct.rf.opt <- as.data.frame(varImp(wtloss.5pct.ittFit_opt_rf, scale = FALSE)$importance)

imp.wtloss.5pct.rf <- bind_cols(imp.wtloss.5pct.rf.10cv, imp.wtloss.5pct.rf.boot, imp.wtloss.5pct.rf.opt)

imp.wtloss.5pct.rf$avg <- round(rowMeans(imp.wtloss.5pct.rf), digits = 2)

imp.wtloss.5pct.rf$variable <- c("Age", "Group", "Sex", "Race", "Edu", "Overvaluation", "Dissatisfy", "Restraint (EDE)", "Dep score", "BMI",

"Self-control", "Weight bias", "CR brood", "CR reflect", "Food add crit", "Food add cat", "Dep dx",

"Anx dx", "PTSD dx", "DUD dx", "AUD dx", "Emo overeat", "Mental health", "Physical health", "Self-esteem", "Interpers prob", "ER nonacpt",

"ER goals", "ER impulse", "ER aware", "ER strategies", "ER clarity", "Food tht supp", "Restraint (TFEQ)",

"Wt cycle", "Bx.ind rapid", "Bx.ind full", "Bx.ind alone", "OBE distress", "Diet hist", "Rapid",

"OBE")

imp.wtloss.5pct.rf = imp.wtloss.5pct.rf[,c(4,5)]

imp.wtloss.5pct.rf <- imp.wtloss.5pct.rf %>%

arrange(avg) %>%

tail(20)

imp.wtloss.5pct.rf$number = seq.int(nrow(imp.wtloss.5pct.rf))

View(imp.wtloss.5pct.rf)

fig5a <- ggplot2::ggplot(imp.wtloss.5pct.l) +

geom_segment(aes(x = number, y = 0, xend = number, yend = avg)) +

geom_point(aes(x = number, y = avg),

show.legend = F) +

scale_x_continuous(breaks=c(1:20), labels=imp.wtloss.5pct.l$variable)+

labs(title = "Logistic", y = "Importance", x = "")+

coord_flip() +

theme_bw()+

theme(plot.title = element_text(size=11))

fig5b <- ggplot2::ggplot(imp.wtloss.5pct.enet) +

geom_segment(aes(x = number, y = 0, xend = number, yend = avg)) +

geom_point(aes(x = number, y = avg),

show.legend = F) +

scale_x_continuous(breaks=c(1:20), labels=imp.wtloss.5pct.enet$variable)+

labs(title = "Elastic net", y = "Importance", x = "")+

coord_flip() +

theme_bw()+

theme(plot.title = element_text(size=11))

fig5c <- ggplot2::ggplot(imp.wtloss.5pct.rf) +

geom_segment(aes(x = number, y = 0, xend = number, yend = avg)) +

geom_point(aes(x = number, y = avg),

show.legend = F) +

scale_x_continuous(breaks=c(1:20), labels=imp.wtloss.5pct.rf$variable)+

labs(title = "Random forest", y = "Importance", x = "")+

coord_flip() +

theme_bw()+

theme(plot.title = element_text(size=11))

pdf("fig_wtloss.5pct.pdf")

fig5 <- grid.arrange(fig5a, fig5b, fig5c, nrow = 1,

top = grid::textGrob("A. Weight reduction >= 5%", x = 0, hjust = 0))

dev.off()

#-----6. wtloss %-----

imp.wtloss.l.10cv <- as.data.frame(varImp(wtloss.ittFit.l, scale = FALSE)$importance)

imp.wtloss.l.boot <- as.data.frame(varImp(wtloss.ittFit_boot.l, scale = FALSE)$importance)

imp.wtloss.l.opt <- as.data.frame(varImp(wtloss.ittFit_opt.l, scale = FALSE)$importance)

imp.wtloss.l <- bind_cols(imp.wtloss.l.10cv, imp.wtloss.l.boot, imp.wtloss.l.opt)

imp.wtloss.l$avg <- round(rowMeans(imp.wtloss.l), digits = 2)

imp.wtloss.l$variable <- c("Age", "Group", "Sex", "Race", "Edu", "Overvaluation", "Dissatisfy", "Restraint (EDE)", "Dep score", "BMI",

"Self-control", "Weight bias", "CR brood", "CR reflect", "Food add crit", "Food add cat", "Dep dx",

"Anx dx", "PTSD dx", "DUD dx", "AUD dx", "Emo overeat", "Mental health", "Physical health", "Self-esteem", "Interpers prob", "ER nonacpt",

"ER goals", "ER impulse", "ER aware", "ER strategies", "ER clarity", "Food tht supp", "Restraint (TFEQ)",

"Wt cycle", "Bx.ind rapid", "Bx.ind full", "Bx.ind alone", "OBE distress", "Diet hist", "Rapid",

"OBE")

imp.wtloss.l = imp.wtloss.l[,c(4,5)]

imp.wtloss.l <- imp.wtloss.l %>%

arrange(avg) %>%

tail(20)

imp.wtloss.l$number = seq.int(nrow(imp.wtloss.l))

imp.wtloss.enet.10cv <- as.data.frame(varImp(wtloss.ittFit, scale = FALSE)$importance)

imp.wtloss.enet.boot <- as.data.frame(varImp(wtloss.ittFit_boot, scale = FALSE)$importance)

imp.wtloss.enet.opt <- as.data.frame(varImp(wtloss.ittFit_opt, scale = FALSE)$importance)

imp.wtloss.enet <- bind_cols(imp.wtloss.enet.10cv, imp.wtloss.enet.boot, imp.wtloss.enet.opt)

imp.wtloss.enet$avg <- round(rowMeans(imp.wtloss.enet), digits = 2)

imp.wtloss.enet$variable <- c("Age", "Group", "Sex", "Race", "Edu", "Overvaluation", "Dissatisfy", "Restraint (EDE)", "Dep score", "BMI",

"Self-control", "Weight bias", "CR brood", "CR reflect", "Food add crit", "Food add cat", "Dep dx",

"Anx dx", "PTSD dx", "DUD dx", "AUD dx", "Emo overeat", "Mental health", "Physical health", "Self-esteem", "Interpers prob", "ER nonacpt",

"ER goals", "ER impulse", "ER aware", "ER strategies", "ER clarity", "Food tht supp", "Restraint (TFEQ)",

"Wt cycle", "Bx.ind rapid", "Bx.ind full", "Bx.ind alone", "OBE distress", "Diet hist", "OBE", "Rapid")

imp.wtloss.enet = imp.wtloss.enet[,c(4,5)]

imp.wtloss.enet <- imp.wtloss.enet %>%

arrange(avg) %>%

tail(20)

imp.wtloss.enet$number = seq.int(nrow(imp.wtloss.enet))

View(imp.wtloss.enet)

imp.wtloss.rf.10cv <- as.data.frame(varImp(wtloss.ittFit_rf, scale = FALSE)$importance)

imp.wtloss.rf.boot <- as.data.frame(varImp(wtloss.ittFit_boot_rf, scale = FALSE)$importance)

imp.wtloss.rf.opt <- as.data.frame(varImp(wtloss.ittFit_opt_rf, scale = FALSE)$importance)

imp.wtloss.rf <- bind_cols(imp.wtloss.rf.10cv, imp.wtloss.rf.boot, imp.wtloss.rf.opt)

imp.wtloss.rf$avg <- round(rowMeans(imp.wtloss.rf), digits = 2)

imp.wtloss.rf$variable <- c("Age", "Group", "Sex", "Race", "Edu", "Overvaluation", "Dissatisfy", "Restraint (EDE)", "Dep score", "BMI",

"Self-control", "Weight bias", "CR brood", "CR reflect", "Food add crit", "Food add cat", "Dep dx",

"Anx dx", "PTSD dx", "DUD dx", "AUD dx", "Emo overeat", "Mental health", "Physical health", "Self-esteem", "Interpers prob", "ER nonacpt",

"ER goals", "ER impulse", "ER aware", "ER strategies", "ER clarity", "Food tht supp", "Restraint (TFEQ)",

"Wt cycle", "Bx.ind rapid", "Bx.ind full", "Bx.ind alone", "OBE distress", "Diet hist", "Rapid",

"OBE")

imp.wtloss.rf = imp.wtloss.rf[,c(4,5)]

imp.wtloss.rf <- imp.wtloss.rf %>%

arrange(avg) %>%

tail(20)

imp.wtloss.rf$number = seq.int(nrow(imp.wtloss.rf))

View(imp.wtloss.rf)

fig6a <- ggplot2::ggplot(imp.wtloss.l) +

geom_segment(aes(x = number, y = 0, xend = number, yend = avg)) +

geom_point(aes(x = number, y = avg),

show.legend = F) +

scale_x_continuous(breaks=c(1:20), labels=imp.wtloss.l$variable)+

labs(title = "Linear", y = "Importance", x = "")+

coord_flip() +

theme_bw()+

theme(plot.title = element_text(size=11))

fig6b <- ggplot2::ggplot(imp.wtloss.enet) +

geom_segment(aes(x = number, y = 0, xend = number, yend = avg)) +

geom_point(aes(x = number, y = avg),

show.legend = F) +

scale_x_continuous(breaks=c(1:20), labels=imp.wtloss.enet$variable)+

labs(title = "Elastic net", y = "Importance", x = "")+

coord_flip() +

theme_bw()+

theme(plot.title = element_text(size=11))

fig6c <- ggplot2::ggplot(imp.wtloss.rf) +

geom_segment(aes(x = number, y = 0, xend = number, yend = avg)) +

geom_point(aes(x = number, y = avg),

show.legend = F) +

scale_x_continuous(breaks=c(1:20), labels=imp.wtloss.rf$variable)+

labs(title = "Random forest", y = "Importance", x = "")+

coord_flip() +

theme_bw()+

theme(plot.title = element_text(size=11))

pdf("fig_wtloss.pdf")

fig6 <- grid.arrange(fig6a, fig6b, fig6c, nrow = 1,

top = grid::textGrob("B. Weight reduction (%)", x = 0, hjust = 0))

dev.off()

pdf("figs1ab.pdf", height = 10)

grid.arrange(fig2, fig3, nrow = 2)

dev.off()

pdf("figs2ab.pdf", height = 5)

grid.arrange(fig4)

dev.off()

pdf("figs3ab.pdf", height = 10)

grid.arrange(fig5, fig6, nrow = 2)

dev.off()

#-------table 1-------

table(sc2.1$sex)

table(sc2.1$race)

table(sc$Ethnicity)

table(sc2.1$edu)

mean(sc2.1$Age)

sd(sc2.1$Age)

mean(sc2.1$base.bmi)

sd(sc2.1$base.bmi)

table(sc2.1$dep_any)

table(sc2.1$anx_any)

table(sc2.1$ptsd)

table(sc2.1$sud_any)

table(sc2.1$aud)

mean(sc$OBE.EDEInt)

sd(sc$OBE.EDEInt)

mean(sc2.1$ede_ov)

sd(sc2.1$ede_ov)

mean(sc2.1$ede_bd)

sd(sc2.1$ede_bd)

mean(sc2.1$ede_rest)

sd(sc2.1$ede_rest)

mean(sc2.1$TFEQ_rest)

sd(sc2.1$TFEQ_rest)

table(sc2.1$bxind_rapid)

table(sc2.1$bxind_full)

table(sc2.1$bxind_alone)

mean(sc2.1$obe_distress)

sd(sc2.1$obe_distress)

mean(sc2.1$wtcycl)

sd(sc2.1$wtcycl)

mean(sc2.1$diethx)

sd(sc2.1$diethx)

mean(sc2.1$EOQ_tot)

sd(sc2.1$EOQ_tot)

mean(sc2.1$FTSI_tot)

sd(sc2.1$FTSI_tot)

table(sc2.1$yfas_dx)

mean(sc2$yfas_crit)

sd(sc2$yfas_crit)

mean(sc2.1$DERS_nonaccept)

sd(sc2.1$DERS_nonaccept)

mean(sc2.1$DERS_goals)

sd(sc2.1$DERS_goals)

mean(sc2.1$DERS_impulse)

sd(sc2.1$DERS_impulse)

mean(sc2.1$DERS_aware)

sd(sc2.1$DERS_aware)

mean(sc2.1$DERS_strat)

sd(sc2.1$DERS_strat)

mean(sc2.1$DERS_clar)

sd(sc2.1$DERS_clar)

mean(sc2$scs_tot)

sd(sc2$scs_tot)

mean(sc2$wbis_tot)

sd(sc2$wbis_tot)

mean(sc2$bdi_tot)

sd(sc2$bdi_tot)

mean(sc2.1$RSES_tot)

sd(sc2.1$RSES_tot)

mean(sc2.1$IIP_tot)

sd(sc2.1$IIP_tot)

mean(sc2$rs_brood)

sd(sc2$rs_brood)

mean(sc2$rs_ponder)

sd(sc2$rs_ponder)

mean(sc2.1$sf_mh)

sd(sc2.1$sf_mh)

mean(sc2.1$sf_ph)

sd(sc2.1$sf_ph)

table(sc2.1$group)

table(sc2.1$rapid.itt)

table(sc2.1$remit.post.itt)

table(sc2.1$remit.post.lnf.itt)

mean(sc2.1$post.OBE_pct.itt)

sd(sc2.1$post.OBE_pct.itt)

mean(sc2.1$Total.EDEint.post.itt)

sd(sc2.1$Total.EDEint.post.itt)

table(sc2.1$post.wtloss.5pct.itt)

mean(sc2.1$post.wtloss_pct.itt)

sd(sc2.1$post.wtloss_pct.itt)

#-------------------10. carefully selected traditional regression----------------------

sc3 <- sc %>%

select(Sex, race, base.bmi, OBE.EDEInt, ede_ov, ede_8tot, EDE_tot, comorbid_any,

bdi_tot, Group, rapid,

remit.post.lnf.itt, post.OBE_pct.itt, Total.EDEint.post.itt,

post.wtloss.5pct.itt, post.wtloss_pct.itt)

sc3$Group <- factor(sc3$Group, levels = c(0, 1))

sc3$Sex <- factor(sc3$Sex, levels = c(0, 1))

sc3$race <- factor(sc3$race, levels = c(0, 1))

sc3$comorbid_any <- factor(sc3$comorbid_any, levels = c(0, 1))

sc3$remit.post.lnf.itt <- factor(sc3$remit.post.lnf.itt,

labels = c("nonremit", "remit"))

sc3$post.wtloss.5pct.itt <- factor(sc3$post.wtloss.5pct.itt,

labels = c("no", "yes"))

sc3$rapid <- factor(sc3$rapid, labels = c("nonrapid", "rapid"))

sc3$Group <- factor(sc3$Group, levels = c(0, 1),

labels= c("standard", "stepped"))

sc3$Sex <- factor(sc3$Sex, levels = c(0, 1),

labels = c("woman", "man"))

sc3$race <- factor(sc3$race, levels = c(0, 1),

labels = c("Non-white", "White"))

sc3$OBE.EDEInt2 <- log(sc3$OBE.EDEInt)

sc3$OBE.EDEInt <- NULL

sc3$post.OBE_pct.itt <- log(sc3$post.OBE_pct.itt + 201)

#---------10a. impute ---------

imp2 <- mice(sc3, m=1, maxit = 50, method = 'pmm', seed = 500)

summary(imp2)

sc3 <- complete(imp2,1)

#---------10b. remit ----------

set.seed(825)

csl_remit.cv <- train(remit.post.lnf.itt ~ Sex+ race+ base.bmi+ OBE.EDEInt2+ ede_ov+ ede_8tot+ comorbid_any+

bdi_tot+ Group+ rapid, data = sc3,

method = "glm",

family = "binomial",

trControl = control_cv,

preProcess = c("center", "scale"),

metric = "ROC")

csl_remit.cv

CI(csl_remit.cv$resample$ROC, ci = 0.95)

set.seed(825)

csl_remit.boot <- train(remit.post.lnf.itt ~ Sex+ race+ base.bmi+ OBE.EDEInt2+ ede_ov+ ede_8tot+ comorbid_any+

bdi_tot+ Group+ rapid, data = sc3,

method = "glm",

family = "binomial",

trControl = control_boot,

preProcess = c("center", "scale"),

metric = "ROC")

csl_remit.boot

CI(csl_remit.boot$resample$ROC, ci = 0.95)

set.seed(825)

csl_remit.opt <- train(remit.post.lnf.itt ~ Sex+ race+ base.bmi+ OBE.EDEInt2+ ede_ov+ ede_8tot+ comorbid_any+

bdi_tot+ Group+ rapid, data = sc3,

method = "glm",

family = "binomial",

trControl = control_opt,

preProcess = c("center", "scale"),

metric = "ROC")

csl_remit.opt

csl_remit.opt_me <- qt(.975, 99)*(csl_remit.opt$results$ROCSD/sqrt(100))

mean(csl_remit.opt$results$ROC) - csl_remit.opt_me

mean(csl_remit.opt$results$ROC)+csl_remit.opt_me

#-----10c. wtloss.5pct.itt----

set.seed(825)

csl_wtloss.5pct.cv <- train(post.wtloss.5pct.itt ~ Sex+ race+ base.bmi+ OBE.EDEInt2+ ede_ov+ ede_8tot+comorbid_any+

bdi_tot+ Group+ rapid, data = sc3,

method = "glm",

family = "binomial",

trControl = control_cv,

preProcess = c("center", "scale"),

metric = "ROC")

csl_wtloss.5pct.cv

CI(csl_wtloss.5pct.cv$resample$ROC, ci = 0.95)

set.seed(825)

csl_wtloss.5pct.boot <- train(post.wtloss.5pct.itt ~ Sex+ race+ base.bmi+ OBE.EDEInt2+ ede_ov+ ede_8tot+ comorbid_any+

bdi_tot+ Group+ rapid, data = sc3,

method = "glm",

family = "binomial",

trControl = control_boot,

preProcess = c("center", "scale"),

metric = "ROC")

csl_wtloss.5pct.boot

CI(csl_wtloss.5pct.boot$resample$ROC, ci = 0.95)

set.seed(825)

csl_wtloss.5pct.opt <- train(post.wtloss.5pct.itt ~ Sex+ race+ base.bmi+ OBE.EDEInt2+ ede_ov+ ede_8tot+ comorbid_any+

bdi_tot+ Group+ rapid, data = sc3,

method = "glm",

family = "binomial",

trControl = control_opt,

preProcess = c("center", "scale"),

metric = "ROC")

csl_wtloss.5pct.opt

csl_wtloss.5pct.opt_me <- qt(.975, 99)*(csl_wtloss.5pct.opt$results$ROCSD/sqrt(100))

mean(csl_wtloss.5pct.opt$results$ROC) - csl_wtloss.5pct.opt_me

mean(csl_wtloss.5pct.opt$results$ROC)+csl_wtloss.5pct.opt_me

#-----------10d. OBE ----------

set.seed(825)

csl_obe.cv <- train(post.OBE_pct.itt ~ Sex+ race+ base.bmi+ OBE.EDEInt2+ ede_ov+ ede_8tot+ comorbid_any+

bdi_tot+ Group+ rapid, data = sc3,

method = "glm",

family = "gaussian",

preProcess = c("center", "scale"),

trControl = control.cont_cv)

csl_obe.cv #R2 = .03, RMSE = 37.41

CI(csl_obe.cv$resample$Rsquared, ci = 0.95)

CI(csl_obe.cv$resample$RMSE, ci = 0.95)

set.seed(825)

csl_obe.boot <- train(post.OBE_pct.itt ~ Sex+ race+ base.bmi+ OBE.EDEInt2+ ede_ov+ ede_8tot+ comorbid_any+

bdi_tot+ Group+ rapid, data = sc3,

method = "glm",

family = "gaussian",

preProcess = c("center", "scale"),

trControl = control.cont_boot)

csl_obe.boot #R2 = .01, RMSE = 43.16

CI(csl_obe.boot$resample$Rsquared, ci = 0.95)

CI(csl_obe.boot$resample$RMSE, ci = 0.95)

set.seed(825)

csl_obe.opt <- train(post.OBE_pct.itt ~ Sex+ race+ base.bmi+ OBE.EDEInt2+ ede_ov+ ede_8tot+ comorbid_any+

bdi_tot+ Group+ rapid, data = sc3,

method = "glm",

family = "gaussian",

preProcess = c("center", "scale"),

trControl = control.cont_opt)

csl_obe.opt #R2 = -.04, RMSE = 40.83

csl_obe.opt.r2_me <- qt(.975, 99)*(csl_obe.opt$results$RsquaredSD/sqrt(100))

mean(csl_obe.opt$results$Rsquared) - csl_obe.opt.r2_me

mean(csl_obe.opt$results$Rsquared)+csl_obe.opt.r2_me

csl_obe.opt.rm_me <- qt(.975, 99)*(csl_obe.opt$results$RMSESD/sqrt(100))

mean(csl_obe.opt$results$RMSE) - csl_obe.opt.rm_me

mean(csl_obe.opt$results$RMSE)+csl_obe.opt.rm_me

#-----10e. EDE at post.itt----

set.seed(825)

csl_ede.cv <- train(Total.EDEint.post.itt ~ Sex+ race+ base.bmi+ OBE.EDEInt2+ ede_ov+ ede_8tot+ comorbid_any+

bdi_tot+ Group+ rapid, data = sc3,

method = "glm",

family = "gaussian",

preProcess = c("center", "scale"),

trControl = control.cont_cv)

csl_ede.cv #R2 = .26, RMSE = .75

CI(csl_ede.cv$resample$Rsquared, ci = 0.95)

CI(csl_ede.cv$resample$RMSE, ci = 0.95)

set.seed(825)

csl_ede.boot <- train(Total.EDEint.post.itt ~ Sex+ race+ base.bmi+ OBE.EDEInt2+ ede_ov+ ede_8tot+ comorbid_any+

bdi_tot+ Group+ rapid, data = sc3,

method = "glm",

family = "gaussian",

preProcess = c("center", "scale"),

trControl = control.cont_boot)

csl_ede.boot #R2 = .17, RMSE = .82

CI(csl_ede.boot$resample$Rsquared, ci = 0.95)

CI(csl_ede.boot$resample$RMSE, ci = 0.95)

set.seed(825)

csl_ede.opt <- train(Total.EDEint.post.itt ~ Sex+ race+ base.bmi+ OBE.EDEInt2+ ede_ov+ ede_8tot+ comorbid_any+

bdi_tot+ Group+ rapid, data = sc3,

method = "glm",

family = "gaussian",

preProcess = c("center", "scale"),

trControl = control.cont_opt)

csl_ede.opt #R2 = .23, RMSE = .75

csl_ede.opt.r2_me <- qt(.975, 99)*(csl_ede.opt$results$RsquaredSD/sqrt(100))

mean(csl_ede.opt$results$Rsquared) - csl_ede.opt.r2_me

mean(csl_ede.opt$results$Rsquared)+csl_ede.opt.r2_me

csl_ede.opt.rm_me <- qt(.975, 99)*(csl_ede.opt$results$RMSESD/sqrt(100))

mean(csl_ede.opt$results$RMSE) - csl_ede.opt.rm_me

mean(csl_ede.opt$results$RMSE)+csl_ede.opt.rm_me

#-----10f. wt loss %.itt----

set.seed(825)

csl_wtloss.cv <- train(post.wtloss_pct.itt ~ Sex+ race+ base.bmi+ OBE.EDEInt2+ ede_ov+ ede_8tot+ comorbid_any+

bdi_tot+ Group+ rapid, data = sc3,

method = "glm",

family = "gaussian",

preProcess = c("center", "scale"),

trControl = control.cont_cv)

csl_wtloss.cv #R2 = .11, RMSE = 7.23

CI(csl_wtloss.cv$resample$Rsquared, ci = 0.95)

CI(csl_wtloss.cv$resample$RMSE, ci = 0.95)

set.seed(825)

csl_wtloss.boot <- train(post.wtloss_pct.itt ~ Sex+ race+ base.bmi+ OBE.EDEInt2+ ede_ov+ ede_8tot+ comorbid_any+

bdi_tot+ Group+ rapid, data = sc3,

method = "glm",

family = "gaussian",

preProcess = c("center", "scale"),

trControl = control.cont_boot)

CI(csl_wtloss.boot$resample$Rsquared, ci = 0.95)

CI(csl_wtloss.boot$resample$RMSE, ci = 0.95)

set.seed(825)

csl_wtloss.opt <- train(post.wtloss_pct.itt ~ Sex+ race+ base.bmi+ OBE.EDEInt2+ ede_ov+ ede_8tot+ comorbid_any+

bdi_tot+ Group+ rapid, data = sc3,

method = "glm",

family = "gaussian",

preProcess = c("center", "scale"),

trControl = control.cont_opt)

csl_wtloss.opt #R2 = .07, RMSE = 7.24

csl_wtloss.opt.r2_me <- qt(.975, 99)*(csl_wtloss.opt$results$RsquaredSD/sqrt(100))

mean(csl_wtloss.opt$results$Rsquared) -csl_wtloss.opt.r2_me

mean(csl_wtloss.opt$results$Rsquared)+csl_wtloss.opt.r2_me

csl_wtloss.opt.rm_me <- qt(.975, 99)*(csl_wtloss.opt$results$RMSESD/sqrt(100))

mean(csl_wtloss.opt$results$RMSE) - csl_wtloss.opt.rm_me

mean(csl_wtloss.opt$results$RMSE)+csl_wtloss.opt.rm_me
